# Glucose-Fueled Gated Nanomotors: Enhancing *In Vivo* Anticancer Efficacy via Deep Drug Penetration into Tumors

**DOI:** 10.1021/acsnano.5c03799

**Published:** 2025-05-30

**Authors:** Andrea Escudero, Francisco J. Hicke, Elena Lucena-Sánchez, Sandra Pradana-López, Juan José Esteve-Moreno, Víctor Sanz-Álvarez, Iris Garrido-Cano, Sandra Torres-Ruiz, Juan Miguel Cejalvo, Alba García-Fernández, Paula Díez, Ramón Martínez-Máñez

**Affiliations:** † Instituto Interuniversitario de Investigación de Reconocimiento Molecular yDesarrollo Tecnológico (IDM), Universitat Politècnica de València, Universitat de València, Camino de Vera, s/n., 46022 València, Spain; ‡ Unidad Mixta UPV-CIPF de Investigación en Mecanismos de Enfermedades y Nanomedicina, Universitat Politècnica de València, Centro de Investigación, Príncipe Felipe. Eduardo Primo Yúfera 3, 46012 València, Spain; § CIBER de Bioingeniería, Biomateriales y Nanomedicina, Instituto de Salud Carlos III, 28029 Madrid, Spain; ∥ Biomedical Research Institute INCLIVA, Carrer de Menéndez y Pelayo,4, 46010 Valencia, Spain; ⊥ Biomedical Research Networking Center in Oncology (CIBERONC), 28029 Madrid, Spain; # Department of Clinical Oncology, University Clinical Hospital of Valencia, Av. de Blasco Ibáñez 17, 46010 Valencia, Spain; ∇ Unidad Mixta de Investigación en Nanomedicina y Sensores, Universitat Politècnica de València, IIS La Fe. Av. Fernando Abril Martorell, 106 Torre A 7a planta, 46026 València, Spain

**Keywords:** nanomotors, glucose, platinum-mesoporous silica nanoparticles, controlled-drug delivery, antitumor therapy

## Abstract

Bioinspired nano/micromotors with drug delivery capabilities are emerging tools with the promising potential to treat numerous diseases. However, some major challenges must be overcome before reaching real biomedical applications. Above all, it is necessary to design engines that employ biocompatible and bioavailable fuels to induce efficient propulsion in biological environments. In addition, ideal nanomotors should also be capable of delivering the cargo on-command using selected stimuli. To tackle these challenges, we herein present the design and evaluation (both *in vitro* and *in vivo*) of a glucose-driven gated Janus nanomotor that performs on-demand anticancer drug delivery to treat solid tumors. The motor’s nanoarchitectonics is based on the anisotropic conjunction of catalytic platinum nanodendrites (PtNds) and a mesoporous silica nanoparticle (acting as a nanocontainer for anticancer drug doxorubicin) capped with enzyme glucose oxidase (GOx). Autonomous nanomotor movement is achieved thanks to two catalytic components, GOx and PtNds, in a hybrid cascade reaction: GOx transforms glucose to give H_2_O_2_ that is subsequently catalyzed by PtNds into H_2_O and O_2_. Besides, gatekeeper moieties (GOx) respond to the presence of intracellular proteases, which induces doxorubicin delivery. Biological experiments with the nanomotor are carried out in cancer cell cultures, three-dimensional (3D) tumor models (spheroids), *in vivo* and in patient-derived organoids (PDOs). A strong anticancer effect is found and attributed to the synergistic combination glucose-induced propulsion, controlled drug delivery, elimination of glucose (by GOx), ROS production (H_2_O_2_ generation by GOx) and hypoxia reduction (O_2_ generated by PtNds). Taken together, this study advances the engineering of endogenously fueled nanomotors for *in vivo* operation and provides insights into the application of active particles in cancer therapy toward clinical application.

## Graphical abstract

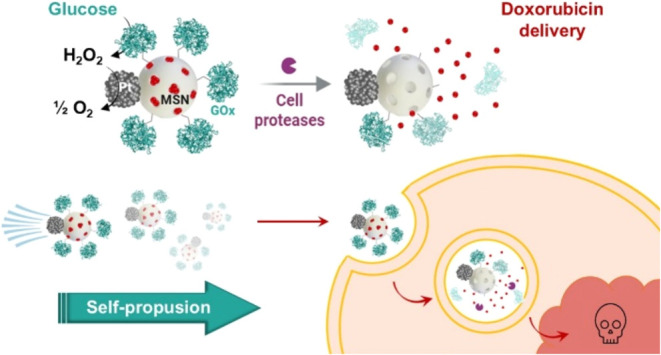

## Introduction

One of the most promising research directions in nanotechnology is the development of micro- and nanobots for advanced applications in areas like precision medicine,^1,2^ biotechnology^3^ and sensing.^4^ Nanobots can be fabricated following nanoarchitectonic approaches by assembling abiotic and/or biological nanocomponents, and are seen as a potential avenue for designing a new generation of smart nanodevices for biomedical applications,^5–7^ such as localized drug delivery.^8,9^ Ideal nanobots for such biomedical application should be endowed with self-propulsion and the capability to read molecular information from the environment and to act accordingly.^10^ In addition, nanobots with self-propulsion capabilities could contribute to solve one of the main hindrances of conventional drug-delivery systems: passing through biological barriers (BBs), such as the extracellular matrix (ECM) in solid tumors.^11–13^

The motion of micro/nanobots can be induced by several approaches that range from physically powered (i.e., using acoustic,^14^ magnetic,^15^ and electric^16^ stimuli as energy sources) to chemically powered. Whereas physically powered micro/nanomotors require an external power source, chemically powered ones employ the transformation of the chemical fuels available in their environment for their propulsion. Motor nanoarchitectonics is usually based on the asymmetric distribution of catalysts on a particle chassis, which leads to the nonsymmetric accumulation of reaction products that induces enhanced motion.^17^ In particular, micro/nanomotors have been described using the catalytic decomposition of chemicals, such as hydrogen peroxide (H_2_O_2_) or hydrazine, by active metallic or enzymatic units.^18^ Among metallic motors, those based on platinum (Pt)^19,20^ are prominent, while those employing catalase as enzymatic motor^21,22^ are the most widespread. In both cases, Pt and catalase catalyze the decomposition of H_2_O_2_ into water (H_2_O) and oxygen (O_2_). However, H_2_O_2_ is a toxic fuel and these H_2_O_2_-propelled micro/nanomotors typically require an external supply of high concentrations of exogenous H_2_O_2_ (that causes damage to cellular structures and oxidative stress), which hinders implementing H_2_O_2_-based engines into living organisms.^23^ Therefore, finding other biocompatible and bioavailable fuels (i.e., unharmful to cells and naturally present in the body at relevant concentrations) is needed. Recent advances in this direction include the design of water-, gastric acid-, amino acids-, glucose- or urea-driven motors.^24–28^ In this regard, one example is the nanomotor consisting of mesoporous silica nanoparticles (MSNs) and urease described by Ruiz-Gonzalez et al.^29^ Authors found that the nanobots navigated in a coordinated manner throughout the bladder exhibiting swarming behavior that induced beneficial fluid mixing to promote drug release.

However, despite these advances, three major challenges can still be highlighted in the development of micro/nanomotors for drug delivery applications: (i) the design of engine’s that employs biocompatible and bioavailable fuels to induce efficient propulsion in biological environments; (ii) the introduction of controlled cargo release capabilities to minimize premature cargo leakage; and (iii) demonstration of applicability in complex living models of disease. In this scenario, Table S1 summarizes some of the few examples published to date on chemically driven nanomotors for *in vivo* cancer treatment. A comparison of the material design, driving system, model complexity and treatment effect is also shown. For instance, Zhong et al. designed platinum nanomotors driven by H_2_O_2_ to enhance the penetration of chemotherapeutics to deep tumor areas. These nanodevices showed a sensitization effect to radiotherapy by improving its rate of tumor growth inhibition in a breast cancer model.^30^ Besides, Yu et al. described the use of cationic gold nanoclusters decorated with glucose oxidase (GOx) and catalase for the reconstruction of the tumor microenvironment. These glucose-propelled nanomotors delivered a hexokinase-2 siRNA to breast cancer cells achieving tumor volume and metastasis reduction.^31^

However, despite the important progress carried out, there are still efforts to be made. The main limitations of these nanodesigns remain in the requirement for high concentrations of fuel to exhibit enhanced diffusion and the lack of cargo delivery or control in its release. In most nanodevices, payload release occurs by simple diffusion instead of upon the recognition of specific stimuli. In conclusion, addressing all the challenges outlined above by a single nanodesign is no trivial matter and has not been effectively achieved to date.

To tackle them, here we report herein the preparation and evaluation of glucose-fueled gated Janus (snowman-type) nanomotors capable of controlled drug release and its enhanced antitumoral activity *in vivo*. The Janus nanoarchitectonics we selected presents two faces with different chemical properties and functionalities, i.e., MSNs and platium nanodendrites (PtNds). Among all drug carriers, MSNs offer advantageous properties such as their three-dimensional (3D) porous network with high loading capacity, biocompatibility, low cost, and the possibility to functionalize them with molecular gates (also called gatekeepers or nanovalves). These gatekeepers act as stimuli-responsive caps that prevent cargo release until a certain physical, chemical or biochemical stimulus is applied.^32,33^ In our system, the MSN part of the nanomotor is responsible for transporting and controlling the release of an antitumor drug (i.e., doxorubicin). For on-command payload delivery, the surface of MSNs is functionalized with amine groups for the covalent attachment of enzyme glucose oxidase (GOx) via the formation of amide bonds, resulting in a protease-sensitive gatekeeper. Autonomous nanomotor movement is achieved thanks to their two catalytic components, GOx and PtNds, in a hybrid cascade reaction: first, GOx performs the enzymatic transformation of glucose into gluconic acid and H_2_O_2_ on the silica surface; and subsequently, PtNds catalyze the decomposition of the H_2_O_2_, generated locally by GOx enzyme, into H_2_O and O_2_. Moreover, the anisotropic nanoarchitecture of nanobots favors the localized generation of H_2_O and O_2_ on the PtNds face, inducing propulsion probably by a self-phoretic mechanism.^34^

A scheme of the doxorubicin-loaded and GOx-functionalized Janus nanomotor (NM_Doxo‑GOx_) and the expected antitumor performance is shown in Scheme 1A. We envisioned that the nanomotor would display active diffusive behavior in response to glucose present in the tumor microenvironment, which would result in deeper and enhanced drug delivery in a tumor model. Moreover, the nanomotor will be able to eliminate glucose (by GOx), produce ROS (H_2_O_2_ generated by GOx) and alleviate hypoxia (O_2_ generated by PtNds). All together is expected to induce enhanced cancer cell death. We report below the investigation of nanoparticle preparation and characterization, controlled cargo release experiments, motility studies and biological evaluations both *in vitro* and *in vivo*.

**Scheme 1:**
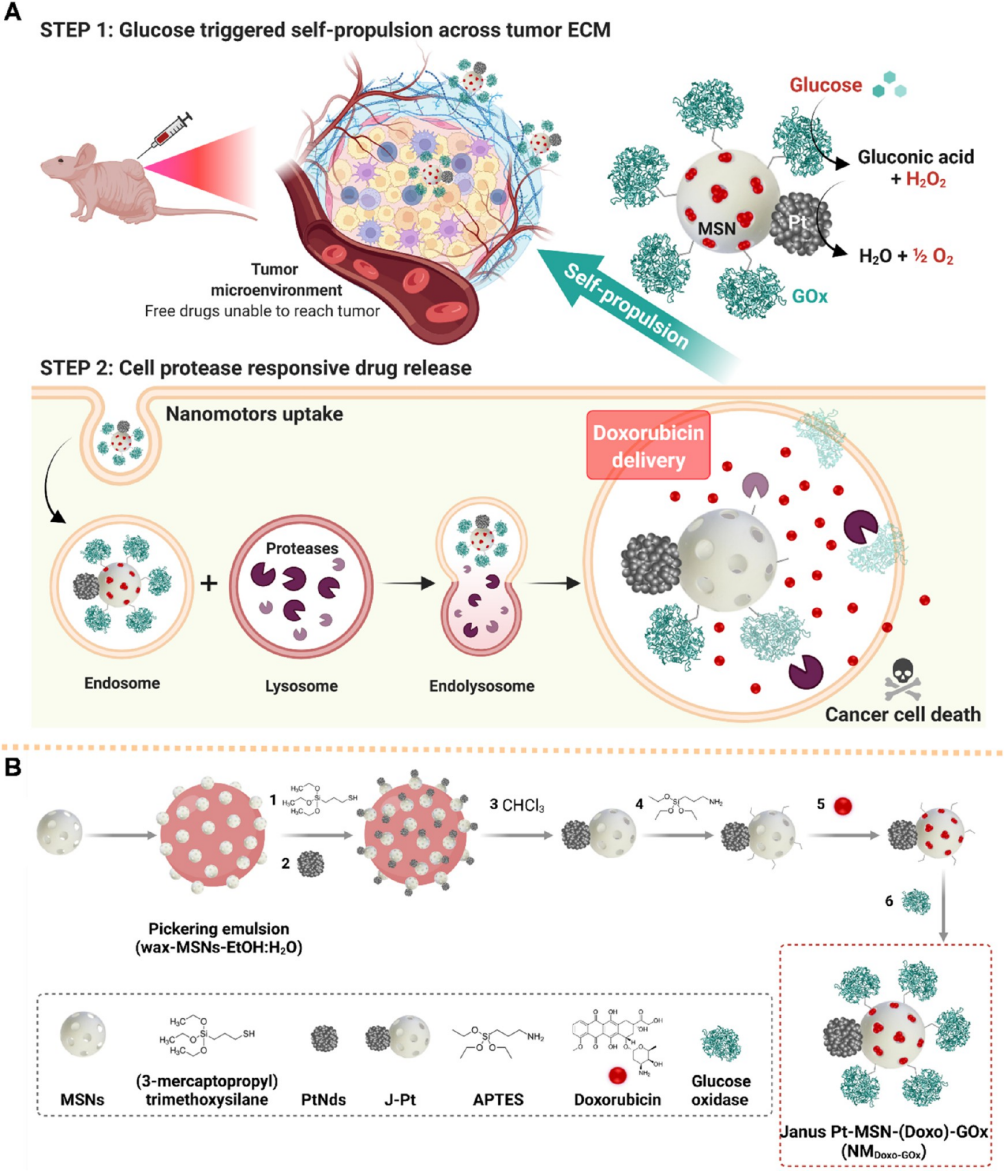
(A) Illustration of the Antitumor Performance of the NM_Doxo‑GOx_ Nanomotor; Step 1: Glucose Induces Self-propulsion of Nanomotors *via* the Sequential Catalytic Activity of GOx and PtNds; Step 2: Nanomotors Release Doxorubicin in Response to Lysosomal Proteases; Resulting in Cancer Cell Elimination; Created with Biorender.com; and (B) Representation of the Synthetic Procedure to Obtain NM_Doxo‑GOx_

## Results and Discussion

### Design, Synthesis, and Characterization of Nanomotors

Janus Pt-MSNs (J-Pt) were synthesized by the toposelective combination of PtNds and MSNs (Scheme 1B and Experimental Section for details).^35–37^ Briefly, MSNs were embedded at the interface of an oil/water Pickering emulsion, exposing one side to the aqueous phase. The MSNs surface was reacted with (3-mercaptopropyl)­trimethoxysilane and, thereafter, with PtNds obtaining J-Pt. To obtain the final nanomotor (NM_Doxo‑GOx_), the MSNs face was loaded with doxorubicin, decorated with (3-aminopropyl)­triethoxysilane and finally capped with GOx through the formation of amide bonds. GOx acts as (i) a component of the nanomotor, enzymatically transforming glucose into H_2_O_2_ and (ii) a protease-sensitive gatekeeper controlling cargo release.^38^ PtNds face was selected as the second element of the system due to its large rough surface, which translates into a high activity for catalytic H_2_O_2_ reduction.^39–42^

We also synthesized control nanoparticles, in which essential components were removed or substituted to impair their function. We specifically prepared control nanoparticles devoid of the drug (NM_GOx_ without doxorubicin) and control nanoparticles devoid of motion capability. For the latter, we prepared two different controls (i) by replacing the GOx enzyme (which initiates the catalytic cascade reaction) with the BSA protein, which only acts as gatekeeper (NM_Doxo‑BSA_) (ii) or by inactivating GOx enzyme by heat (NM_Doxo‑GOx‑IN_). This thermal process results in GOx losing its structure and, therefore, its catalytic activity, being unable to transform glucose into H_2_O_2_. In addition, NM_GOx_ and NM_GOx‑IN_ covalently labeled with a fluorophore (PA, Figure S1) were prepared for visualization by confocal laser scanning microcopy (CLSM). The PA fluorophore was specifically designed and prepared for this study because it is not degraded by H_2_O_2_ generated by the GOx enzyme. Finally, we also included a control group consisting of all the active components of the final nanoparticle NM_Doxo‑GOx_ (doxorubicin, PtNds and GOx) but not assembled (NA). A scheme of all prepared nanoparticles is summarized in Table S2.

Characterization of the prepared nanoparticles was carried out using different techniques. The morphology of the starting MSNs, PtNds, J-Pt and NM_GOx_ was confirmed by high-resolution transmission electron microscopy (HR-TEM) and field emission scanning electron microscopy (FE-SEM), as shown in Figure 1A and Figures S2B and S3. The J-Pt nanoparticles had a “snowman-like” morphology with a diameter of 124.2 ± 20.5 nm due to the binding of individual nanoparticles of PtNds and MSNs (Figures 1Ai and S2A). On the one hand, the PtNds face is formed by the fusion of multiple Pt seeds with a diameter of ca. 2.5 nm organized in a spherical nanoparticle of 37 ± 8 nm. As shown in Figure 1Aii, PtNds presents a crystalline nanostructure with lattice fringes interplanar spacing of 2.2 and 1.9 Å for the (111) and (200) face-centered cubic planes (fcc),^43,44^ as further confirmed by powder X-ray diffraction (PXRD) (Figure S4A). On the other hand, the MSNs face shows a hexagonal porous MCM-41-like structure with a spherical shape and a diameter of 92 ± 16 nm. Likewise, HR-TEM images show that the mesoporous order in MSNs was not altered after the PtNds attachment and functionalization (Figure S4B). Additionally, scanning transmission electron microscopy coupled with energy dispersive X-ray spectroscopy (STEM-EDX) of NM_GOx_ allowed the construction of a map showing the atomic composition (wt %) (Figure 1B). O and Si atoms were the most abundant (44.6 and 30.4%, respectively) and were attributed to the MSNs face. Pt atoms were found on the opposite face and were quantified as 17.5% of the total composition. A similar percentage was obtained by inductively coupled plasma mass spectrometry analysis (ICP-MS), which indicated that there are 200 μg of Pt per mg of J-Pt (20 wt %). N atoms (7.5%) assigned to enzyme immobilization were also detected.

**Figure 1:**
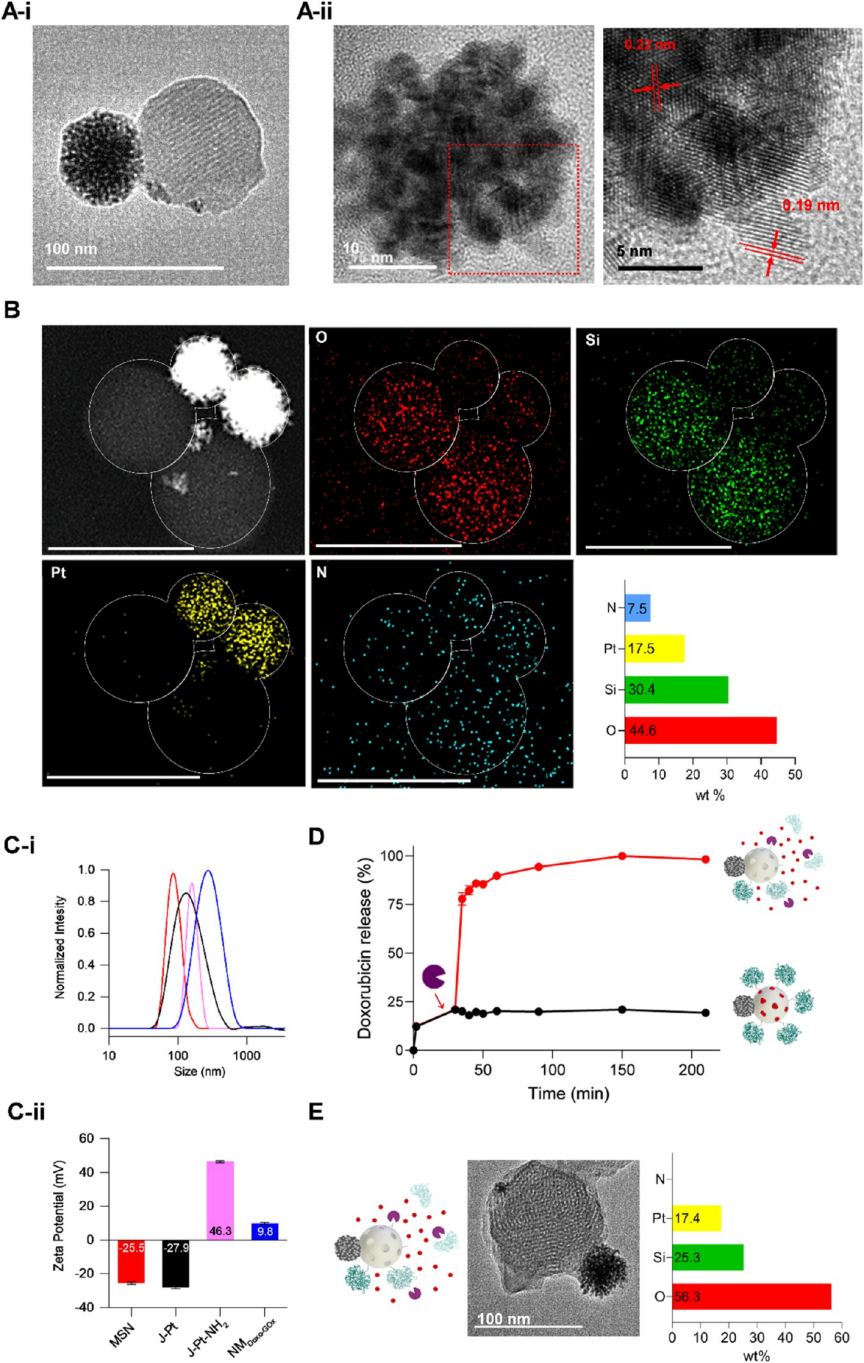
Structural characterization and controlled cargo release. (A-i) HR-TEM image of J-Pt showing the hexagonal porous structure of MSNs and the successful conjugation with PtNds in a single anisotropic nanoparticle with a snowman-like morphology: (A-ii) HR-TEM image of PtNds depicting the presence of the crystalline faces of PtNds with interplanar distances of 2.2 and 1.9 Å on the (111) and (200) planes, respectively. (B) STEM-EDX analysis of NM_GOx_. Elemental mapping confirms the presence of O (red, 44.6%), Si (green, 30.4%), Pt (yellow, 17.5%) and N (blue, 7.5%) atoms, (scale bar 100 nm). (C) Changes in the hydrodynamic diameter determined by DLS (C-i) and the ζ-potential (C-ii) during the different steps followed for the synthesis of NM_Doxo‑GOx_, (red, MSN; black, J-Pt; pink, J-Pt-NH_2_; blue, NM_Doxo‑GOx_). (D) Normalized doxorubicin release profile from NM_Doxo‑GOx_ monitored by fluorescence spectroscopy (λ_em_, 555 nm; λ_ex_, 470 nm) in the presence (red) and absence (black) of proteases from Streptomyces griseus (final concentration of 1 mg mL^–1^) added at 30 min (data represent the mean ± SD, *n* = 3). (E) Study of composition and morphology by HR-TEM and STEM-EDX after the controlled release of doxorubicin by proteases from NM_Doxo‑GOx_. The atomic quantification by STEM-EDX confirmed the presence of O (red, 57.3%), Si (green, 25.3%), Pt (yellow, 17.4%) and the absence of N atoms in agreement with the degradation of GOx by protease enzymes.

The N_2_ adsorption–desorption isotherms also corroborated the presence of the porous network in MSNs, which was preserved in J-Pt. In both isotherms, an acute adsorption step corresponding to the N_2_ condensation inside the mesopores was observed at values of 0.1–0.3 *P*/*P*_0_ (Figure S5). In contrast, the N_2_ isotherm for NM_GOx_ showed a significant reduction in the absorption process, indicating pore blocking with GOx. The application of the Barret–Joyner–Halenda model (BJH)^45^ resulted in the determination of the pore diameter and volume values of 2.69 nm and 0.82 cm^3^ g^–1^ for MSNs, 2.30 nm and 0.14 cm^3^ g^–1^ for J-Pt, and nearly zero for NM_GOx_ (Table S3). Moreover, the application of the Brunauer–Emmett–Teller model (BET)^46^ allowed calculation of the total specific surface area: 997.9 m^2^ g^–1^ for MSNs, 551.2 m^2^ g^–1^ for J-Pt, and 49.6 m^2^ g^–1^ for NM_GOx_ (Table S3, Supporting Information). In addition, the presence of GOx in NM_GOx_ was also supported by Fourier transform infrared (FTIR) studies, where characteristic IR absorption bands of amide I bond of proteins (1651 cm^–1^), C–N bond (1543 cm^–1^) and CH_2_ groups (2975 cm^–1^) were found (Figure S6). Quantification of the GOx enzyme bound to the nanomotor NM_GOx_ was calculated by bicinchoninic acid assay (BCA)^47^ and resulted in 108.5 μg per mg of NM_GOx_ (Table S4). The thermogravimetric analysis (TGA) supported these results (Figure S7).

Dynamic light scattering (DLS) and ζ-potential analysis allowed to follow size and surface charge changes during the synthesis of the nanoparticles. The hydrodynamic diameter increased from 146 ± 3 nm in MSNs to 168 ± 2 nm in J-Pt due to the linking of PtNds. Upon doxorubicin loading and the immobilization of the capping GOx in NM_Doxo‑GOx_, the size increased up to 271 ± 3 nm (Figure 1Ci). Regarding the ζ-potential, typical negative values of MSNs due to the presence of silanol groups (−25.5 mV) increased in J-Pt modified with APTES (J-Pt-NH_2_, 46.3 mV). However, the ζ-potential value decreased to slightly positive values in NM_Doxo‑GOx_ (9.8 mV) due to the attachment of GOx, which is negatively charged at a neutral pH (IP = 4.2) (Figure 1Cii).^48–50^ Similar studies were also performed for control nanoparticles (Figure S8).

### On-Command Protease-Triggered Delivery of Doxorubicin

As detailed above, J-Pt mesopores were loaded with doxorubicin and the system was capped by GOx to give NM_Doxo‑GOx_. The drug doxorubicin is expected to be released following the mechanism depicted in Scheme 1A. Thus, after cellular internalization of the nanoparticles, the high lysosomal protease activity in cancer cells is expected to cause hydrolysis of GOx, inducing unblocking of the mesopores and the concomitant release of the drug into the medium by a favorable concentration gradient.

To study the delivery process, controlled delivery of doxorubicin^51^ from NM_Doxo‑GOx_ in response to proteases from Streptomyces griseus was evaluated by fluorescence spectroscopy. As can be observed in Figure 1D, NM_Doxo‑GOx_ remained capped, retaining most of the drug inside the porous structure in the absence of proteases (black curve). Conversely, enhanced doxorubicin delivery was found in the presence of the enzyme with a maximum delivery achieved at ca. 150 min (red curve). Under the conditions tested, payload release is attributed to the specific hydrolysis of the GOx gatekeeper by proteases.^52^ We also investigated the protease threshold needed to trigger doxorubicin release. As observed in Figure S9A, 0.05 mg mL^–1^ of protease are sufficient to cause a slight increase in doxorubicin release, with significant release obtained when using 0.1 mg mL^–1^. Total doxorubicin loaded in NM_Doxo‑GOx_ was determined by a forced release assay, amounting to 67 μg per mg of NM_Doxo‑GOx_ (eq 1, and Table S4). In a subsequent step, we also evaluated the controlled release of doxorubicin from NM_Doxo‑GOx_ using purified lysosomal extract with the aim of reproducing the environment that nanoparticles will face in lysosomes. As shown in Figure S9B, lysosomal extract also triggers doxorubicin release. The results demonstrate that NM_Doxo‑GOx_ delivers doxorubicin in response to an intracellular proteolytic stimulus.

Finally, HR-TEM studies, after the protease-mediated controlled release of doxorubicin from NM_Doxo‑GOx_, demonstrated that the MSNs-PtNds scaffold remained unaffected (Figure 1E). Moreover, atomic quantification by STEM-EDX (Figure 1E) showed similar values for O, Si and Pt (57.3% of O, 25.3% of Si and 17.4% Pt atoms) to those of NM_Doxo‑GOx_ whereas a reduction of N atoms from 7.5% to undetectable values was also found, which agrees with the degradation of GOx by protease enzymes.

### Analysis of Glucose-Induced Motility

The propulsion strategy followed in the design of NM_Doxo‑GOx_ started with (i) the enzymatic transformation of glucose (an endogenous fuel present in the tumor microenvironment)^52^ into H_2_O_2_ by the GOx enzyme (β-d-glucose + O_2_ → d-glucono-1,5-lactone + H_2_O_2_), followed by (ii) the catalysis of the generated H_2_O_2_ into O_2_ and water (H_2_O_2_ → H_2_O + 1/2 O_2_) by the PtNds face (Scheme 1A). To demonstrate the propulsion mechanism, we first tested the catalytic GOx activity on NM_Doxo‑GOx_ by a spectrophotometric assay (Figure S10). From this assay, the activity of the immobilized GOx enzyme was calculated to be 323 U per g of NM_Doxo‑GOx_, which corresponds to 0.3 U per mg of GOx (based on BCA protein quantification). Subsequently, the H_2_O_2_-reducing activity of the PtNds face on NM_Doxo‑GOx_ was demonstrated by ultraviolet–visible (UV–vis) spectrophotometry. The nanoparticle displayed a high peroxidase activity following a Michaelis–Menten-like enzyme kinetics (Figure S11) with an apparent affinity constant (*K*_M_) for H_2_O_2_ of 13 mM (eq 6). Moreover, the catalytic efficiency to transform H_2_O_2_ in terms of turnover rate (*K*_cat_) was calculated to be 5.2 × 10^5^ s^–1^ (eq 7 and Table S5), a value almost 150-fold higher than horseradish peroxidase (HRP).^53,54^ Likewise, to further demonstrate the NM_Doxo‑GOx_ catalytic activities we quantitatively analyzed glucose consumption, H_2_O_2_ production, and dissolved O_2_ levels during the reaction. The results (Figure S12) demonstrated that NM_Doxo‑GOx_ induced rapid glucose depletion (measured via phenol-sulfuric acid assay) within 30 min under physiological glucose concentrations (5 mM). This glucose consumption was followed by the efficient decomposition of the generated H_2_O_2_ (quantified by Ampliflu Red/HRP assay), confirming the critical role of PtNds in the process. Notably, this behavior was absent in NM_Doxo‑GOx‑IN_, emphasizing the necessity of the integrated Janus architecture to transform glucose into H_2_O_2_, and decompose H_2_O_2_ to promote propulsion. Also, dissolved O_2_ levels generated by NM_Doxo‑GOx_ were monitored with an oximeter over time (60 min) in response to different glucose concentrations (from 0 to 15 mM) under hypoxic conditions. An increase in dissolved O_2_ was observed at all glucose concentrations, reaching 3 mg L^–1^ at 15 mM. These experiments demonstrate that NM_Doxo‑GOx_ transforms glucose into H_2_O_2_, and that decomposes H_2_O_2_ to give H_2_O and O_2_.

After confirming the catalytic capabilities of NM_Doxo‑GOx_, we characterized the movement as a function of the glucose concentration (Figure 2). The glucose concentrations tested (0–25 mM) in the experiments throughout the work were selected based on usual endogenous glucose levels in blood and tissues (4–7 mM)^55^ and the usual level of the cell medium in which some of the *in vitro* assays (*vide infra*) were carried out (25 mM).^56,57^ The range studied also agrees with bibliographic data, which points that the local levels of this metabolite are prominent in tumors.^58^ This is attributed to the increased transport of glucose from the circulatory system as it is an essential nutrient that provides energy (Warburg effect) and precursor for biosynthetic processes in cancer cells.^59,60^

**Figure 2:**
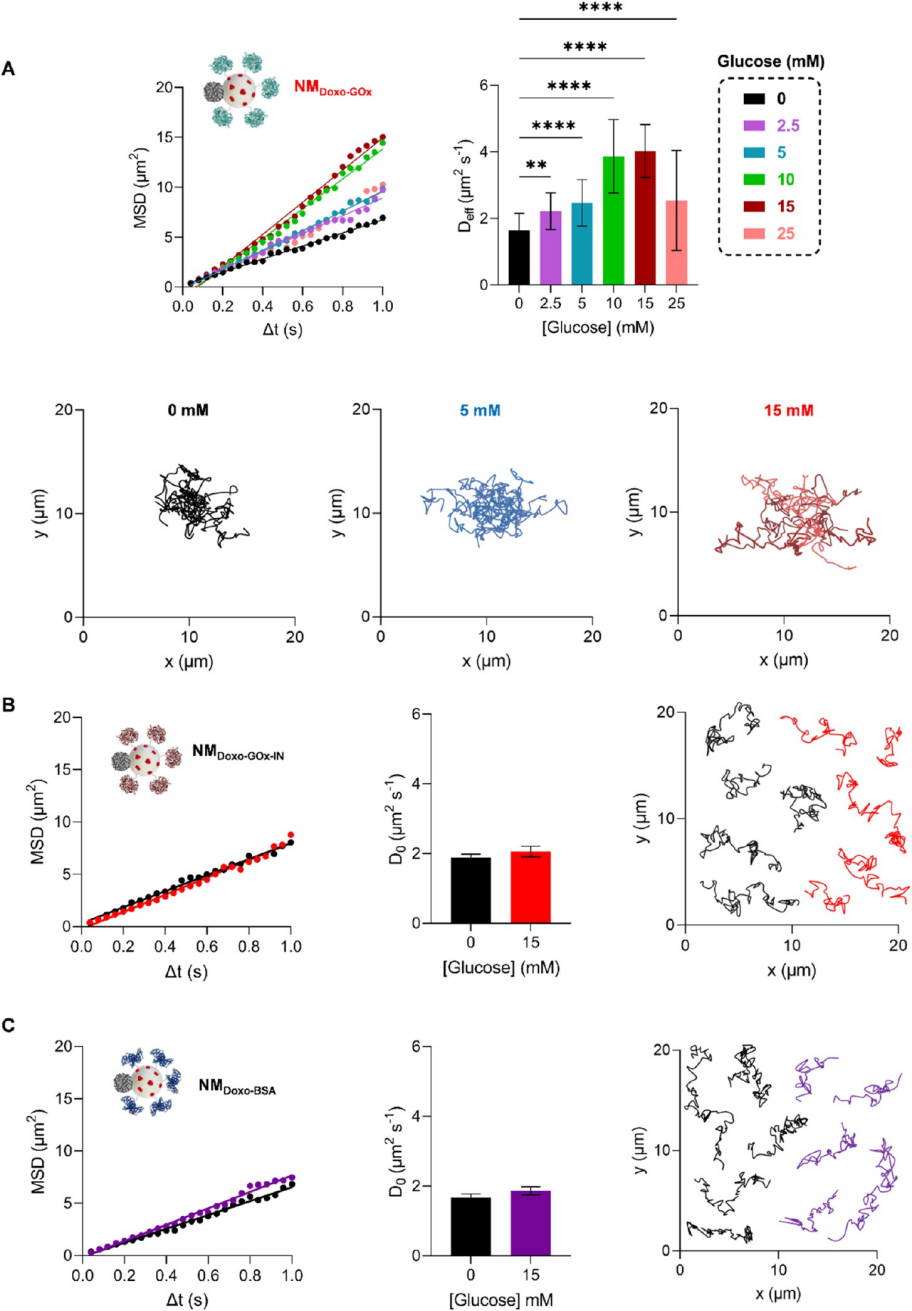
Analysis of nanomotors’ motion in response to glucose: NTA evaluation of NM_Doxo‑GOx_ (A) and the control nanoparticles NM_Doxo‑GOx‑IN_ (B) and NM_Doxo‑BSA_ (C). (A) From left to right: average mean MSD vs Δ*t* at different glucose concentrations (0, black; 2.5 mM, purple; 5 mM, blue; 10 mM, green; 15 mM, dark red and 25 mM, pink), *D*_eff_ or *D*_0_ values calculated from the MSD plots and 5 representative spatial trajectories at 0 (black), 5 (blue) and 15 mM (red) of glucose. (B–C) From left to right: MSD vs Δ*t*, *D*_0_ values calculated from the MSD plots and 5 representative spatial trajectories of NM_Doxo‑GOx‑IN_ and NM_Doxo‑BSA_ at 0 and 15 mM. of glucose (0 mM: black; 15 mM: red in NM_Doxo‑GOx‑IN_ and purple in NM_Doxo‑BSA_). The differences between 0 and 15 mM in control nanoparticles were not statistically significant and, therefore, were not indicated. Data represents the media ± SEM of 50 nanodevices with a size between 250 and 350 nm. Statistical significance was determined by a one-way analysis of variance (ANOVA) Test (** *p* < 0.01, **** *p* < 0.0001).

To record the nanoparticle’s mobility, we employed a Nanosight NS300 instrument, the nanoparticle tracking analysis software (NTA) and an in-house developed R code to analyze data. Briefly, the *x* and *y* positions of each nanoparticle were used to calculate their mean square displacement (MSD) (eq 9). Plotting the MSD values vs time increment (Δ*t*) allowed obtaining a linear correlation from which the diffusion coefficients (*D*) of nanomotors (*D*_0_ for passive nanodevices and effective diffusion coefficient, *D*_eff_ in the presence of glucose) were calculated (eq 10). NM_Doxo‑GOx_ showed a Brownian motion in absence of glucose and short tracking paths with a *D*_0_ of 1.6 ± 0.1 μm^2^ s^–1^, value that is an agreement with the theoretical diffusion according to the Stokes–Einstein equation for nanoparticles of a size of 271 nm (1.6 μm^2^ s^–1^) (Figures 2A and S13). In contrast, NM_Doxo‑GOx_ exhibited enhanced diffusivity in the presence of glucose (Videos S1 and S2). *D*_eff_ for NM_Doxo‑GOx_ increased from 2.2 ± 0.1 μm^2^ s^–1^ at 2.5 mM glucose to a maximum of 4.0 ± 0.1 μm^2^ s^–1^ at 15 mM of fuel, displaying enhanced random spatial trajectories (Figures 2A and S13). However, at 25 mM of glucose *D*_eff_ (2.5 ± 0.2 μm^2^ s^–1^) decreased to values like those reached at 5 mM of fuel (2.5 ± 0.1 μm^2^ s^–1^), most likely caused by the saturation of the enzyme activity at high substrate concentrations.

To verify the proposed mechanism, we investigated whether the replacement or inactivation of the first element that endows the nanomotor with self-propulsion (i.e., GOx) led to the loss of motion capability. As a summary, Table S6, Figures 2 and S14–S15 show *D*_0_ and *D*_eff_ values of the complete nanomotor (NM_Doxo‑GOx_) and those for NM_Doxo‑GOx‑IN_ (in which GOx is inactivated by heat) and NM_Doxo‑BSA_ (in which GOx was replaced by the protein BSA) at 0 and 15 mM of glucose. Only NM_Doxo‑GOx_ showed a significant diffusion coefficient increase (2.4-fold) because of the addition of the glucose, while no significant increase in *D*_0_ for NM_Doxo‑GOx‑IN_ and NM_Doxo‑BSA_ was observed (Figures S14–S16). These experiments confirm the key role of GOx in NM_Doxo‑GOx_ to achieve glucose-induced autonomous movement.

In addition, given that the Pt face of NM presents a high catalytic activity for H_2_O_2_ decomposition, and that in tumors H_2_O_2_ can be present due to the overproduction of ROS (concentration around 100 μM),^61^ we evaluated whether the addition of H_2_O_2_ (at 100 μM concentration) resulted in an increase in the glucose-induced motion. To carry out this assay, a combination of glucose and H_2_O_2_ was used as fuel (glucose 5 mM + H_2_O_2_ 100 μM). The results obtained (Figure S17) show that there are no D_eff_ differences when adding or not H_2_O_2_ at this concentration.

These results indicated that the sequential decomposition of glucose and H_2_O_2_ on the anisotropic surface of the Janus snowman-type nanomotors generated a driving force following probably a self-diffusiophoresis mechanism.^62–64^ Altogether, these findings demonstrated that NM_Doxo‑GOx_ shows (i) a controlled delivery of the anticancer drug doxorubicin triggered by the presence of proteases; and (ii) diffusive propulsion by a catalytic cascade process in the presence of glucose. Finally, we demonstrated that NM_Doxo‑GOx_ can be stored for at least 60 days without decomposing or significant loss of functionalities when suspended in PBS and 4 °C (Figure S18). Thus, GOx activity in stored NM_Doxo‑GOx_ was maintained for 60 days with only a 20% reduction at the end of the period, also showing similar *D*_eff_ values at days 0 and 60. Doxorubicin content at day 60 (52 μg per mg of NM_Doxo‑GOx_) was similar to that found at day 0 (Table S4). Moreover, TEM images at day 60 demonstrated that the porous structure of the MSN face and the crystalline nanostructure of the PtNds face remained unaltered.

### Cytotoxicity Studies

After confirming the specific release of doxorubicin in response to proteases (Figures 1D and S9), the cell uptake of NM_Doxo‑GOx_ was confirmed by TEM (Figure S19), CLSM and flow cytometry (Figure S20). Moreover, cytotoxicity studies *in vitro* in two-dimensional (2D) and three-dimensional (3D) cancer cell culture were carried out.

The cytotoxic activity of NM_Doxo‑GOx_ was assessed in the presence and absence of glucose in a 2D HeLa cell culture (Figure 3A). Cell viability studies performed in the presence of glucose revealed an increased therapeutic effect of NM_Doxo‑GOx_, which resulted in effective cancer cell death. NM_Doxo‑GOx_ (Figure 3Aii, red stripped) notably reduced cancer cell viability to ca. 50% at the very low dose of 5 μg mL^–1^ in the presence of glucose. In contrast, cell viability remained close to 100% in NM_Doxo‑GOx_ treated cells in the absence of fuel (Figure 3Aii, red). Besides, higher doses of NM_Doxo‑GOx_, above 50 μg mL^–1^, caused a large decrease in cancer cell viability (approximately 0% in the presence of glucose) (Figure S21A). The cytotoxicity of NM_Doxo‑GOx_ was also demonstrated in 2D models of breast cancer and melanoma (Figure S22). Further viability experiments also proved that control nanoparticles without enhanced motion NM_Doxo‑GOx‑IN_ (Figure 3Aii, black) and NM_Doxo‑BSA_ (Figure 3Aii, purple), were not toxic to tumor cells at the same concentrations in both, the presence and absence of glucose. In addition, NM_GOx_ (unloaded nanomotors; Figure 3Aii, blue), NA (unassembled nanomotor; Figure 3Aii, orange) and PtNds (Figure S23) were neither toxic to HeLa cells at the low concentrations tested. We also confirmed that the enzymatic cascade activity of GOx and PtNds in NM_GOx_ in the presence of glucose generated reactive oxygen species (ROS) inducing oxidative stress in cells (Figures S21B and S24).^65–68^ Taken together, these experimental data suggest that increased motility of gated nanomotors positively impacts cancer cell killing via the synergistic combination of anticancer drug delivery and intracellular ROS production, which causes cell damage by oxidative stress.

**Figure 3:**
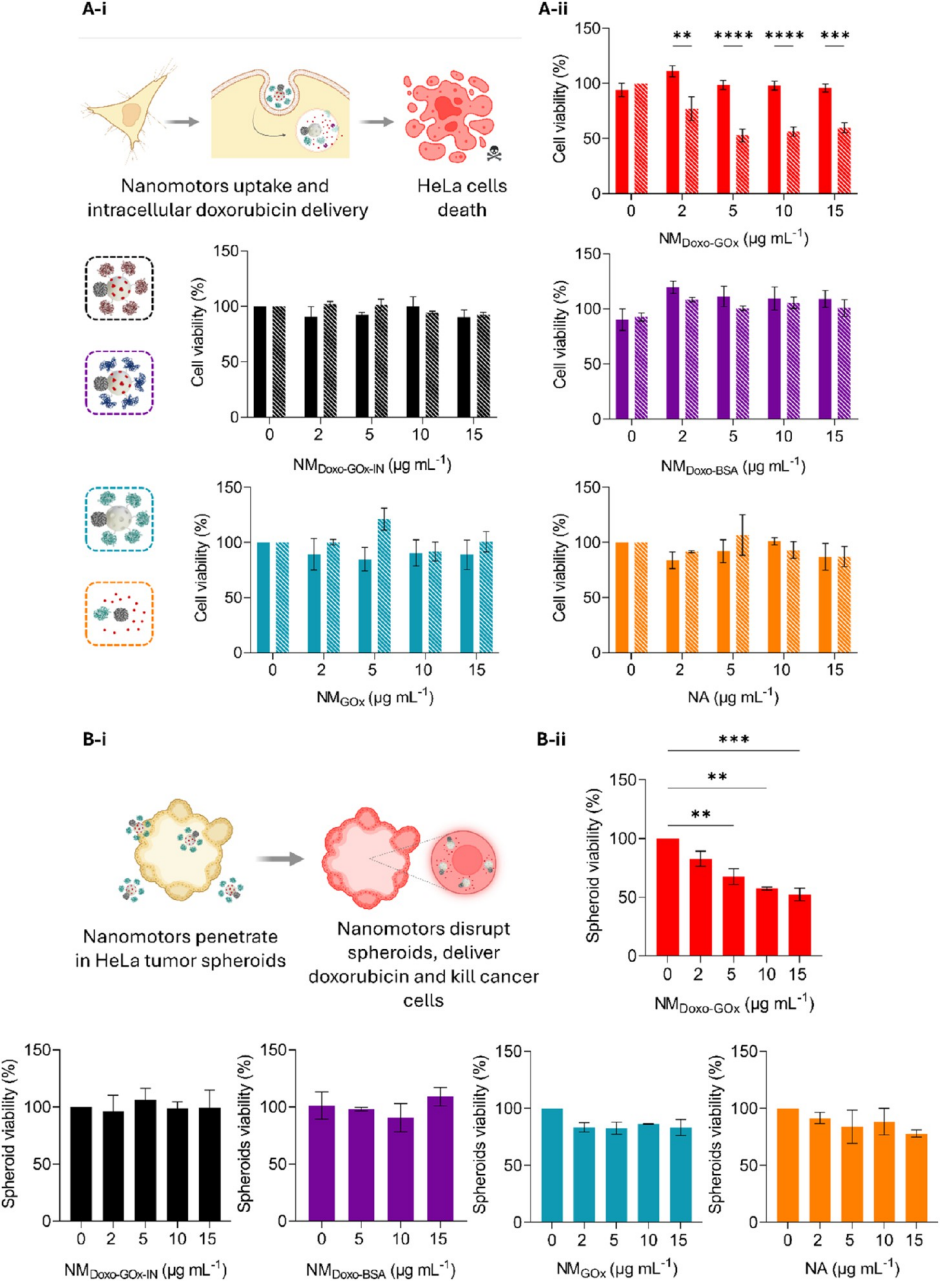
Evaluation of the anticancer capacities *in vitro*. (A-i) Scheme of nanomotors’ performance in HeLa cells in 2D culture. Created with Biorender.com. (A-ii) Cell viability assays. HeLa cells were treated with different concentrations of NM_Doxo‑GOx_ (red), NM_Doxo‑GOx‑IN_ (black), NM_Doxo‑BSA_ (purple), NM_GOx_ (blue), and NA (orange) in the absence or presence of glucose 25 mM (striped) for 24 h. Data are presented as mean ± SEM, *n* = 3. Statistical significance was determined by a two-way ANOVA Test (* *p* < 0.05, ** *p* < 0.01, *** *p* < 0.001, **** *p* < 0.0001). (B-i) Scheme of nanomotors’ performance in 3D HeLa tumor spheroids. Created with Biorender.com. (B-ii) Spheroids viability assays. HeLa spheroids were incubated for 24 h with different concentrations of NM_Doxo‑GOx_ (red), NM_Doxo‑GOx‑IN_ (black), NM_Doxo‑BSA_ (purple), NM_GOx_ (blue), and NA (orange). Data are expressed as mean ± SEM, *n* = 3. Statistical significance was determined by a two-way ANOVA Test (**** *p* < 0.0001). The differences between the control nanodevices were not statistically significant and, therefore, were not indicated.

Encouraged by the promising 2D cell cancer culture results, we further evaluated the nanomotors performance in HeLa multicellular tumor spheroids-like cultures as a 3D model to better resemble *in vivo* behavior of tumors (Figure 3B). Once it was confirmed that NM_Doxo‑GOx_ nanomotors were able to deliver doxorubicin to deep layers of (Figure S25) tumor spheroids by CLSM, the cytotoxic activity was studied. Note that in this case it was impossible to evaluate nanoparticles’ behavior in the absence of glucose because glucose is essential for maintaining spheroid integrity. The viability assays showed that treating spheroids with NM_Doxo‑GOx_ (Figure 3Bii, red) significantly reduced cell viability (up to 48% at a concentration of 15 μg mL^–1^), whereas the treatment with the passive nanoparticles, NM_Doxo‑GOx‑IN_ (Figure 3Bii, black) and NM_Doxo‑BSA_ (Figure 3Bii, purple), induced lower toxicity at the same concentrations. We also confirmed that the control nanodevices, NM_GOx_ (lacking doxorubicin; Figure 3Bii, blue) and NA (unassembled nanomotor; Figure 3Bii, orange), did not lead to significant toxicity of tumor spheroids. The results demonstrated that the NM_Doxo‑GOx_ nanomotor exhibited a remarkable therapeutic effect triggering cell death in the tumor spheroid culture at a very low dose.

### Evaluation of Enhanced Penetration

In a further step, to investigate whether nanomotors’ autonomous movement could provide superior penetration we followed two different approaches. On the one hand, we evaluated the penetration of nanomotors in a model resembling the ECM (Figure 4A); and, on the other hand, we evaluated the penetration and drug delivery capacity of the nanodevice in tumor spheroids (Figures 4B and S26).

**Figure 4:**
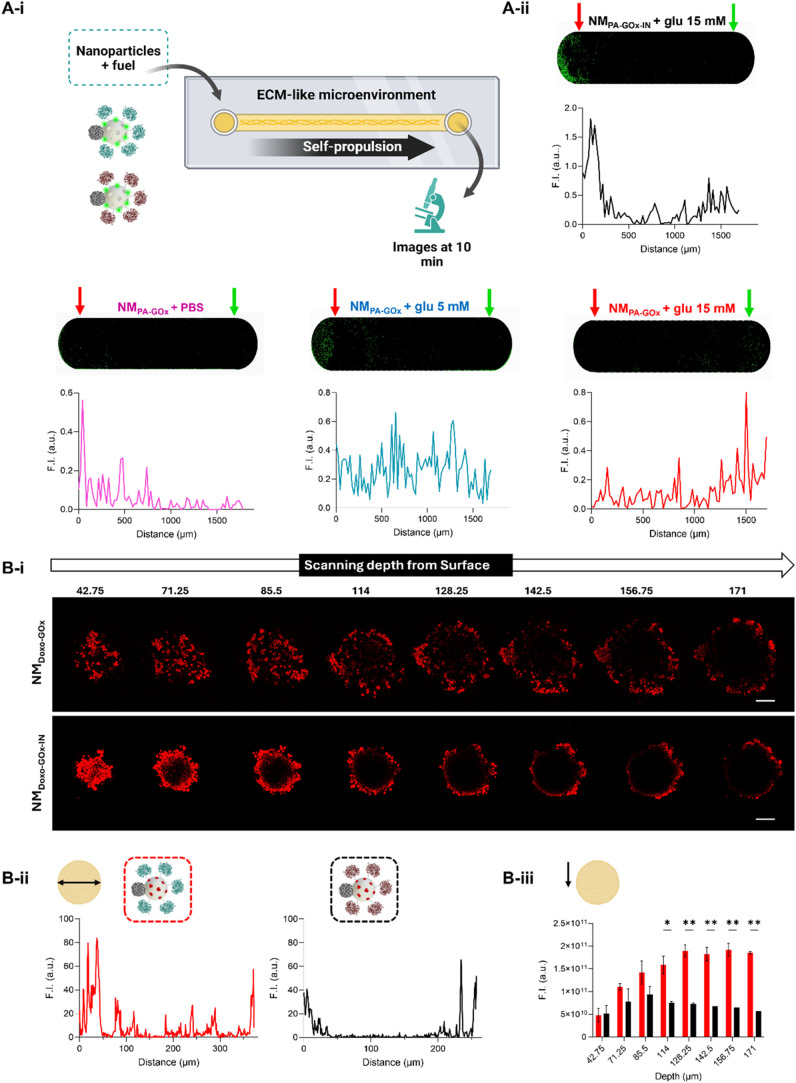
Penetration capabilities of the nanomotors. (A) Evaluation of nanomotors in channels filled with Matrigel. (A-i) Schematic of the design of the experiment. Created with Biorender.com. (A-ii) CLSM image showing the location of the nanodevices (labeled in green) and quantification of the fluorescence intensity along the channel using ImageJ software. NM_PA‑GOx_ + PBS: pink, NM_PA‑GOx‑IN_ + 15 mM glucose: black, NM_PA‑GOx_ + 5 mM glucose: blue, NM_PA‑GOx_ + 15 mM glucose: red. The red arrow indicates the point where the nanoparticles were added, and the green arrow indicates the opposite point at the end of the channel. (Scale bar = 1 mm). (B) Evaluation of the penetration into 3D tumor spheroids of HeLa cells of the nanomotor NM_Doxo‑GOx_ and the control NM_Doxo‑GOx‑IN_. (B-i) CLSM images ordered from shallowest to deepest (scale bar: 100 μm). (B-ii) Fluorescence intensity profile in one of the sections (depth: 114 μm). (B-iii) Quantification of fluorescence intensity in representative sections. The arrow indicates the direction of the planes. The determinations in graphs (B-ii) and (B-iii) were carried out with the program ImageJ (NM_Doxo‑GOx_: red, NM_Doxo‑GOx‑IN_: black), (*n* = 2). Statistical significance was assessed using a two-way ANOVA Test (* *p* < 0.05, ** *p* < 0.01).

First, we set up a motion assay on a two-compartment microfluidic platform aiming to mimic the viscosity and composition of the ECM in tumors.^69^ The channel of the microfluidic device (17 mm of length) was filled with Matrigel, which is a basement membrane matrix extracted from mice tumors. Matrigel composition includes approximately 60% laminin, 30% collagen IV, and 8% entactin.^70^ In addition, nanomotors were covalently labeled with a H_2_O_2_-resistant fluorophore (PA, see Figure S1 and Experimental Section) for visualization purposes (NM_PA‑GOx_ and NM_PA‑GOx‑IN_). Nanoparticles were placed at the beginning of the microfluidic channel, followed by the addition of glucose (5 or 15 mM) or PBS. After 10 min, PA fluorescence (green signal) was monitored along the channel by CLSM (see scheme in Figure 4Ai). Results, depicted in Figure 4Aii, indicate that NM_PA‑GOx_ (blue and red) showed a longer reach in the channel than the passive control NM_PA‑GOx‑IN_ in the presence of fuel (black) and NM_PA‑GOx_ in the absence of glucose (pink), which are mainly distributed close to the well where they were added (indicated by a red arrow). In addition, there were also differences between propelling NM_PA‑GOx_ with different amounts of biofuel. When 5 mM glucose (blue) was added, the PA fluorescent signal was distributed homogeneously through the channel. On the other hand, when 15 mM glucose (red) was added, the intensity was mostly located at the end of the channel (indicated by a green arrow). These findings indicate that glucose-triggered diffusion allows enhanced penetration of the nanomotor in an ECM-like microenvironment.

To further verify the enhanced deeper penetration of the nanomotors, we treated spheroids (approximate size: 300–400 μm) with NM_GOx_ (not containing doxorubicin to avoid doxorubicin-associated toxicity) and J-Pt (without GOx, as passive control) and quantified the number of nanoparticles in sections of the spheroids after 4 h by TEM (Figure S26). In both cases, nanodevices were clearly visualized inside the cell cytoplasm of tumor cells. Nevertheless, the number of NM_GOx_ nanoparticles per cell was almost 2-fold higher than that of J-Pt (as determined by ImageJ quantification and the statistical analysis), indicating that glucose-induced active motion results in increased cell uptake in 3D tumor spheroids.

Moreover, penetration of nanomotors was also studied by CLSM (Figure 4B). For this purpose, spheroids were treated with NM_Doxo‑GOx_ or NM_Doxo‑GOx‑IN_ (as control) for 24 h. After the application of both treatments, the presence of doxorubicin (red signal) was determined in the different sections of the spheroids. In the spheroids treated with NM_Doxo‑GOx‑IN_ (containing inactivated GOx, black), the fluorescent signal was mostly localized at the outer edge, while in the core and deeper layers no fluorescent signal was observed. On the contrary, in the spheroid treated with the active nanoparticle NM_Doxo‑GOx_ (red), the fluorescent signal was also localized in the central part of deeper layers. Fluorescence profiles of one of the sections (Figure 4Bii) and quantification of the total fluorescence intensity in the different sections ordered from shallowest to deepest (Figure 4Biii) indicated that NM_Doxo‑GOx_ is able to deliver greater amount of doxorubicin to a greater deeper compared with NM_Doxo‑GOx‑IN._ At this point it is important to note that, due to the high opacity and compactness of the tumor structure, laser penetration was limited to about the upper third of the spheroids (approximately 170 μm). The images also show that the integrity of the spheroids treated with the active NM_Doxo‑GOx_ nanomotor was affected because of their toxicity, which is a consequence of the synergistic combination of glucose-induced higher penetration, controlled drug delivery, and ROS production (H_2_O_2_ generation by GOx). In contrast, spheroids treated with the NM_Doxo‑GOx‑IN_ remained unaltered (which agreed with the cytotoxicity results, Figure 3).

Altogether, the *in vitro* studies demonstrated: (i) enhanced nanomotor movement powered by glucose, which resulted in enhanced cell uptake and deeper penetration in tumor spheroids and ECM-like matrices; and (ii) controlled doxorubicin release triggered by proteases leads to cancer cell death at very low nanomotors concentrations.

### *In Vivo* Antitumoral Effect

After confirming the capabilities of the engineered nanomotors *in vitro*, we went a step further by evaluating their antitumor effect *in vivo* in two HeLa xenograft mice models involving (i) an early stage and (ii) an advanced stage of tumor development. In these experiments, we opted to administer the nanomotors locally into the tumor. This is a useful approach and in fact some innovative onco-therapies already in clinical use are administrated intratumorally. Examples include local hyperthermia, cryotherapy, photodynamic therapy, brachytherapy, or local immunotherapy. Attending nanomaterials for cancer, regulatory agencies have already approved the use of magnetic nanoparticles injected into the tumor capable of inducing hyperthermia in the treatment of glioblastoma.^71^ In general, local deliver nanotechnologies provide important advantages over systemic ones, such as improved local drug retention, reduced systemic toxicity, and reduced administration frequency. Local administration is also a suitable alternative for the treatment of inoperable or recurrent tumors, as it can reduce cancer burden rendering tumors extirpable. In addition, since it does not affect other tissues, it could be used for palliative therapy.^72^

First, we studied the capability of the nanomotor to inhibit tumor growth at an early stage of the disease. For this purpose, the therapeutic effect of NM_Doxo‑GOx_ and two control nanoparticles, i.e NM_GOx_ (not loaded with doxorubicin) and NM_Doxo‑BSA_ (without self-propulsion ability) was studied. To generate the tumor-bearing model, HeLa cells were implanted (embedded in Matrigel via subcutaneous injection) in the flanks of Balb/c nude female mice. The administration of the nanotherapies started when tumor mass was visualized and localized to a small area (volume ca. 25 mm^3^). Nanoparticles were administered at a dosage of 15 μg mouse^–1^ (using PBS as vehicle) twice weekly for 4 weeks (see Experimental Section and Figure 5A). As illustrated in Figures 5B and S27A, tumors treated with NM_GOx_ (blue) and NM_Doxo‑BSA_ (purple) grew to levels comparable to those of the mice injected with the vehicle (gray). No therapeutic effect was revealed for incomplete nanoparticles at the applied concentrations. In contrast, the NM_Doxo‑GOx_ treated group showed significant tumor growth inhibition. This was also supported by the digital photo of the resected tumors at the end of the experiment (Figure 5C).

**Figure 5:**
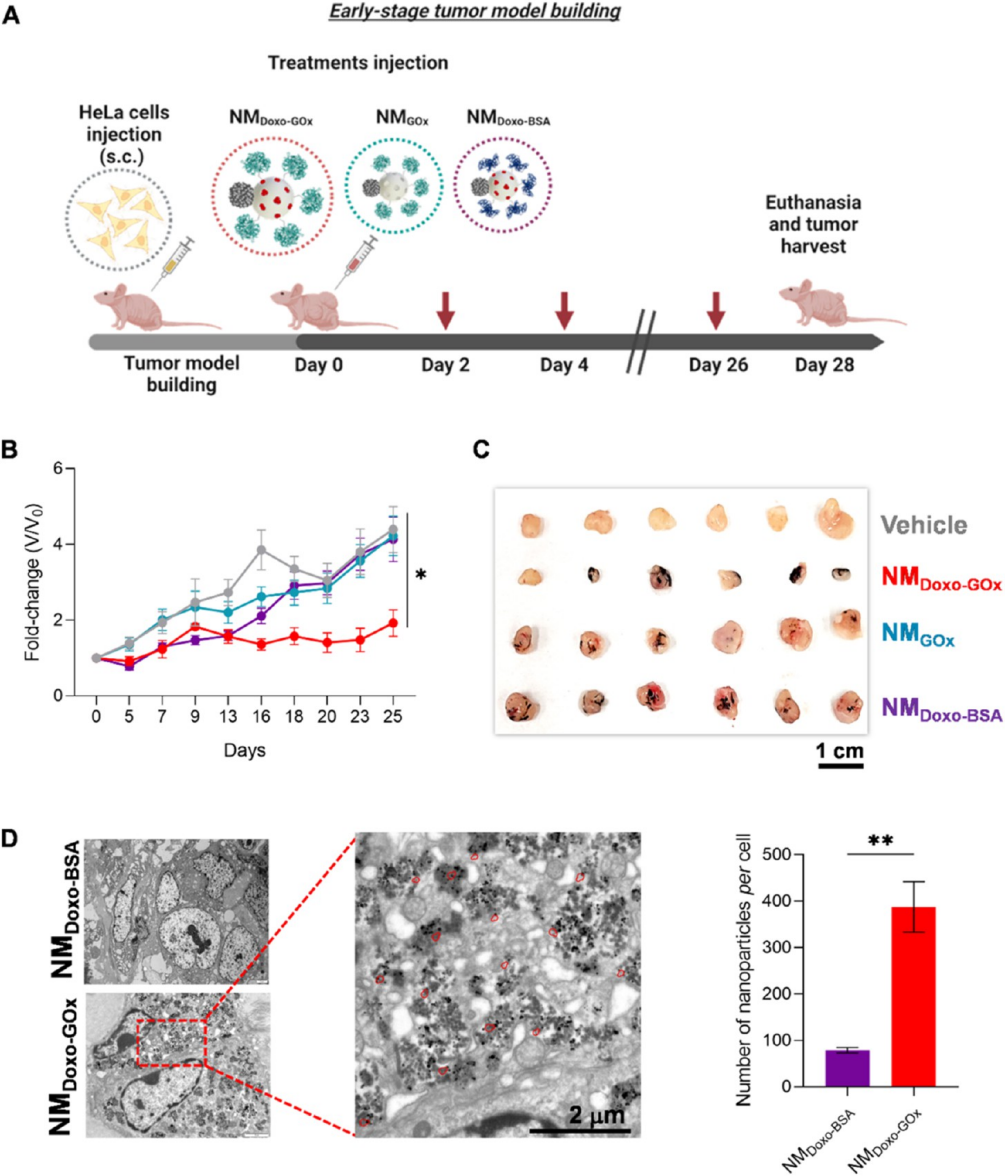
*In vivo* evaluation of the antitumor activity in an early stage cancer model. (A) Graphic description of nanomotors’ therapy in tumor-bearing Balb/c mice. Created with Biorender.com. (B) Monitorization of tumor growth upon treatment with nanomotors and the control nanoparticles. For each tumor, the relative volume change was determined and compared to the baseline before treatment (NM_Doxo‑GOx_, red; NM_GOx_, blue; NM_Doxo‑BSA_, purple; vehicle, gray). Data are expressed as mean ± SEM, *n* = 6. (C) Photograph of the excised tumors after applying different treatments. (D) Enhanced penetration of nanomotors in the tumors analyzed by TEM (*n* = 4). The NM_Doxo‑GOx_ image shows an increase in the number of nanoparticles in the cell cytoplasm (some marked in red) compared to the passive nanoparticles as quantified in the graph. Data are expressed as mean ± SEM. Statistical significance was determined by a one-way ANOVA, followed by Tukey’s test (* *p* < 0.05, ** *p* < 0.01).

One of the main objectives in the development of nanomotors is their potential ability to penetrate even hard-to-reach sites such as deep areas of tumors. For this reason, we further investigated the presence of nanoparticles in deep tumor tissue and the uptake of nanoparticles *in vivo*. Accordingly, we compared the number of nanoparticles per cell on tumor slides treated with either NM_Doxo‑GOx_ or NM_Doxo‑BSA_ by TEM. As shown in Figure 5D, a 4-fold increase in the number of NM_Doxo‑GOx_ nanoparticles was observed compared to the control. Representative images of the procedure followed for quantification of the number of nanoparticles *per* cell in spheroids and tumors are shown in Figure S28.

In addition, we studied by confocal fluorescence, whether NM_Doxo‑GOx_ would indeed induce enhanced drug delivery compared to the nanoparticles not endowed with movement *in vivo*. As shown in Figures 6Ai–iii and S29, CLSM images of the tumor slides of the animals treated with NM_Doxo‑GOx_ displayed a higher red fluorescent signal (derived from the released doxorubicin) than the tumor slides of the animals treated with the NM_Doxo‑BSA_ passive control. This confirmed that NM_Doxo‑GOx_ led to enhanced release of the loaded drug in tumors. Subsequent quantification of doxorubicin-related fluorescence intensity supported this conclusion (Figure 6Aiv). Furthermore, the histological analysis of apoptosis on tumor slides by the TUNEL assay revealed that NM_Doxo‑GOx_ treatment resulted in an increase in death cells compared to other controls (i.e., NM_GOx_ and NM_Doxo‑BSA_), as clearly observed by the resulting increase in nuclear green fluorescence (Figure 6B). ROS production in tumor-bearing Balb/c mice by using DCFDA-H2DCFDA as detection probe was also confirmed for tumors treated with NM_Doxo‑GOx_ compared to vehicle. This effect was not observed in tumors treated with controls (Figure S30).

**Figure 6:**
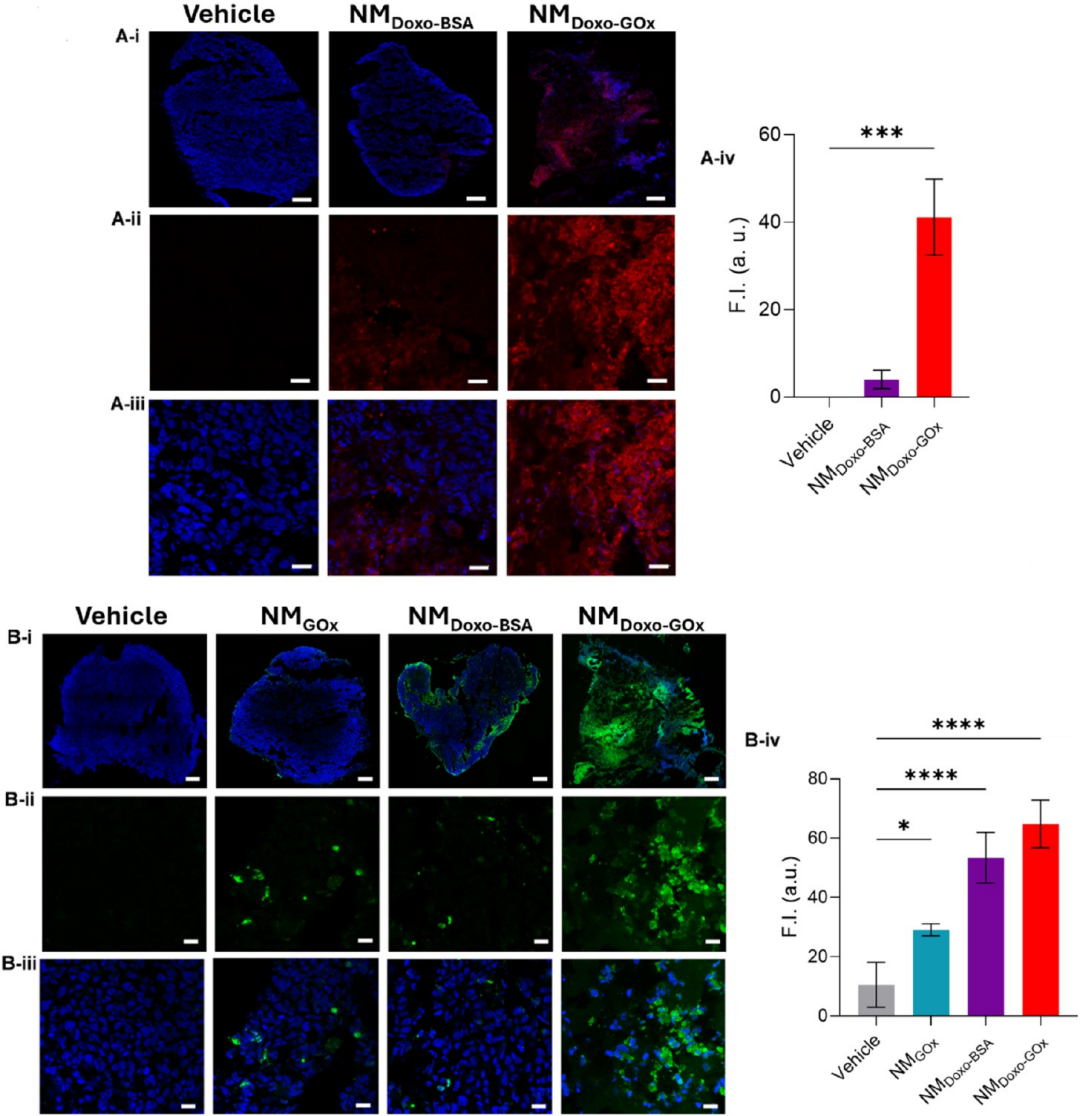
*In vivo* evaluation of the antitumor activity in an early stage cancer model: controlled drug delivery and cell death. (A-i) Representative CLSM photographs of doxorubicin delivery in tumor sections of the animals treated with NM_Doxo‑GOx_ and NM_Doxo‑BSA_ (cell nuclei, blue; doxorubicin, red; scale bar 100 μm). (A-ii) and (A-iii) Zoomed photographs (cell nuclei, blue; doxorubicin, red; scale bar 20 μm). (A-iv) Fluorescence intensity quantification (data represent mean ± SEM, *n* = 5) after selecting three random fields in each image. Statistical significance was determined by a two-way ANOVA, followed by Tukey’s test (** *p* < 0.01). (B-i) Representative CLSM images of the TUNEL assay showing apoptosis in the tumors of the mice treated with NM_Doxo‑GOx_, NM_Gox_, and NM_Doxo‑BSA_ (cell nuclei, blue; TUNEL^+^ positive cells, green; scale bar: 100 μm). (B-ii) and (B-iii) Zoomed images (cell nuclei, blue; TUNEL^+^ positive cells, green; scale bar 20 μm). (B-iv) Quantification of the corresponding TUNEL^+^-positive cells in 3 aleatory areas of each tissue section (data expressed as mean ± SEM, *n* = 4). Only the green signal from the cell nuclei was considered for quantification. Statistical significance was determined by a two-way ANOVA, followed by Tukey’s test (* *p* < 0.05, **** *p* < 0.0001).

Besides, the biodistribution of NM_Doxo‑GOx_ in mice was analyzed by determining the Pt levels in the tumor and major organs by ICP-MS (Figure S31). Pt was mainly found in the tumor, where NM_Doxo‑GOx_ was injected. Much lower Pt levels were also detected in the spleen and liver.^73–75^ These results suggest that the nanoparticles could be removed from tumors by the reticuloendothelial system and directed to the spleen where immune cells reside. In addition, the presence of Pt in liver might suggest that the nanoparticles are cleared from the body by hepatobiliary excretion. In addition, the biosafety of the treatment was assessed by hematoxylin-eosin and TUNEL staining of major organs of mice. The results showed no apparent pathological alterations (Figure S32) or apoptotic cells (Figure S33) in the organs of mice treated with NM_Doxo‑GOx_ compared to the organs of control mice. We also monitored the mice body weight throughout the experiment and analyzed blood samples at the end point to evaluate inflammation levels and liver and kidney functions. The results showed nonsignificant fluctuations in all the parameters measured (Figure S34). Altogether, these findings suggest that NM_Doxo‑GOx_ had no adverse side effects on the organism at the dosage regimen used.

Encouraged by all these promising findings, we moved one step forward to investigate whether the nanomotors were also capable of inducing a therapeutic effect even at a more advanced stage of the disease. In this case, the tumor-bearing model was generated as described above, however, the administration of the treatments began once the tumor volume reached ca. 70 mm^3^ (Figure S35, black). Tumors were treated with NM_Doxo‑GOx_ and, in this case, with the control nanoparticles NM_Doxo‑GOx‑IN_ (containing inactivated GOx). Nanodevices were administered via intratumoral injection (dosage of 15 μg mouse^–1^ dissolved in PBS) three times per week (see Experimental Section and Figure 7A).

**Figure 7:**
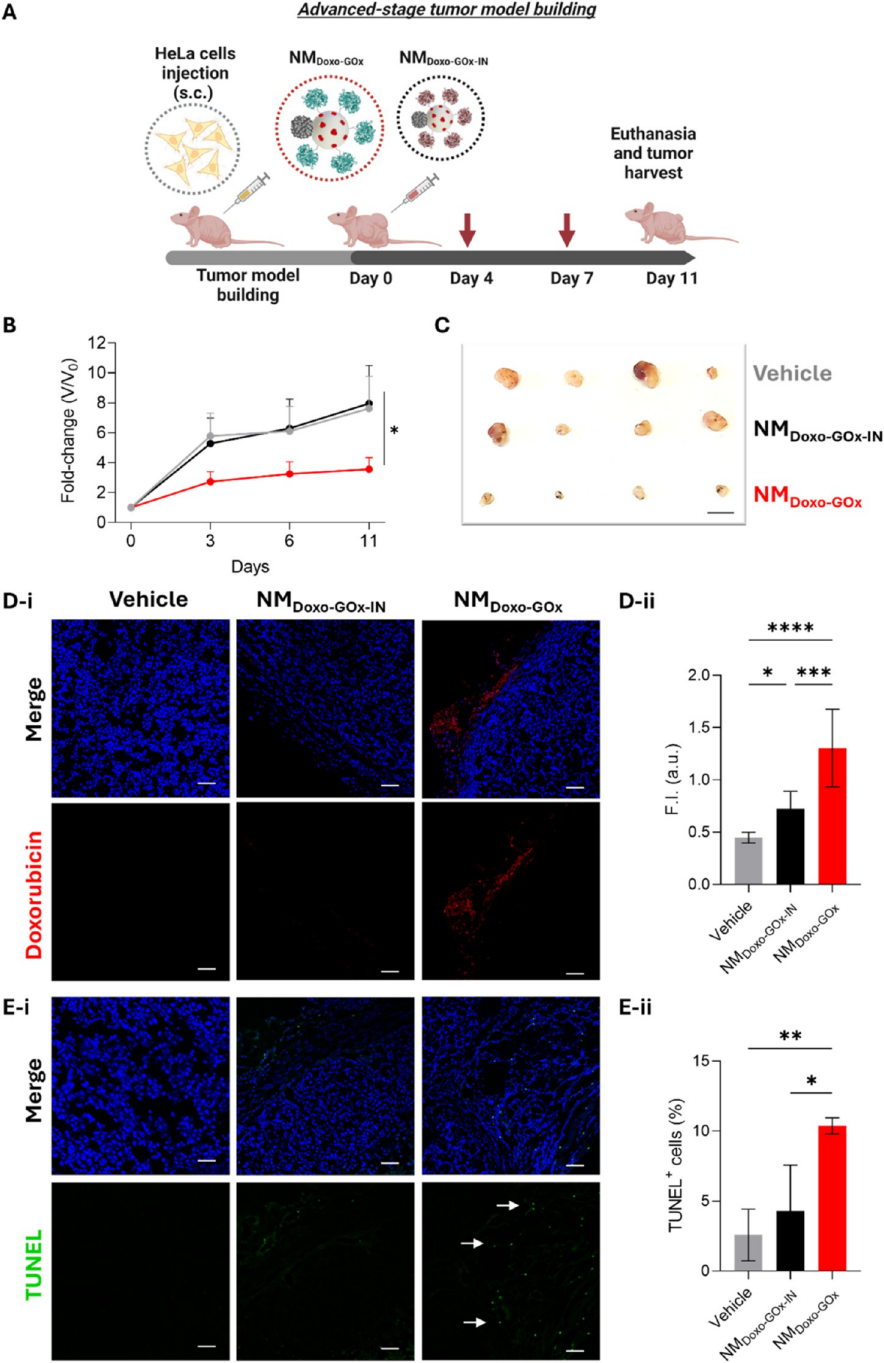
*In vivo* evaluation of the antitumor activity in an advanced-stage cancer model: tumor volume reduction, controlled drug delivery and cell death. (A) Schematic of nanomotors’ therapy timeline in tumor-bearing Balb/c mice. Created with Biorender.com. (B) Fold-change of tumor volume after the different treatments (NM_Doxo‑GOx_, red; NM_Doxo‑GOx_, black; and vehicle, gray). Data are expressed as mean ± SEM, *n* = 9. (C) Photograph of representative excised tumors at the end of the experiment (scale bar = 1 cm). (D-i) Representative CLSM photographs of doxorubicin delivery in tumor sections of the animals (cell nuclei, blue; doxorubicin, red; scale bar 50 μm). (D-ii) Fluorescence intensity quantification in 6 random areas of each tumor section (data represent mean ± SEM, *n* = 4). (E-i) Representative CLSM images of the TUNEL assay showing apoptosis in the tumors of the mice (cell nuclei, blue; TUNEL^+^-positive cells, green and marked with an arrow; scale bar: 50 μm). (E-ii) Quantification of the corresponding TUNEL^+^-positive cells in 6 random areas of each tumor section (data expressed as mean ± SEM, *n* = 4). Statistical significance was determined by a two-way ANOVA, followed by Tukey’s test (* *p* < 0.05, ** *p* < 0.01, *** *p* < 0.001, **** *p* < 0.0001).

As shown in Figure 7B–C and S27B, NM_Doxo‑GOx_ treatment resulted in significant reduction of tumor growth (red). On the contrary, treatment with NM_Doxo‑GOx‑IN_ was not effective (black), as the tumors grew like those treated with vehicle (gray). Treatments could not continue longer than day 11 because exponential tumor growth that affected mice well-being. In contrast, the well-being of mice treated with the complete NM_Doxo‑GOx_ was not compromised. Tumor volumes at the end of the experiment and mice body weight variation are shown in Figures S36 and S37, respectively. Treatment with NM_Doxo‑GOx_ resulted in larger doxorubicin delivery (red fluorescence, Figure 7D) and population of TUNEL^+^ cells (green fluorescence, Figure 7E) compared to controls. Subsequent quantification of doxorubicin and TUNEL fluorescence intensity in different tumor sections supported these conclusions (Figure 7Dii,Eii). We also studied the distribution of NM_Doxo‑GOx_ in the tumor in this advanced-stage cancer model. In this experiment a complete tumor treated with NM_Doxo‑GOx_ was divided into four representative areas and evaluated by TEM. Nanoparticles were found in all tumor sections, indicating that NM_Doxo‑GOx_ are able to penetrate and distribute throughout the entire tumor tissue (Figure S38). Furthermore, tumors from NM_Doxo‑GOx_-treated mice showed tissue degradation with a significant decrease in cell number and a reduction in hypoxia levels, as observed by quantifying the number of cell nuclei (Figure S39) and hypoxic adducts (Figure S40) in tumor sections, respectively.

Altogether, the above *in vivo* results evidence that NM_Doxo‑GOx_ presents a remarkable therapeutic effect on reducing tumor volume both at an early- and an advance-stage of the disease. This antitumor performance results from the synergy between the controlled drug delivery (by proteases) and the catalytic activities of GOx and PtNds that overall induce enhanced motion, consumption of glucose (starvation therapy), ROS production (contributing to oxidative stress)^65–68^ and hypoxia alleviation (by O_2_ generation).^31^ These features suggest that engineered nanoparticles, such as NM_Doxo‑GOx_, have interesting improved potential in drug therapy.

### Evaluation in Patient-Derived Organoids

In the attempt to better assess the therapeutic effectiveness of the designed nanomotors within a context more similar to real clinical scenarios, NM_Doxo‑GOx_ efficacy was evaluated using breast cancer patient-derived organoids (PDOs) (Figure 8). PDOs allow to predict patient’s response better than cancer cell lines as they retain the features of the tumor of origin. We employed PDOs originated from a tumor sample from a metastatic triple-negative breast cancer patient, which represents an aggressive disease associated with poor patient’ outcomes. TNBC serves as an ideal model to validate this novel strategy considering its fibrotic nature and an immunosuppressive tumor microenvironment (TME) influenced by cancer-associated fibroblasts (CAFs). These cells modify TME by metabolic reprogramming, extracellular matrix production and remodeling, resulting in drug resistance and immunosuppression that hinder effective therapy.^76,77^

**Figure 8:**
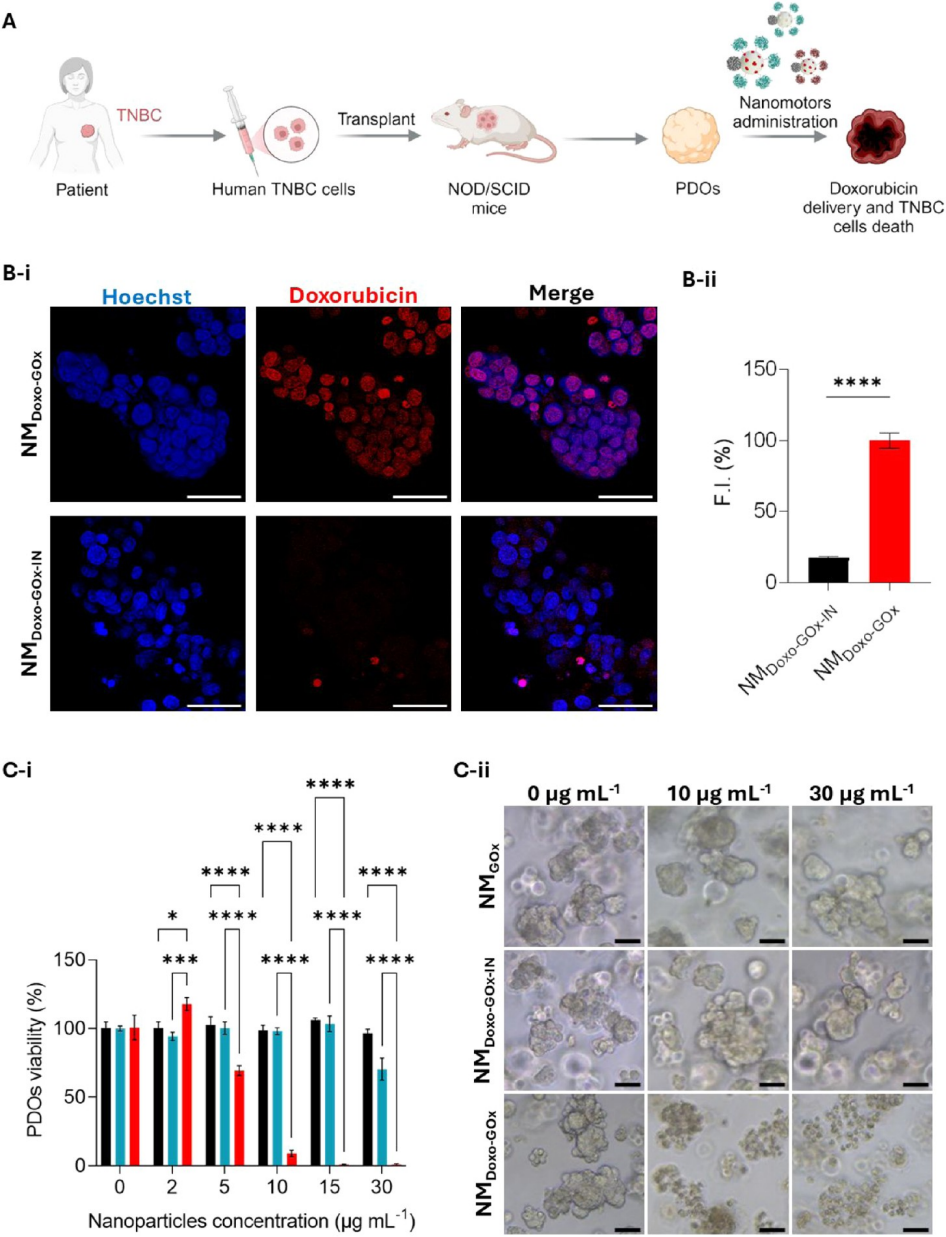
Evaluation of the antitumor effect in breast PDO. (A) Schematic of PDO formation and therapy. Created with Biorender.com. (B) Doxorubicin delivery into PDOs. PDOs were treated with 50 μg mL^–1^ of NM_Doxo‑GOx_ (red) or NM_Doxo‑GOx‑IN_ (black) for 2 h and accumulation of doxorubicin was evaluated by CLSM. (B-i) Representative images are presented (blue: nuclei; red: doxorubicin; scale bar: 50 μm). (B-ii) Mean fluorescence intensity of doxorubicin was determined (mean ± SEM). (C) PDOs viability. PDOs were incubated for 24 h with different concentrations of NM_Doxo‑GOx_ (red), NM_GOx_ (blue) or NM_Doxo‑GOx‑IN_ (black) and viability was determined. (C-i) Data are presented as mean ± SEM. (C-ii) Representative optical microscopy images of PDOs are shown, (scale bar: 300 μm).

First, PDOs were treated with either NM_Doxo‑GOx_ or the passive control NM_Doxo‑GOx‑IN_ for 2 h and then evaluated by CLSM to determine their ability to deliver doxorubicin (Figure 8B). The results revealed a robust doxorubicin fluorescent signal localized within the cell nuclei upon the treatment with NM_Doxo‑GOx_, whereas treatment with the inactive control yielded a minimal signal (approximately 20%), underscoring the importance of the enzymatic activity to enhance doxorubicin penetration into PDOs. Then, the cytotoxic activity was further evaluated (Figure 8C). The results demonstrate that treatment of PDOs with NM_Doxo‑GOx_ induced a decrease in cell viability even at low concentrations (approximately 90% at 15 μg mL^–1^, Figure 8Ci). The strong cytotoxic effect of the nanomotor was also corroborated in optical microscopy images of PDOs, in which the loss of their three-dimensional structure and the presence death cells can be observed (Figure 8Cii). Additionally, PDOs were also treated with inactive control (NM_Doxo‑GOx‑IN_), which exhibited no toxic effect at the tested concentrations. Treatment with NM_GOx_ (unloaded nanoparticle) only induced a mild toxic effect (approximately 70% of viable cells) at the highest concentration tested, most likely attributed to the generation of ROS by the GOx enzyme.

## Conclusions

To summarize, we report on the successful construction, characterization, and validation of multifunctional gated nanomotors to treat solid tumors. The developed system is based on the conjunction of PtNds and MSNs in single anisotropic Janus snowman-type nanoparticles. The MSNs’ face is loaded with anticancer drug doxorubicin and capped with GOx (NM_Doxo‑GOx_). The enzyme constitutes both the gatekeeper (by modulating cargo release in response to cellular proteases) and the first element of the propulsion system (by transforming endogenous glucose from the tumoral microenvironment into H_2_O_2_). In turn, the PtNds’ face acts as the second catalytic element in a hybrid cascade reaction. PtNds transform the locally produced H_2_O_2_ into O_2_ and H_2_O, finally driving nanomotors by products accumulating on one face, probably following a self-diffusiophoresis mechanism.^62–64^ As demonstrated by the different biological experiments, the engineered nanomotors exhibited great antitumor capability *in vitro*, *in vivo* and PDO models. Overall, we propose that this is due to a unique nanoarchitectonics design that combines a controlled drug delivery mechanism and the catalytic activities of GOx and PtNds. This induces not only enhanced motion, but also consumption of glucose, ROS production and hypoxia alleviation (O_2_ generation). All of these together lead to increased cancer cell death in different models. Our approach demonstrates the application of the designed nanoparticles *in vivo* as efficient antitumoral agents in both early and advanced stages of the disease. In addition, the efficacy of the nanoparticles has been also demonstrated in a patient-derived organoid model, which is more similar to the real clinical scenario. In this context, there is a relation between the dose of nanoparticles administered and the observed therapeutic and nontherapeutic responses. We have been able to maximize the local efficacy of the treatment while minimizing the systemic effects associated with conventional therapies by administrating a very low dose of nanoparticles specifically in the tumor microenvironment. Therefore, our approach optimizes drug delivery strategies in tumors, improving therapeutic selectivity by allowing controlled and targeted drug release. The remarkable capabilities of drug delivery in tumors provides a promising nanoplatform for cancer therapy. Thanks to their versatility, our nanoarchitectonic design has a broad scope. J-Pt nanoparticles can be loaded with other drugs and functionalized with other biomolecules to potentially penetrate other biological barriers of interest, such as microbial biofilms, skin, or the blood-brain barrier. We believe that our results provide a significant advance toward clinical applications and may pave the way to produce tailor-made smart nanocarriers for therapeutic purposes.

## Experimental Section

### Synthesis and Characterization of Nanoparticles

#### Synthesis of MSNs

*N*-cetyltrimethylammonium bromide (CTAB) was dissolved in 480 mL of deionized water at a concentration of 2.74 mM. To increase pH, 3.5 mL of NaOH 2 M were added, and temperature was adjusted to 80 °C. Then 5 mL of silicon precursor tetraethyl orthosilicate (TEOS) were added dropwise to the surfactant solution. The mixture reacted for 2 h under magnetic stirring. The resultant solid was isolated by centrifugation, washed with deionized water until a neutral pH was reached and dried at 70 °C. Finally, that solid was calcinated at 550 °C in an oxidant atmosphere for 5 h to obtain the template-free mesoporous silica nanoparticles-type MCM-41 (MSNs).

#### Synthesis of PtNds

164 mg of H_2_PtCl_6_ and 200 mg of poly­(vinylpyrrolidone) (PVP) were dissolved in 20 mL of deionized water. Then 10 mL of an aqueous solution of ascorbic acid (0.2 M) were added dropwise to the previous solution, and the mixture was heated at 55 °C for 3 h under magnetic stirring. The resultant black color solution reveals the presence of branched platinum nanoparticles, known as nanodendrites (PtNds).^40,42^

#### Synthesis of J-Pt

180 mg of MSNs were homogeneously dispersed in 10 mL of 1 μM CTAB in 6.7% ethanol solution. The mixture was heated at 75 °C. Then 1 g of paraffin wax was added. After melting paraffin, an Ultra-Turrax T-8 (IKA, Germany) was used to homogenize the mixture for 10 min at 25,000 rpm. The emulsion was magnetically stirred for 1 h at 75 °C to form a Pickering emulsion. Then it was cooled to room temperature before 200 μL of 3-(mercaptopropyl)­trimethoxysilane and 10 mL of methanol were added. After 3 h, the silanized solid was isolated by centrifugation and washed with methanol twice. Next the solid was dispersed in 68 mL of methanol and mixed with 30 mL of PtNds. The reaction was left under stirring conditions for 24 h at room temperature. Finally, the solid was filtered, washed with chloroform, and dried to yield Janus Pt-MSN nanoparticles (J-Pt).^34–37^

#### Synthesis of NM_Doxo‑GOx_

First, 25 mg of J-Pt were diluted in 3 mL of anhydrous acetonitrile (ACN) and 25 μL of (3-aminopropyl)­triethoxysilane (APTES) were added to the mixture. The reaction was left under stirring conditions for 5.5 h in an inert atmosphere to partially functionalize the mesoporous side with amine groups. Later the solid was washed several times with ACN, ethanol and 100 mM PBS pH 7.5. For the loading process, 12.5 mg of doxorubicin were diluted with 3 mL of PBS and added to the solid. The reaction took place in the dark under magnetic stirring for 24 h. Then the solid was centrifuged again. To end gatekeeper construction, the acidic groups of GOx were activated. For this purpose, 6.25 mg of GOx were solved in 3 mL of 100 mM PBS, pH 6, and 6.25 mg of *N*-(3-(dimethylamino)­propyl)-*N*′-ethylcarbodiimide hydrochloride (EDC). Then 6.25 mg of *N*-hydroxysuccinimide (NHS) were added. The reaction was left for 30 min at 4 °C and stirring. This was followed by washing the enzyme several times with 100 mM PBS, pH 6. Then 25 mg of the amino-functionalized solid were combined with the activated GOx. The reaction took place in 100 mM PBS, pH 6, under magnetic stirring at 4 °C overnight. Finally, the solid was isolated by centrifugation and washed with PBS to yield the final complete nanomotor Janus Pt-MSN-(Doxo)-GOx (NM_Doxo‑GOx_).

#### Synthesis of NM_GOx_

These control nanomotors (NM_GOx_) were capped with GOx. They were not loaded but left empty. The procedure followed for their synthesis was similar to that of NM_Doxo‑GOx_, but the drug-loading step was omitted.

#### Synthesis of NM_Doxo‑BSA_

These control nanoparticles (NM_Doxo‑BSA_) were loaded with doxorubicin, but the employed gatekeeper was bovine serum albumin (BSA) protein with no catalytic activity. The protocol followed to prepare them was like that of NM_Doxo‑GOx_, but the protein was changed from GOx to BSA in the gatekeeping assembly step.

#### Synthesis of NM_Doxo‑GOx‑IN_

These control nanoparticles without movement (NM_Doxo‑GOx‑IN_) were prepared by inactivating the catalytic activity of GOx by heat. For this purpose, the final nanomotors NM_Doxo‑GOx_ were incubated at 70 °C for 1 h.

#### Synthesis of NM_PA‑GOx_ and NM_PA‑GOx‑IN_

These nanomotors were modified with a H_2_O_2_-resistant fluorophore to enable visualization by CLSM. First, the synthesis of the fluorophore, which consisted of an APTES-modified 3,4,9,10-perylenetetracarboxylic dianhydride diimide derivative (PTDA) (PA), was performed. In the first reaction step, 2.81 g of 6-aminohexanoic acid (21 mmol) was mixed with 4.04 g of PTDA (10 mmol) and 28 g of imidazole, and the mixture was heated at 120 °C for 1 h under argon atmosphere. The reaction mixture was cooled to 90 °C and diluted with distilled water. The resulting dark red solution was filtered and acidified with 2 M HCl to pH 3–4. The resulting solid was isolated by filtration and dried under vacuum, yielding the diimide PTDA. In a second reaction step, the solid was mixed with 85.57 μL of APTES (0.362 mmol), 8.12 mg of DMAP (0.066 mmol), 77.27 mg of EDC (0.392 mmol) and 55.61 μL of TEA (0.395 mmol) in 10 mL of dichloromethane. The reaction was magnetically stirred for 4 days at room temperature. After that time, 10 mL of chloroform was added, and washing was performed with 20 mL of NaHCO_3_ (5%) and 20 mL of distilled water. The organic phase was then dried with MgSO_4_, filtered and finally the solvent was removed on a rotary evaporator, giving the final orange colored compound (λ_exc_ = 492 nm, λ_em_ = 550 nm).^78,79^

To label the nanomotor surface with PA, 5 mg of NM_APTES_ were suspended in 2 mL of chloroform and 5 mg of the product (1 mmol) were added. The reaction was kept under magnetic stirring for 5.5 h. Then, the nanomotors were washed by centrifugation (13,500 rpm, 3 min) with chloroform, ethanol, and PBS (100 mM, pH 7.5) several times. Finally, the enzyme anchoring was performed under the same conditions described for NM_Doxo‑GOx_, yielding NM_PA‑GOx_. Nonmotile control nanoparticles, NM_PA‑GOx‑IN_, were also prepared. In that case, the same synthetic protocol was followed, but last the enzyme activity was inactivated by heat (70 °C, 1 h).

All the nanoparticles were stored in PBS at 4 °C until used.

#### Determination of the Gatekeeper Quantity

The BCA method was followed to calculate the quantity of the enzyme GOx attached to the mesoporous face of the nanoparticles. The technical procedure with several modifications was followed.^47^ Then 10 μL of nanoparticles (1 mg mL^–1^) were incubated with 200 μL of B/A solution (1:50) for 10 min at 60 °C. Later samples were centrifuged, and the absorbance of the supernatant was measured at 570 nm. The calibration line was prepared with different concentrations of free BSA (from 7.8 to 1000 μg mL^–1^) in PBS 100 mM at pH 7.5.

### Analysis of Controlled Delivery Capability of Nanomotors

#### On-Command Protease-Triggered Doxorubicin Delivery by Nanomotors

For the *in vitro* cargo-controlled release studies, 1.5 mg of NM_Doxo‑GOx_ were suspended in 2 mL of 10 mM PBS, pH 7.5, and separated into two fractions. Suspensions were placed inside a shaker at 37 °C and incubated for 30 min. Next 1 mg of protease from S. griseus were added to one of them. At successive times (5, 10, 15, 20, 30, 60, and 120 min), mixtures were centrifuged (5 min, 12,500 rpm) to eliminate nanomotors. Then the fluorescence emission of the doxorubicin released to media was measured (λ_exc_ = 470 nm, λ_em_ = 555 nm). Forced release assays were performed to determine the total amount of doxorubicin contained in the different nanoparticles. In each case, 0.5 mg of nanoparticles were suspended in 1 mL of DMSO and left to stir for 24 h in the dark. Suspensions were then centrifuged, and the absorbance of the supernatant was measured (λ_abs_ = 495 nm). The doxorubicin concentration was inferred by Lambert–Beer’s law (eq 1). The loading efficiency (DLE for the drug and LE for the enzyme) of the nanomotor was determined by eq 2.

1 A=ε·c·l

where *A* is absorbance, *c* is the molar concentration (M), *l* is the optical length path (cm) and ε is the molar absorption coefficient of doxorubicin (9.250 mM^–1^ cm^–1^).

2 $$\mathrm{DLE}=\frac{C_{loaded}}{C_{total}}\cdot 100//\mathrm{LE}\frac{C_{\mathrm{bound}}}{C_{\mathrm{total}}}\cdot 100$$

where DLE is the drug loading efficiency, *C*_loaded_ is the amount of drug encapsulated, *C*_total_ is the total amount of drug or enzyme added in the synthetic procedure, LE is the loading efficiency an *C*_bound_ is the amount of enzyme attached.

#### On-Command Doxorubicin Delivery Triggered by Lysosomal Extract

For the cargo-controlled release studies simulating the cellular endocytic microenvironment, 1 mg of NM_Doxo‑GOx_ was suspended in 1 mL of purified lysosomal extract. In addition, the same amount of the final nanomotor was suspended in 1 mL of 10 mM PBS, pH 7.5 as a control. The mixtures were placed on a shaker at 37 °C and centrifuged (5 min, 12,500 rpm) at successive times (5, 30, 60, 120, 180, 240, and 300 min) to remove the nanomotors. Finally, the fluorescence emission of the doxorubicin released into the medium was measured under the usual parameters. The lysosomal extract was obtained according to the manufacturer’s indications (Lysosome Isolation Kit, LYSISO1) from fresh rabbit liver and used in a 1:4 dilution.

#### Protease Threshold for On-Demand Delivery of Doxorubicin by Nanomotors

To study the minimum protease concentration that produces controlled release of cargo, 0.75 mg of NM_Doxo‑GOx_ was suspended in 1 mL of 10 mM PBS, pH 7.5 as a control. The same amount of NM_Doxo‑GOx_ was suspended in 1 mL of increasing concentrations (between 0.05 and 1 mg mL^–1^) of proteases from S. griseus. The samples were incubated in a shaker at 37 °C and at successive times (30, 60, and 120 min) were centrifuged to remove nanomotors (5 min, 12,500 rpm). Then the fluorescence emission of doxorubicin released to the medium was measured.

### Analysis of Glucose-Induced Motility of Nanomotors

#### Glucose Oxidase Enzymatic Activity Assay

To confirm that the immobilization of GOx to nanoparticles did not damage its ability to produce H_2_O_2_ from glucose, an enzymatic activity assay was performed. This test is based on UV–visible spectrophotometry and consists of the capability of H_2_O_2_ to oxidize 2,2′-azino-bis­(3-ethylbenzothiazoline-6-sulfonic) acid (ABTS) in a blue-green product (ABTS^2–^), which is followed by this technique (λ_abs_ = 405 nm) in the presence of PtNds.^80^ The chemical reactions of the GOx enzymatic activity assay are the following

Reactions 3 and 4

R1 $$D‐\mathrm{glu}\,\mathrm{cose}+O_{2}+H_{2}O\overset{\mathrm{glucose}\;\,\mathrm{oxid}\mathrm{ase}}{\to }D‐\mathrm{gluconic}\;\mathrm{acid}+H_{2}O_{2}$$

R2 $$H_{2}O_{2}+\mathrm{ABTS}\overset{\mathrm{PtNds}}{\to }\mathrm{ABT}S^{2-}+H_{2}O$$

In a typical experiment, solutions of glucose 1 M (180 mg mL^–1^) and ABTS (1 mg mL^–1^) were prepared in distilled water. Then 250 and 250 μL were respectively mixed in a quartz cuvette, to which 10 μL of PBS (blank) or 10 μL of the nanoparticle’s suspension (1 mg mL^–1^) were added. After shaking samples, the absorbance was recorded at 405 nm according to time (2 min at 25 °C). To calculate enzymatic activity, eq 5 was applied.

3 $$\frac{\mathrm{enzyme}\;\mathrm{units}}{g}=\frac{\left(D-D_{\mathrm{blank}}\right)\cdot V_{T}\cdot F_{D}}{\varepsilon_{\mathrm{ABTS}}\cdot l\cdot V_{S}\cdot C_{S}}$$

where Δ is the slope of the graph (min^–1^), Δ_blank_ is the slope of the graph for the blank (min^–1^), *V*_T_ is the total volume in the cuvette, *F*_D_ is the dilution factor, ε_ABTS_ is the molar extinction of ABTS at 405 nm (36.87 mM^–1^ cm^–1^), *L* is the optical path in the cuvette (1 cm), *V*_S_ is the volume of the added sample (mL) and *C*_S_ is the concentration of the added sample (g mL^–1^).

#### Peroxidase-like Activity Assay

To confirm that J-Pt (and therefore the other nanoparticles synthesized from it) was able to catalyze H_2_O_2_, a peroxidase-like activity test was run. Once again, this test was based on the UV–visible spectrophotometry detection of ABTS^2–^, which was produced in the presence of H_2_O_2_ and PtNds.^81^ In the experiment, 500 μL ABTS (1 mg mL^–1^), 500 μL H_2_O_2_ (0, 1, 2.5, 5, 15, and 30 mM) and 40 μL (1 mg mL^–1^) of J-Pt were mixed and the increment in absorbance (λ_abs_ = 405 nm) was recorded for 2 min at 25 °C. Enzymatic-like Michaelis–Menten parameter (*K*_M_), maximum velocity (*V*_max_), turnover rate (*K*_cat_) and catalytic efficiency were calculated by applying eqs 6–8 to the results represented in Figure S11B.

4 $$\frac{1}{v}=\frac{K_{M}}{V_{\max}}\cdot \frac{1}{\left[S\right]}+\frac{1}{V_{\max}}$$

where *v* is reaction velocity, *K*_M_ is the Michaelis–Menten constant, *V*_max_ is the maximum reaction velocity and *S* is the concentration of the substrate.

5 $$K_{\mathrm{cat}}=\frac{V_{\max}}{\left[E\right]}$$

where *K*_cat_ is the turnover rate, *V*_max_ is the maximum reaction velocity and [*E*] is the molar concentration of the nanomotor, calculated as previous studies.^82^

6 $$\mathrm{catalytic}\;\mathrm{efficiency}=\frac{K_{\mathrm{cat}}}{K_{M}}$$

where *K*_cat_ is the turnover rate and *K*_M_ is the Michaelis–Menten constant.

#### Measurement of Glucose Concentration

To evaluate the capability of NM_Doxo‑GOx_ to consume glucose, the nanomotor (i.e., NM_Doxo‑GOx_) and NM_Doxo‑GOx‑IN_ (both at a final concentration of 300 μg mL^–1^) were incubated in 2 mL of PBS (pH 7.5) containing 5 mM glucose for 30 min at 37 °C. Then, samples were centrifuged (12,500 rpm, 3 min) to remove the nanoparticles and aliquots of 250 μL were taken and mixed with 250 μL of phenol (5% in water) and 1 mL of H_2_SO_4_ and incubated for 10 min at 25 °C. Moreover, a glucose calibration curve was prepared (concentrations from 0 to 5 mM). The formation of a yellow-colored compound was determined by UV–visible spectrophotometry (λ_abs_ = 490 nm). Glucose concentration was determined by interpolation on the calibration curve.

#### Measurement of H_2_O_2_ Concentration

The reaction was set up under identical conditions to the prior assay, using the same concentrations of NM_Doxo‑GOx_ and NM_Doxo‑GOx‑IN_ (300 μg mL^–1^). The samples were also centrifuged (12,500 rpm, 3 min) to remove the nanoparticles and the supernatants were mixed with 100 μL of PBS and 20 μL of a “master mix” containing 200 μL of HRP (10 U mL^–1^) in PBS pH 7.5 and 10 μL of Ampliflu Red (10 mM) in DMSO and measured in a fluorimeter (λ_exc_ = 560 nm, λ_em_ = 590 nm). To quantify the concentration of H_2_O_2_, a calibration curve was employed (concentrations from 0 to 3 mM).

#### Measurement of O_2_ Generation

Fifteen μL of NM_Doxo‑GOx_ (final concentration of 30 μg mL^–1^) were mixed with different concentrations of glucose (0, 2.5, 5, 10, and 15 mM) in a total volume of 5 mL of zero oxygen solution (hypoxic conditions). Measurements were taken using an oximeter every 5 min for 60 min.

#### MSD Calculation

We employed the device Nanosight NS300 and nanoparticle tracking analysis (NTA) by software 3.0 to characterize the motion behavior of single nanomotors and the control nanoparticles were prepared. We evaluated different increasing glucose concentrations (0, 2.5, 5, 10, 15, and 25 mM), which were first placed in the Nanosight chamber to avoid a possible drift associated with fuel introduction. Next nanodevices (NM_Doxo‑GOx_, NM_Doxo‑GOx‑IN_ and NM_Doxo‑BSA_) were added at a final concentration of 2 μg mL^–1^ in PBS, pH 7.5, after ultrasonication (final total volume of 800 μL, temperature of 25 °C). Then the instrument (via an optical microscope and an sCMOS camera) recorded 30 s videos at a speed of 30 frames s^–1^. This allowed the coordinates (*x*, *y*) of 50 nanoparticles to be extracted over time (Δ*t* = 1 s). Note that nanomotors smaller than 250 nm or larger than 350 nm were ruled out to avoid analyzing residues or aggregates, respectively. Based on this information, an in-house R code calculated the mean square displacement (MSD) for each nanoparticle (eq 9) by assuming that motion was two-dimensional. Finally, the program allowed us to obtain either the *D*_eff_ of nanomotors or the *D*_0_ for the control nanoparticles and nonfueled nanomotors, both by plotting MSD vs Δ*t* and by applying eq 10. By following the same software, the spatial trajectories of nanodevices were represented.

7 $$\mathrm{MSD}=\left\{{\left(x_{t}-x_{0}\right)}^{2}\right\}=\frac{1}{N}\sum_{i=0}^{N}(x^{i}\left(t\right)-x^{t}{\left(0\right))}^{2}$$

where MSD is mean square displacement, *N* is number of nanoparticles, and *x* ^*i*^ and *x* ^*t*^ are particles’ vector positions at different times.

8 $$\mathrm{MSD}=\left(4D_{\mathrm{eff}}\right)\cdot \Delta t//\mathrm{MSD}=\left(4D_{0}\right)\cdot \Delta t)$$

where MSD is mean square displacement, *D*_eff_ is the effective diffusive coefficient for the active nanoparticles, *D*_0_ is the diffusion coefficient for the nonpropelled nanoparticles and Δ*t* is the time increment.

#### Motion in an ECM-like Microenvironment

To demonstrate the ability of nanomotors to self-propel in a complex medium that simulates the ECM microenvironment, a CLSM analysis in a microfluidic device coated with Matrigel was performed. For this purpose, the microslide (μ-Slide VI 0.5 Glass Bottom, purchased from Ibidi) was filled with Matrigel. Later nanodevices NM_PA‑GOx_ or NM_PA‑GOx‑IN_ (0.25 mg mL^–1^) and the fuel glucose (5 or 15 mM) were added at one end of the channel. After 10 min, CLSM images (10×) of the channel were obtained. Image analysis was performed with ImageJ software.

### Evaluation of Nanomotors’ Anticancer Capabilities

#### Cancer Cell Culture

HeLa (a human-derived cervical cancer cell line), 4T1 (a mice-derived triple negative breast cancer cell line) and SK-Mel-103 (a human-derived melanoma cancer cell line) cells were used. Cells were cultured in DMEM high glucose medium supplemented with 10% FBS and incubated in a 5% CO_2_ humidified atmosphere at 37 °C.

#### Nanomotors’ Internalization in 2D Culture

To assess nanomotor’s internalization in cancer cells, CLSM and flow cytometry analyses were performed. HeLa cells were seeded in 6-well plates at a density of 300,000 cells mL^–1^ in DMEM high glucose with 10% FBS and incubated at 37 °C for 24 h and 5% CO_2_. The cells were then treated with NM_Doxo‑GOx_ at 50 μg mL^–1^ for 30 min and incubated for 1, 4, or 6 h.

#### Nanoparticles’ Cytotoxicity in 2D Culture

To evaluate nanoparticles’ cytotoxic activity on cancer cells, WST-1 viability assays were performed. HeLa cells were seeded in 96-well plates at a density of 35,000 cells mL^–1^ DMEM high glucose with 10% FBS to be incubated at 37 °C for 24 h and 5% CO_2_. 4T1 and SK-Mel-103 cells were seeded in the same conditions but at a density of 50,000 cells mL^–1^. For the assays in the absence of fuel, the medium was replaced with DMEM without glucose. Treatments NM_Doxo‑GOx_, NM_Doxo‑GOx‑IN_, NA, NM_GOx_ and NM_Doxo‑BSA_ were added at different concentrations (0, 2, 5, 10, and 15 μg mL^–1^) and allowed to incubate for 30 min. After these times, the noninternalized nanoparticles were removed by a washing step, and cells were incubated under the usual conditions for 24 h. Last, cell proliferation reagent WST-1 was added for 1 h and the absorbance signal was measured at 440 nm. To assess the cytotoxicity of PtNds, the same procedure was followed but using concentration from 0.4 to 3 μg mL^–1^.

#### ROS Production and Detection in 2D HeLa Culture

The ROS formation in HeLa cells induced by treatment with nanomotors was evaluated using the fluorescent ROS detection probe, DCFDA-H2DCFDA (λ_exc_ = 495 nm, λ_em_ = 529 nm). HeLa cells were seeded in a 6-well plate at a density of 170,000 cells mL^–1^ in DMEM high glucose +10% FBS and incubated for 24 h at 37 °C and 5% CO_2_. Afterward, the medium of half the wells was changed to DMEM without glucose. Cells were treated with NM_GOx_ (50 μg mL^–1^) for 30 min at 37 °C. Then cells were washed with PBS to remove non endocytic nanomotors and were incubated for 5, 15, 30, or 60 min. The DCFDA in PBS (5 μM) was added and incubated for 30 min. The ROS detection probe was removed, and the appropriate medium was added to wells and incubated at 37 °C for another 30 min period to stabilize the fluorescence signal. Finally, Hoechst 33342 was added and CLSM images were obtained.

#### 3D Tumor Spheroids Formation

To prepare HeLa 3D multicellular spheroids, we followed the literature guidelines with some modifications.^83^ In the first place, 96-well plates were coated with 50 μL of 1.5% agarose in PBS per well. Agarose was allowed to solidify to form a concave surface, and plates were stored at 4 °C until used. Then 200 μL of HeLa cells were seeded at 10,000 cells mL^–1^ in the generated cavity and incubated at 37 °C and 5% CO_2_ for 4 days. Finally, successful 3D tumor spheroids formation was confirmed by optical microscopy.

#### Nanoparticles’ Cytotoxicity in 3D Tumor Spheroids

To test whether nanomotors were capable of effectively delivering doxorubicin in 3D HeLa spheroids, NM_Doxo‑GOx_ was incubated with them at a concentration of 50 μg mL^–1^ in DMEM high glucose with 10% FBS at 37 °C and 5% CO_2_ for 4 h. DNA marker Hoechst3342 was added and imaged by CLSM. Next the cytotoxic effect was also studied. Spheroids were incubated with nanoparticles NM_Doxo‑GOx,_ NM_Doxo‑GOx‑IN_, NA, NM_GOx_ and NM_Doxo‑BSA_ at concentrations from 0 to 15 μg mL^–1^ in DMEM high glucose supplemented with 10% FBS at 37 °C and 5% CO_2_ for 24 h. The next day, WST-1 was incubated for 3 h and absorbance at 440 nm was recorded.

#### Nanoparticles’ Penetration in 3D Tumor Spheroids

The tumor penetration ability of nanomotors was studied by confocal microscopy. Spheroids were treated with NM_Doxo‑GOx_ or NM_Doxo‑GOx‑IN_ at a concentration of 50 μg mL^–1^ during 24 h. Then, Hoechst (1 μg mL^–1^ incubation for 3 h) was added to label the nuclei of the cells. CLSM images were obtained on a Leica TCS SP8MP multiphoton microscope and fluorescence quantification was performed with the ImageJ Software (expressed as fluorescence intensity per area). Note that due to the high density and compactness of the spheroids only about 1/3 of the total structure is visible with this technique.

### In Vivo Antitumoral Effect of Nanomotors

#### Animal Model

Female (6-week-old) Balb/c nude mice were acquired from Charles River Laboratories and housed at the Príncipe Felipe Research Centre (València, Spain) under pathogen-free conditions. Animals were left in a room at controlled temperature inside ventilated polyethylene cages with stainless steel lids, were administered food and water *ad libitum* and subjected to alternate light/dark cycles lasting 12 h. Mice were treated according to the Guide for Care and Use of Laboratory Animals. All the procedures were approved by the Ethical Committee for Research and Animal Welfare of the Generalitat Valenciana, Conselleria d’Àgricultura, Medi Ambient, Canvi Climàtic i Desenvolupament Rural (2021/VSC/PEA/0070).

#### Tumor Formation

To establish the tumor-bearing mouse model, mice were first anesthetized with isoflurane prior to cancer cell inoculation. Then 1 million HeLa cells resuspended in Matrigel (50% in DMEM) were subcutaneously injected into both flanks of each mouse. Next animals were randomly separated into different experimental groups. Tumor size (length × width) was monitored with an electronic caliper every 2 days. Tumor volume (mm^3^) was estimated by eq 11.

9 $$\mathrm{tumor}\;\mathrm{volume}=\frac{\left(\mathrm{length}\cdot \left({\mathrm{width}}^{2}\right)\right)}{2}$$

#### Mice Treatments

In the early stage cancer model, treatments began 13 days after injecting HeLa cells (set as day 0), when tumor size was approximately 25 mm^3^. The experimental groups were the control, not treated; the final nanomotor, NM_Doxo‑GOx_; the control nanomotor without doxorubicin, NM_GOx_; the nonmotile control nanoparticle NM_Doxo‑BSA_; *n* = 6. Different nanodevices were administered by subcutaneous injection around the tumor at a dose of 15 μg mouse^–1^ (injection volume 25 μL tumor^–1^, solvent PBS 100 mM, pH 7.5) twice weekly, for 4 weeks. On day 28, animals were euthanized by CO_2_ atmosphere, and blood and tumors were collected for further analyses. In the advanced-stage cancer model, treatments were initiated on day 63 after cell inoculation surgery (set as day 0), when tumor size reached approximately 70 mm^3^. The experimental groups were the control, not treated; the final nanomotor, NM_Doxo‑GOx_; and the passive control nanoparticle NM_Doxo‑GOx‑IN_; *n* = 9. The nanoparticles were administered three times during 1 week at a dose of 15 μg mouse^–1^ (injection volume 25 μL tumor^–1^, solvent PBS 100 mM, pH 7.5). Animals were euthanized on day 11 by CO_2_ atmosphere, and tumors were collected for histological analyses.

Highlight that the weight and welfare of the animals were evaluated during both experimental procedures according to the criteria of the Morton and Griffiths scale.

#### Tumor Penetration of Nanomotors by TEM

The evaluation of the cellular uptake of nanomotors was performed by TEM. HeLa cells were seeded on a chamber slide at a density of 350,000 cell mL^–1^ and incubated for 24 h according to the usual parameters. Then cells were treated with NM_GOx_ at a concentration of 50 μg mL^–1^ for 30 min and were subsequently washed with 100 mM PBS, pH 7.4. Next cells were incubated 24 h at 5% CO_2_ and 37 °C. For fixation purposes, cells were mixed with a solution of 3% glutaraldehyde for 2 h at room temperature. Cells were then washed with 100 mM PB and dehydrated in ethanol. To finish, samples were stained with 1% uranyl acetate and 1% osmium tetroxide, included in Araldite epoxy resin, and sectioned into 50–70 nm nanoslices.

The procedure followed to obtain the 3D tumor spheroids TEM images was similar, but the concentration of the treatment with J-Pt or NM_GOx_ was increased to 150 μg mL^–1^, and fixation was carried out with a mixture of 2.5% glutaraldehyde and 2% paraformaldehyde for 1 h at room temperature, and thereafter for 24 h at 4 °C. The protocol followed for the TEM imaging in the early stage cancer model was the same, except that the collected tumors were first divided in half and then cut into representative sections of 1 mm^3^ (*n* = 4). To study the nanomotors distribution in the advanced-stage cancer model, the tumor was divided into 4 representative areas and the same procedure was used. To estimate the number of nanoparticles per cell in spheroids and tumor slices, the cell counter plugging of ImageJ was employed by quantifying at least five images per experimental group.

#### Doxorubicin Detection in Tumors

For *in vivo* doxorubicin detection purposes, the excised tumors were first divided in half and later were embedded in OCT solution and frozen on dry ice. Tumors were cut into 10 μm cryosections, mounted on glass slides and stained with Hoechst 33342 in the dark. Images were obtained by CLSM (λ_ex_ = 470 nm, λ_em_ = 555 nm, magnifications 20×, 40×, and 63×). The mean doxorubicin fluorescence intensity was determined by scanning tumor sections in an Aperio Versa 200 equipment (magnification 10×) and quantifying at least three random regions of each tumor section with the Aperio Image Scope software (*n* ≥ 4).

#### Evaluation of Apoptosis in Tumors

To evaluate the effect of the different nanoparticles on apoptosis in tumors, a TUNEL assay was performed. Tumors were embedded in OCT, frozen on dry ice, sliced into 10 μm sections, mounted on glass slides, and fixed, permeabilized and labeled in accordance with *In Situ* Cell Death Detection Kit (Merck) indications and later with Hoechst 33342 in the dark. Images were obtained by CLSM (λ_ex_ = 488 nm, λ_em_ = 550 nm) at the 20×, 40×, and 63× magnifications. Tumor sections were scanned by Leica Aperio Versa 200 equipment at the 10× magnification, and the number of apoptotic cells was assessed with the Aperio Image Scope software by randomly selecting at least three zones of the samples (*n* = 4).

#### Evaluation of Hypoxia in Tumors

To evaluate the effect of treatment with nanoparticles in the reversal of hypoxia in tumors, the EF5 Hypoxia Detection Kit (Merck) was used. Tumors were embedded in OCT, frozen on dry ice, sliced into 10 μm sections, mounted on glass slides, and fixed, permeabilized and labeled in accordance manufacture indications and later with DAPI in the dark. Images were obtained by CLSM (λ_ex_ = 488 nm, λ_em_ = 550 nm) at the 10× and 40× magnifications (*n* = 2).

#### ROS Generation Detection in HeLa Cells and Tumors

The same procedure as for cell cultures was used to detect ROS *in viv*o. Tumors were weighed, mechanically disaggregated in PBS, and incubated with the DCFDA probe (5 μM) at 37 °C for 30 min. Then the fluorescent signal was monitored in a Wallace 1420 workstation (*n* = 4). H_2_O_2_ was used as positive control. The results were normalized according to the percentage of live cells.

#### Analysis of Platinum Biodistribution

Lungs, heart, liver, spleen, bladder kidneys, and tumor of mice were extracted to study the biodistribution of Pt. The organs were weighed and digested in a microwave oven. Then, Pt levels were measured by Inductively Coupled Plasma Mass Spectrometer System (ICP-MS) using an Agilent model 7900. Data are expressed as mg Pt per kg tissue.

#### Analysis of NM_Doxo‑GOx_ Toxicity

To evaluate the biocompatibility of the nanomotor in mice, organs (lungs, heart, liver, spleen and kidneys) were extracted, fixed with 4% paraformaldehyde, embedded in paraffin, cut in 5 μm sections and stained with hematoxylin and eosin. To determine apoptosis levels, organs were labeled with *In Situ* Cell Death Detection Kit (Merck). Moreover, blood samples were collected and subjected to biochemical and hematological analyses.

### Evaluation of Nanomotors Efficacy in Patient-Derived Organoids

#### Organoid Formation and Maintenance

A naïve-tissue sample was obtained from a core needle biopsy of a triple-negative breast cancer patient at Hospital Clínico of València. The proper informed consent was signed by the patient. Tumor fragment was then implanted in the mammary fat of 6 weeks to 3 months-old NOD/SCID female mice (Charles River Laboratories, Wilmington, MA). The tumor was allowed to grow until it reached the maximum size of 1 cm^3^ and organoid formation was then performed. Briefly, the tumor was washed for 10 min at room temperature with 1% penicillin/streptomycin (Biowest, France), cut into small pieces, and digested with patient-derived organoid (PDO) media (Table S7) containing 300 U mL^–1^ collagenase I (Gibco) and 100 U mL^–1^ hyaluronidase I (Sigma-Aldrich) on an orbital shaker at 37 °C for 1 h and 10 min. Tumor fragments were then centrifuged at 300*g* for 5 min and erythrocytes were lysed with 2 mL of ACK lysis buffer (Gibco) for 3 min at room temperature before the addition of 10 mL of PDO media and centrifugation at 300*g* for 5 min. The sample was resuspended in 10 mL of PDO media and strained by using a 200 μm cell strainer (pluriSelect, Germany) before centrifugation at 300*g* for 5 min. After that, 5 × 10^5^ to 1 × 10^6^ cells were embedded in 200 μL of growth factor-reduced Matrigel (Corning) domes on a tissue-treated 6-well plate. PDO media was changed twice a week and passage was performed every 7–14 days. To passage PDO, Matrigel was digested with a Dispase solution of 80% (Stemcell Technologies, Canada), 20% of FBS (Gibco), and 10 μM of Y-27632 (Stemcell Technologies, Canada) for 20 min at 37 °C before centrifugation at 300*g* for 5 min. The organoids were then dissociated into single cells with TrypLE Express (Gibco) for 10 min at 37 °C and centrifuged at 300*g* for 5 min. Finally, 2 × 10^5^ cells per well were seeded in a dome of 200 μL of growth factor-reduced Matrigel (Corning) on a tissue-treated 6-well plate. All experiments were approved by the Institutional Review Board of Biomedical Research Institute INCLIVA (Valencia, Spain) (2021/VSC/PEA/0260).

#### PDO Penetrability Assay

2000 Organoids were embedded in 30 μL growth factor-reduced matrigel (Corning) domes on an 8-well chambered coverslip (Ibidi GmbH). The following day, organoids were treated with 50 μg mL^–1^ of NM_Doxo‑GOx_ or NM_Doxo‑GOx‑IN_ for 2 h and fixed with 4% PFA for 10 min at 37 °C. Then, nuclei were stained with Hoechst 33342 (Invitrogen) for 4 h. Doxorubicin was analyzed by confocal microscopy using the Leica TCS SP8 X White Light Laser Confocal Microscope (Leica Microsystems, Wetzlar, Germany) at the Central Medicine Research Unit of Universitat de València (UCIM-UV). Pictures were captured with an HC PL APO CS2 20x/0.75 IMM objective and recorded in sequential order with an excitation of 405 and 546, and an emission of 410–600 and 584–637 for doxorubicin and Hoechst, respectively. The doxorubicin mean fluorescence intensity per organoid was quantified using ImageJ Software (version 1.51h, NIH). For each condition, a minimum of 20 organoids were analyzed, and the fluorescence intensity of NM_Doxo‑GOx_ was normalized for NM_Doxo‑GOx‑IN_.

#### Organoids Viability Assay

PDOs were digested to obtain a single-cell suspension. Then, 10^4^ cells well^–1^ were embedded in 10 μL of 50% of growth factor-reduced Matrigel (Corning) domes on a 96-well plate. Once organoids were formed, they were treated with 2, 5, 10, 15, or 30 μg mL^–1^ of NM_Doxo‑GOx_, NM_GOx_ or NM_Doxo‑GOx‑IN_ for 24 h. Untreated organoids were also included as a control. In a separate plate, 4 wells were seeded as a day 0 seeding control. Plates were then incubated with CellTiter-Glo 3D Cell Viability Assay (Promega) according to the manufacturer’s protocol. The well contents were then transferred to a black polystyrene 96-well plate and measured in the luminometer Promega GloMax Plate Reader (Promega). Raw values were normalized to day 0 seeding control and 100% viability was established for the control condition.

### Statistical Analysis

A statistical analysis of the results was carried out with the GraphPad Prism 8 software. A comparison between the different groups was made by a one/two-way ANOVA test, followed by Tukey’s post tests. *p*-Values below 0.05 were considered statistically significant and were indicated by an asterisk (* *p* < 0.05, ** *p* < 0.01, *** *p* < 0.001, **** *p* < 0.0001).

## Supplementary Material







## Notes

Materials, description of other nanomotors employed in anticancer therapy (Table S1), description of the nanomaterials used in this study (Table S2), PA structure, size distribution by TEM, FE-SEM images, PXRD patterns, N_2_ adsorption–desorption isotherms, FTIR and TGA, hydrodynamic diameter, ζ-potential, cargo-controlled delivery in response to lysosomal components and different protease concentrations, STEM-EDX analysis after drug-release process, GOx activity assay, peroxidase-like activity test, glucose and H_2_O_2_ consumption, O_2_ generation, nanomotors’ trajectories, motion characterization of control nanoparticles, characterization of H_2_O_2_-triggered motion, cell internalization by TEM, ROS generation, doxorubicin delivery in 3D tumor spheroids, tumor penetration of nanomotors by TEM, CLSM and flow cytometry, biodistribution and biosafety analysis, representative image of nanomotors’ quantification and distribution in tumor, tumor volume at the beginning and end of the experiments and determination of hypoxia in tumor (PDF)
Brownian motion of NM_Doxo‑GOx_ recorded by Nanosight (0 mM glucose) (Video S1) (AVI)
Enhanced diffusion of NM_Doxo‑GOx_ in the presence of glucose recorded by Nanosight (25 mM glucose) (Video S2) (AVI)
