# Supplementary material for: Glucose-Fueled Gated Nanomotors: Enhancing In Vivo Anticancer Efficacy via Deep Drug Penetration into Tumors
Source: ACS Nano. 2025 May 30;19(22):20932–55. doi: 10.1021/acsnano.5c03799 (PMC12164516; doi:10.1021/acsnano.5c03799)
Supplement: Supplementary file 1 [file nn5c03799_si_001.pdf]

Supporting Information

Glucose-fueled Gated Nanomotors: Enhancing *In Vivo* Anticancer Efficacy via Deep Drug Penetration into Tumors

Andrea Escudero,[a,b] Francisco J. Hicke,[a,c] Elena Lucena-Sánchez,[a,b,c] Sandra Pradana-López,[a] Juan José Esteve-Moreno,[a,b,c] Víctor Sanz-Álvarez,[a] Iris Garrido-Cano,[a,c,d] Sandra Torres-Ruiz,[d] Juan Miguel Cejalvo,[d,e,f] Alba García-Fernández,[a,b,c] Paula Díez [a,c,g]* and Ramón Martínez-Máñez[a,b,c,g]**

[a]Instituto Interuniversitario de Investigación de Reconocimiento Molecular y Desarrollo Tecnológico (IDM), Universitat Politècnica de València, Universitat de València. Camino de Vera, s/n. 46022, València, Spain.

[b]Unidad Mixta UPV-CIPF de Investigación en Mecanismos de Enfermedades y Nanomedicina, Universitat Politècnica de València, Centro de Investigación Príncipe Felipe. C/ Eduardo Primo Yúfera 3. 46012, València, Spain.

[c]CIBER de Bioingeniería, Biomateriales y Nanomedicina, Instituto de Salud Carlos III

[d]Biomedical Research Institute INCLIVA. C/ de Menéndez y Pelayo, 4, 46010, Valencia, Spain.

[e]Biomedical Research Networking Center in Oncology (CIBERONC), 28029 Madrid, Spain.

[f]Department of Clinical Oncology, University Clinical Hospital of Valencia. Av. de Blasco Ibáñez 17, 46010, Valencia, Spain.

[g]Unidad Mixta de Investigación en Nanomedicina y Sensores. Universitat Politècnica de València, IIS La Fe. Av. Fernando Abril Martorell, 106 Torre A 7ª planta. 46026, València, Spain.

Chemicals

n-cetyltrimethylammonium bromide (CTABr), tetraethyl orthosilicate (TEOS), dihydrogen hexachloroplatinate (H_2PtCl_6), polyvinylpyrrolidone (PVP), ascorbic acid, sodium citrate tribasic dihydrate, paraffin wax, (3-mercaptopropyl)trimethoxysilane, (3-aminopropyl)triethoxysilane (APTES), glucose oxidase from *Aspergillus niger* (GOx) (EC 1.1.3.4), bovine serum albumin (BSA), N-(3-dimethylaminopropyl)-N'-ethylcarbodiimide hydrochloride (EDC), N-hydroxysuccinimide (NHS), 6-aminohexanoic acid, perylene-3,4,9,10-tetracarboxylic acid diimide, imidazole, 4-dimethylaminopyridine (DMAP), triethylamine (TEA), $NaHCO_3$, $MgSO_4$, protease from *Streptomyces griseus*, 2,2′-azino-bis(3-ethylbenzothiazoline)-6-sulfonic acid (ABTS), Dulbecco′s Modified Eagle′s Medium (DMEM), Hoechst 33342, WST-1, isoflurane, and *in situ* cell death detection kit fluorescein were acquired from Sigma Aldrich. Doxorubicin HCl ($C_{27}H_{29}NO_{11}$) was purchased from Carbosynth. Bicinchoninic acid assay (BCA) kit, hydrogen peroxide (30%), fetal bovine serum (FBS), glutaraldehyde, paraformaldehyde, and Matrigel were supplied by Thermo Fisher. DCFDA/H2DCFDA Cellular ROS Assay Kit was provided by Abcam. OCT was obtained from VWR. HeLa cells were purchased from ATCC.

General instruments and methods

The nanoparticle scaffold was characterized by standard methods for nanomaterials characterization, like powder X-ray diffraction (PXRD), N_2 adsorption-desorption, thermogravimetric analysis (TGA), Fourier transformed infrared spectroscopy (FTIR), dynamic light scattering (DLS), transmission electron microscopy (TEM) and scanning transmission electron microscopy coupled with electronic energy dispersive x-ray spectroscopy (STEM-EDX). PXRD analysis was performed on a D8 Advance Seifert 3000TT diffractometer employing CuKα radiation at low angles ($1.5 < 2\theta > 7$, with steps of 0.04 degrees and 3s for step) and high angles ($35 < 2\theta > 80$ with steps of 0.04 degrees and 1 s for step). Micromeritics TriStar II Plus automated analyzer was used for the N_2 adsorption-desorption isotherms recording, degassing the nanoparticles at 90 or 120 °C under vacuum overnight. The specific surface area was determined using the Brunauer-Emmett-Teller (BET) model from the adsorption data in the low-pressure range. Pore size was calculated by the Barrett-Joyner-Halenda (BJH) model. TGA measurements were performed in a TA Instruments SDTQ600 apparatus in an oxidizing atmosphere (air, 80 mL min-1) and heating rate program between 393-1273 °C at 10 °C min^{-1} followed by an isothermal heating step at 1273 °C for 30 min. FTIR was performed in a Tensor 27 instrument (Bruker). DLS and zeta potential analysis were carried out on a ZetaSizer Nano ZS (Malvern). TEM images of nanoparticles were acquired on a JEOL TEM-2100F electron microscope, and STEM-EDX on a JEM 2100F instrument. Additionally, nanomotors motion was analyzed in a Nanosight NS300 (Malvern) and nanomotors catalytic activities on a JASCO V-650 spectrophotometer and a portable waterproof oximeter (Hanna Instruments). For nanomotors drug delivery assays a Thermo-shaker HC24N Grant Instruments PCMT was used to shake the samples, and a JASCO FP-8500 spectrophotometer was employed to measure the fluorescence emission. Confocal laser scanning microscopy (CLSM) images were obtained with a Leica TCS SP8 AOBS inverted microscope and tissue fluorescent images were acquired with a digital pathology research scanner, Leica Aperio Versa. TEM images of cells and tissues were obtained with a FEI Tecnai Spirit G2 microscope operating at 80 kV with a digital camera. Cytotoxicity studies were carried out in a Wallac Victor2TM spectrophotometer.

Table S1. Comparison of the nanodevice $NM_{Doxo\text{-}GOx}$ with other recently published chemically driven nanomotors for cancer treatment using *in vivo* models.

Description of the nanomaterial design and antitumoral strategy	Fuel and driving system	Model complexity	Treatment effect	
**Janus Au-MnO_2.** Gas therapy with SO_2 obtained from BTS and MnO_2 decomposition. No chemotherapy agent delivery.	Catalysis of endogenous **H_2O_2** (0.1 mM) into O_2 by MnO_2.	B16 tumor-bearing C57BL6 mice.	Tumor volume reduction and increase in the survival, apoptosis, and necrosis rates.	(1)
**Zwitterionic nanoparticles loaded with cysteine and α-CHCA**. Induction of acidosis. No chemotherapy agent delivery.	Transformation of **cysteine** to H_2S by endogenous CBS.	MCF-7 tumor-bearing Balb/c mice.	Tumor penetration, tumor volume reduction and increase in the apoptosis rate and necrotic area.	(2)
**Cationic gold nanoclusters decorated with GOx and catalase.** Hypoxia alleviation and HK-2 siRNA delivery to inhibit glycolysis. No chemotherapeutic release.	Decomposition of **glucose** by GOx and **H_2O_2** by catalase.	4T1 tumor-bearing Balb/c mice.	Relief of hypoxia, inhibition of aerobic glycolysis and reduction in tumor volume and lung metastasis.	(3)
**Hydroxyapatite nanorods functionalized with urease and hyaluronidase and loaded with Doxo.** Doxo delivery and ECM digestion.	Transformation of **urea** (5 mM) into CO_2 and NH_3 by urease.	4T1 tumor-bearing Balb/c mice.	Survival prolongation, tumor growth inhibition and no tumor metastasis to lungs.	(4)
**Heparin/folic acid nanoparticles loaded with L-arginine and Doxo.** Doxo delivery and ECM digestion by $ONOO^-$.	L-arginine transformed to **NO** by endogenous NOS.	MCF-7 tumor-bearing Balb/c mice.	Penetration and reversal of multidrug resistance in tumor.	(5)
**Heparin folate nanoparticles loaded with L-arginine, Doxo and anti-PD1.**	L-arginine transformed to **NO** by endogenous NOS.	B16F10 tumor-bearing C57BL/6 mice.	Tumor penetration, infiltration of T-cells	(6)

Docetaxel delivery, ECM degradation and immune cells tumor infiltration.				
**Janus Ag-polymer.** Cisplatin delivery.	Decomposition of **H_2O_2** (1 mM) by Ag.	4T1 tumor-bearing Balb/c mice.	Tumor growth inhibition.	(7)
**Janus sphere Pt-MSN functionalized with a copper chelator and loaded with a photosensitizer.** Cooper deficiency, hypoxia reversion and PDT.	Decomposition of endogenous **H_2O_2** by Pt.	LoVo tumor-bearing nude mice.	Damage to tumor tissue and metastasis inhibition.	(8)
**Pt nanoflowers.** Doxo delivery.	Decomposition of **H_2O_2** (10 mM) by Pt.	4T1 tumor-bearing Balb/c mice.	Reduction in tumor volume in combination with radiotherapy: sensitization effect.	(9)
**Janus Au-Pt nanorod.** Oxidation to Pt^{2+} and formation of Pt-DNA adducts.	Decomposition of **H_2O_2** (200 µM) by Pt.	MCF-7 tumor-bearing mice.	Photoacoustic visualization and tumor volume reduction.	(10)
**Janus Au functionalized with PEG and urease. Exhibit movement in serum.**	Transformation of **urea** (10 mM) into CO_2 and NH_3 by urease.	Microfluidic channel with an agarose gel containing urea.	Biosafety in MCF-7 cells and *in vivo*.	(11)
**$NM_{Doxo-GOx}$.** Glucose-induced movement and intracellular Doxo controlled-release.	Enzymatic transformation of **glucose** into H_2O_2 and subsequent decomposition by PtNds.	HeLa tumor-bearing mice and cancer patient-derived organoids.	Enhanced nanomotors penetration and Doxo delivery resulting in significant tumor growth inhibition.	This work

Table S2. Summary of the nanoparticles employed in this work.

Nanoparticle	Code	Description	
Janus Pt-MSN	J-Pt	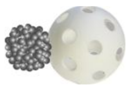	Inorganic scaffold of the nanomotor without functionalization
Janus Pt-MSN-(-)-GOx	NM_{GOx}	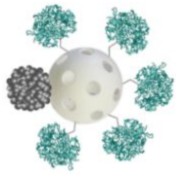	Control nanomotor: empty Janus Pt-MSN capped with GOx. **Self-propulsion but not drug delivery**.
Janus Pt-MSN-(Doxo)-BSA	$NM_{Doxo\text{-}BSA}$	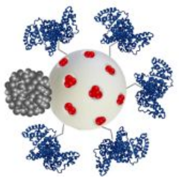	Control: nanoparticle without the first propellent element, GOx. **Drug delivery but not self-propulsion.**
Janus Pt-MSN-(Doxo)-GOx with GOx activity inactivated by heat	$NM_{Doxo\text{-}GOx\text{-}IN}$	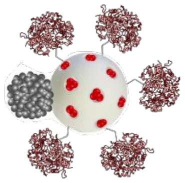	Control: nanoparticle with the first propellent element, GOx inactivated. **Drug delivery but not self-propulsion**.
Janus Pt-MSN-(-)-PA-GOx	$NM_{PA\text{-}GOx}$	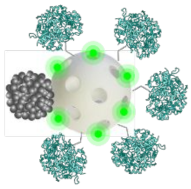	Control nanomotor: PA-labelled nanomotor capped with GOx but not loaded with Doxo. **Used for CLSM motion studies**.
Janus Pt-MSN-(-)-PA-GOx with GOx activity inactivated by heat	$NM_{PA\text{-}GOx\text{-}IN}$	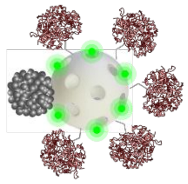	Control: PA-labelled nanomotor capped with GOx but inactivated by heat. No self-propulsion. **Used for CLSM motion studies**.
PtNds, Doxo and GOx	NA	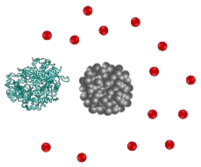	Catalytic elements and drug content of the final nanomotor but **not assembled**.

Janus Pt-MSN-(Doxo)-GOx	**$NM_{Doxo-GOx}$**	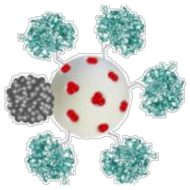	**Final nanomotor**: Janus Pt-MSN loaded with Doxo and capped with GOx. **Drug delivery & self-propulsion**.

1. Structural characterization of nanoparticles

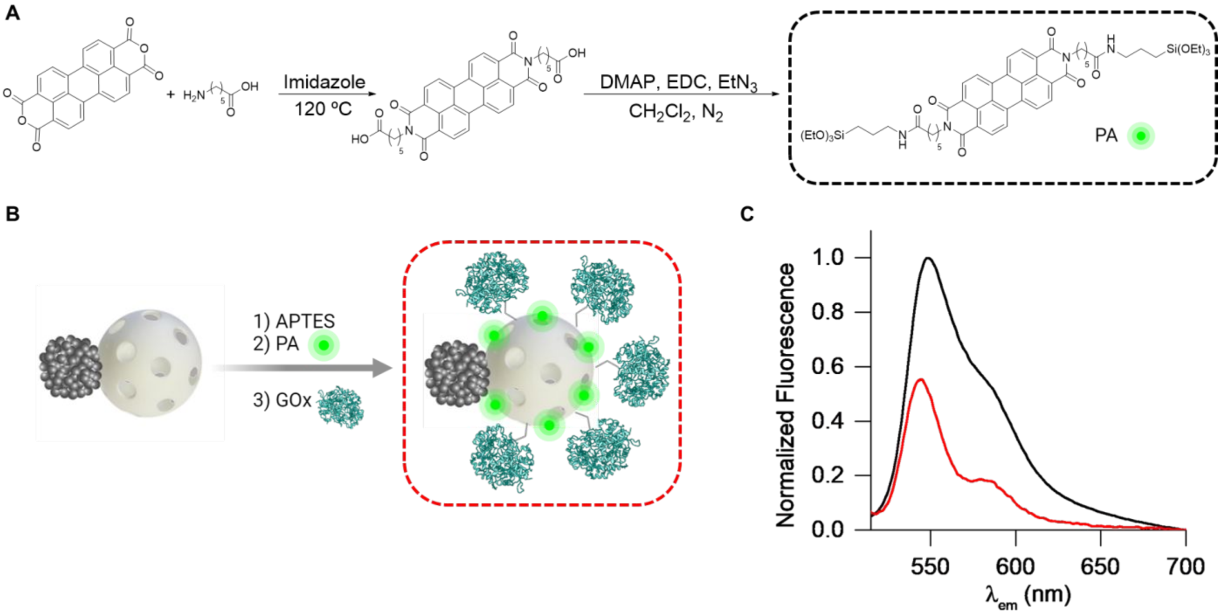

Figure S1. A) Scheme of the synthesis process of the H_2O_2-resistant fluorophore PA. B) Scheme of the procedure followed to construct the nanomotor NM_{PA-GOx}. C) Fluorescence emission spectrum of PA (black) and NM_{PA-GOx} (red) in PBS (λ_{exc} = 490 nm).

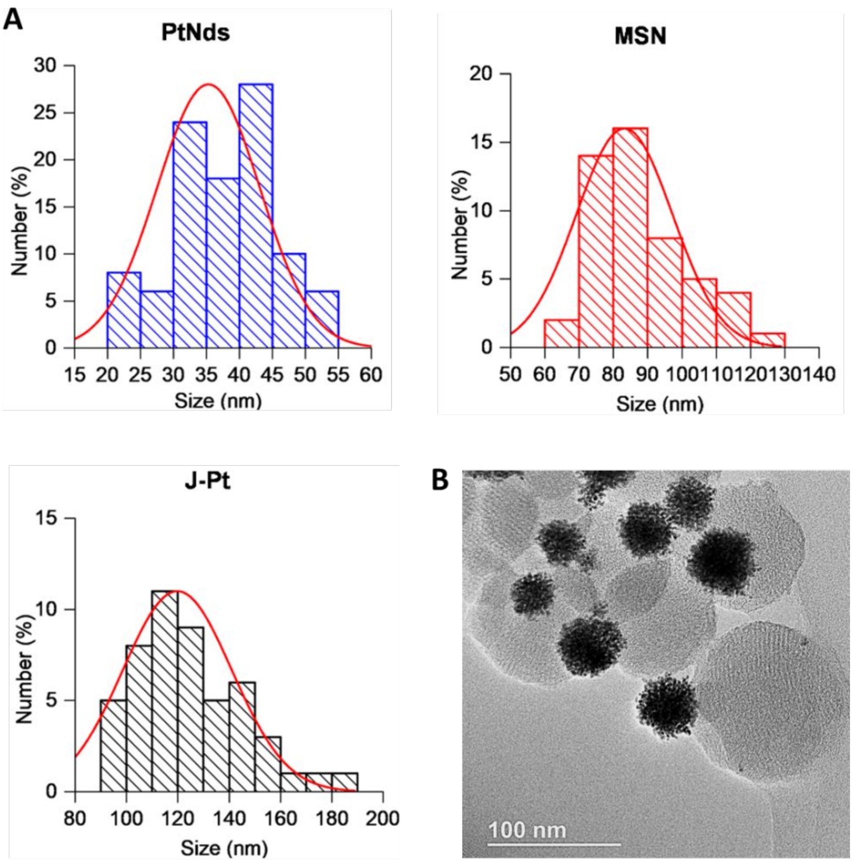

Figure S2. A) Size distribution of PtNds (blue), MSN (red) and J-Pt (black) (determined by quantifying the dimensions of 50 nanoparticles by TEM). B) Representative image of J-Pt showing the snowman-like nanoarchitectonics with a 1:1 ratio of Pt-MSN (Scale bar: 100 nm).

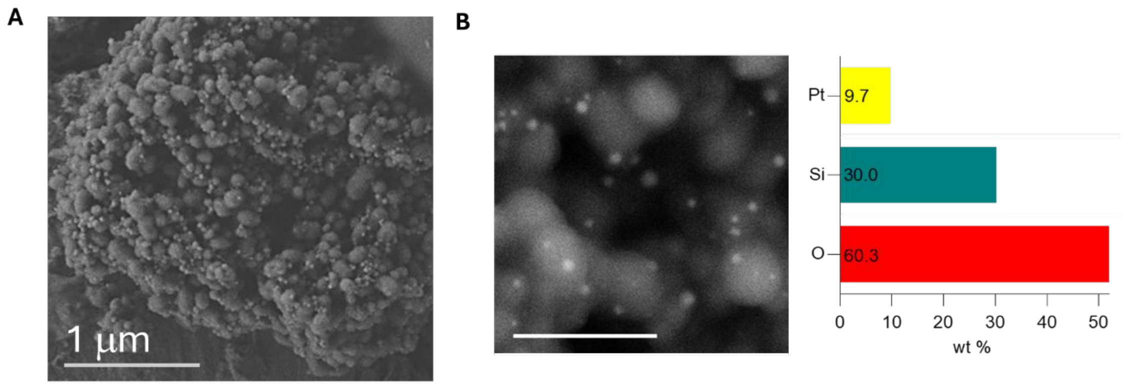

Figure S3. A) FE-SEM images of J-Pt nanoparticles, showing their surface morphology with successful conjugation between MSN and PtNds. B) STEM-EDX analysis of J-Pt

confirming the presence of O (red, 60.3 %), Si (green, 30.0 %) and Pt (yellow, 9.7 %) atoms, (scale bar: 200 nm).

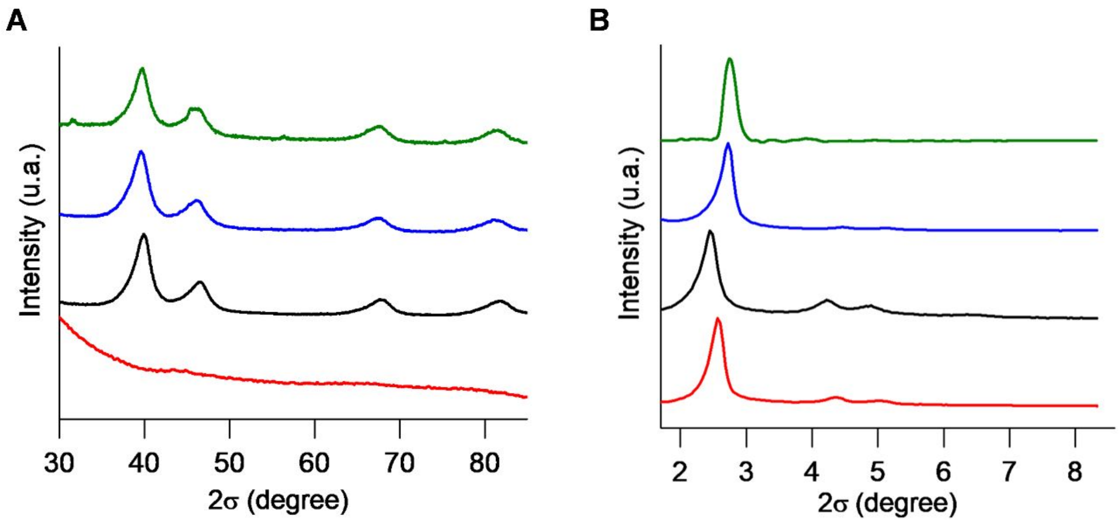

Figure S4. PXRD patterns of calcined MSNs (red), J-Pt (black), NM_{GOx} (blue) and $NM_{Doxo\text{-}GOx}$ (green) at high (30 < 2θ > 85) (A) and low (1.5 < 2θ > 8) (B) angles.

All nanoparticles showed the characteristic Bragg peak of mesoporous materials with MCM-41 structure at around 2.6° indexed as plane (100). In addition, they present two less intense peaks, at approximately 4.1 and 4.7 °, corresponding to planes (110) and (200). These results confirmed that the joining of PtNds and MSNs to build J-Pt as well as the functionalization of its surface (NM_{GOx}) and the loading of the pores ($NM_{Doxo\text{-}GOx}$) did not have an impact on the mesoporous structure. Besides, J-Pt, NM_{GOx} and $NM_{Doxo\text{-}GOx}$ exhibited Bragg peaks indexed at (111), (200), (220) and (311) reflecting the cubic structure of the Pt nanoparticles.

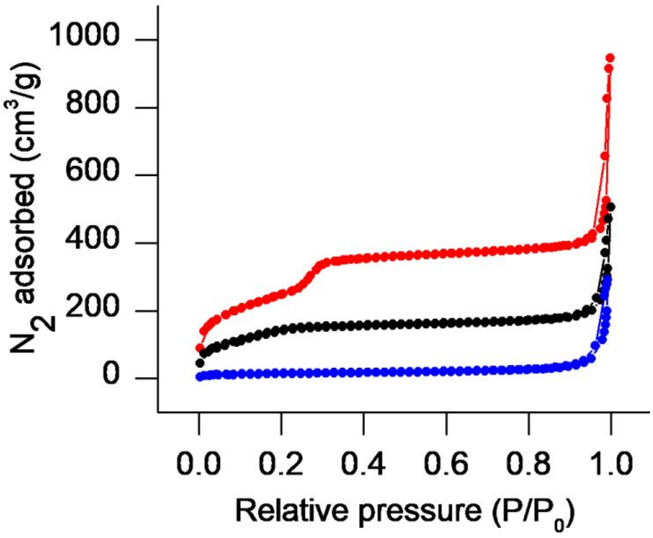

Figure S5. N_2 adsorption-desorption isotherms of calcined MSNs (red), J-Pt (black) and NM_{GOx} (blue).

N_2 adsorption-desorption isotherms of MSNs and J-Pt presented a main adsorption step from 0.2 to 0.4 P/P_0 values, which corresponds to N_2 condensation inside the empty pores by capillarity. In addition, the absence of hysteresis loop pointed to the cylindrical uniformity of the pores. However, in NM_{GOx} the ~ 0.3 P/P_0 adsorption step disappeared, indicating a significant decrease of the gas volume adsorbed. This finding confirmed the correct blocking of the pores with the gatekeeper GOx. The adsorption step at 0.9 P/P_0 present in all isotherms indicated textural porosity.

Table S3. Textural properties (pore volume, pore size, and specific surface) of calcined MSNs, J-Pt and NM_{GOx} calculated from the N_2 adsorption-desorption isotherms applying the models BJH and BET.

Nanoparticle	Pore volume (BJH) (cm^3 g^{-1})	Pore size (BJH) (nm)	Specific surface (BET) (m^2 g^{-1})
**MSNs**	0.8	2.7	997.9
**J-Pt**	0.1	2.3	551.2
**NM_{GOx}**	**0.03**	**-**	**49.6**

BET results showed that the specific surface area was decreased in J-Pt compared to MSNs, which is associated to the attachment of PtNds on one side of the nanoparticles. In NM_{GOx} the surface area was even lower than in J-Pt due to the functionalization with the gatekeeper GOx that blocks the pores.

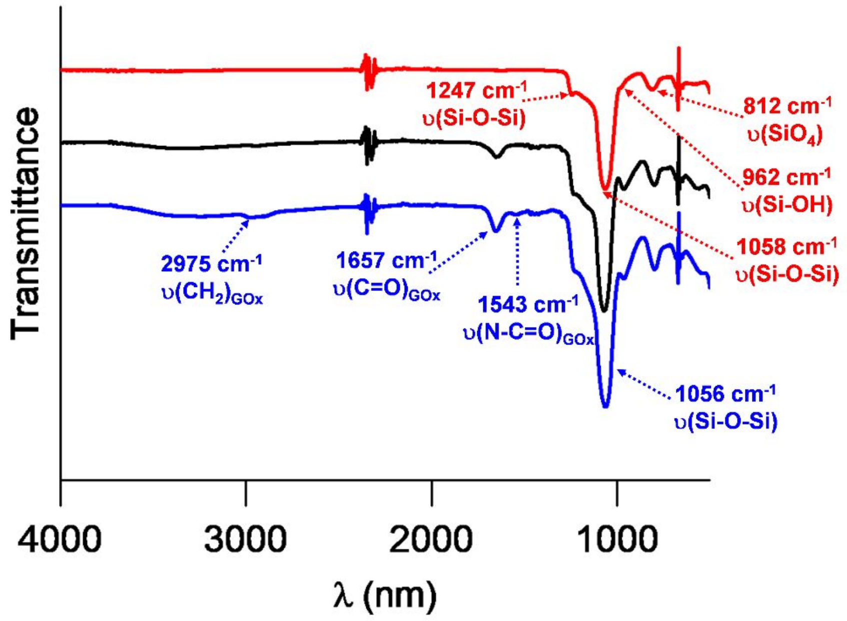

Figure S6. FTIR spectrum of calcined MSNs (red), J-Pt (black) and NM_{GOx} (blue).

In the FTIR spectra of NM_{GOx}, MSN and J-Pt showed the bond stretching vibrations characteristic of siliceous materials. The peaks at 962 cm^{-1} were attributed to Si-OH bonds, the bands at 812 cm^{-1} corresponded to SiO_4 tetrahedra, and the bands at 1058 cm^{-1} and 1247 cm^{-1} (shoulder) were attributed to Si-O-Si bonds. In addition, the NM_{GOx} spectrum showed an absorption band of amide I at 1657 cm^{-1}, a peak at 1543 cm^{-1} corresponding to the stretching vibration of the C-N bond, as well as a band assigned to CH_2 groups at 2975 cm^{-1}. All of them indicated the presence of the GOx enzyme attached to the surface of the nanomotor.[12]

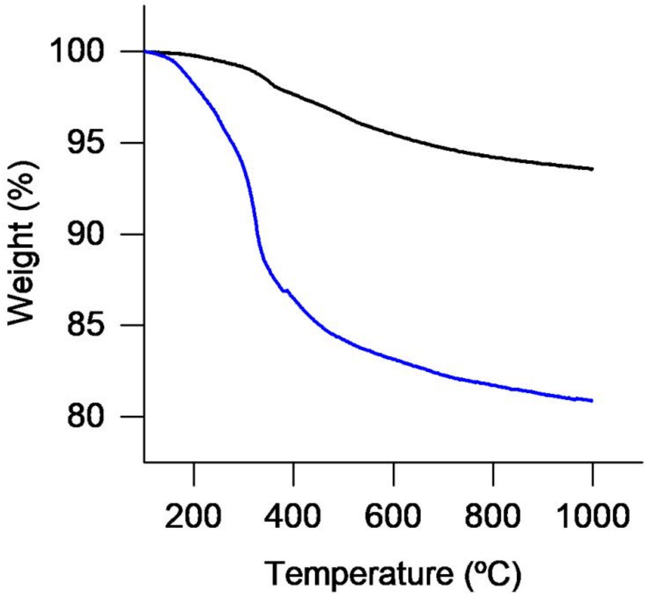

Figure S7. TGA analysis of J-Pt (black) and NM_{GOx} (blue).

In the NM_{GOx} TGA analysis, the thermal decomposition of the organic content confirmed the correct functionalization with the enzyme GOx as capping system (calculated to be 121.5 µg *per* mg of nanoparticle).

Table S4. Characterization of nanoparticles: gatekeeper quantity (µg mg nanoparticle $^{-1}$), GOx activity (U mg nanoparticle $^{-1}$) and doxorubicin content (µg mg nanoparticle $^{-1}$).

Nanoparticle name	Gatekeeper (µg mg nanoparticle^{-1})	GOx activity (U g nanoparticle^{-1})	Doxo loaded (µg mg nanoparticle^{-1})
**NM_{GOx}**	100.73	323	**-**
**$NM_{Doxo-BSA}$**	90.39	-	34
**$NM_{Doxo-GOx}$**	**108.05**	**323***	**67**

The drug loading efficiency (DLE) of $NM_{Doxo-GOx}$ was calculated to be 13.4 %, whereas the loading efficiency (LE) relative to the enzyme bound was calculated to be 43 % (**Equation 2**).

* The GOx activity of $NM_{Doxo-GOx}$ was 323 U g^{-1}. However, considering that the amount of GOx immobilized on the nanomotor was 108.5 µg mg^{-1} (deduced by BCA assay), its specific activity was 2977 U g^{-1}. The activity of free GOx was 5480 U g^{-1}, therefore, after its immobilization on the nanoparticles, 54.3% of its activity was retained.

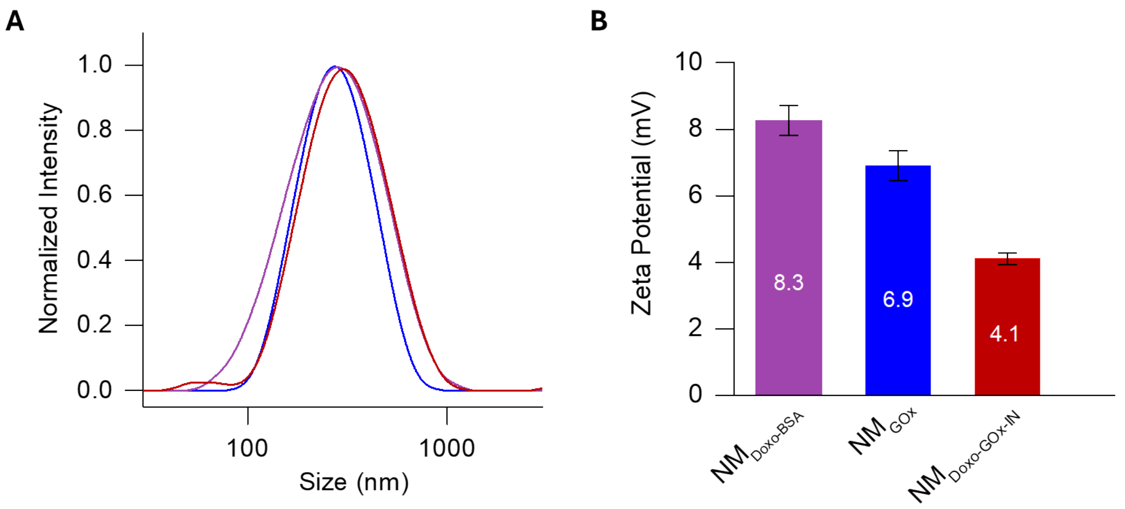

Figure S8. DLS analysis of control nanoparticles NM_{GOx} (blue), $NM_{Doxo\text{-}BSA}$ (purple) and $NM_{Doxo\text{-}GOx\text{-}IN}$ (dark red). A) Hydrodynamic diameter (nm). B) Zeta potential (mV). Nanoparticles were dispersed in deionized water (DI) at a concentration of 1 mg mL^{-1}, sonicated for 5 min and then the supernatant was measured.

The size of the control nanoparticles, $NM_{Doxo\text{-}BSA}$ (256 ± 4 nm), NM_{GOx} (270 ± 5 nm) and $NM_{Doxo\text{-}GOx\text{-}IN}$ (285 ± 4 nm), was comparable to that observed for the final nanomotor $NM_{Doxo\text{-}GOx}$ (271 ± 3 nm). However, we found minor differences in the surface charge values (NM_{GOx}, 6.9 mV; $NM_{Doxo\text{-}BSA,}$ 8.3 mV; and $NM_{Doxo\text{-}GOx\text{-}IN}$, 4.1 mV). The slightly different surface charge observed between the active, $NM_{Doxo\text{-}GOx}$ (9.8 mV), and the inactive, $NM_{Doxo\text{-}GOx\text{-}IN}$ was probably due to the exposure carboxylic groups due to the conformational change caused by the heat inactivation process.

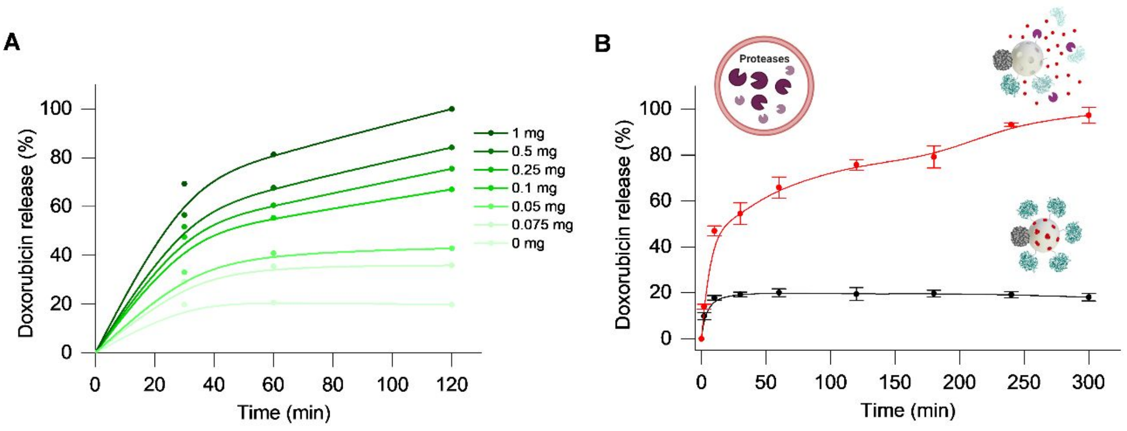

Figure S9. Normalized doxorubicin release profile measured by fluorescence spectroscopy (λ_{em}, 555 nm; λ_{ex}, 470 nm) from $NM_{Doxo\text{-}GOx}$ in the presence of: A) different

protease concentrations (from 0 to 1 mg, increasing intensity of green) of *Streptomyces griseus* to study the enzyme threshold necessary to trigger doxorubicin release, and B) lysosomal extract (red) or PBS (black) (data represent mean ± SD, n = 3).

Graph A shows that concentrations as low as 0.05 mg promote delivery of doxorubicin compared to the control without enzyme (0 mg), which increased proportionally with the protease concentration added until reaching a maximum at 1 mg (concentration used in Figure 1D). Graph B shows that lysosomal proteases trigger the specific release of the cargo from the pores of the nanomotor. Maximum release was found after an incubation of about 300 min, indicating that this is the time required for GOx to be degraded.

2. Analysis of glucose-induced motility of nanomotors

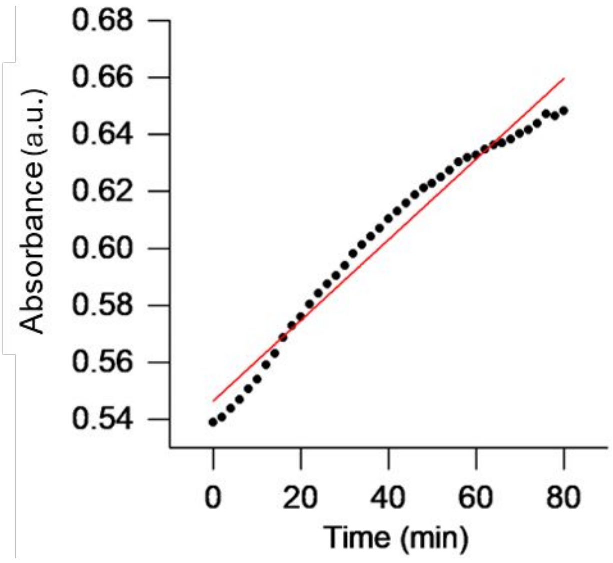

Figure S10. Determination of GOx activity on $NM_{Doxo\text{-}GOx}$. Monitorization of $ABTS^{2-}$ formation as consequence of H_2O_2 generation (absorbance at 405 nm) in function of time. The GOx activity on the nanomotor was estimated to be 32 U *per* g of solid (Equation 3).

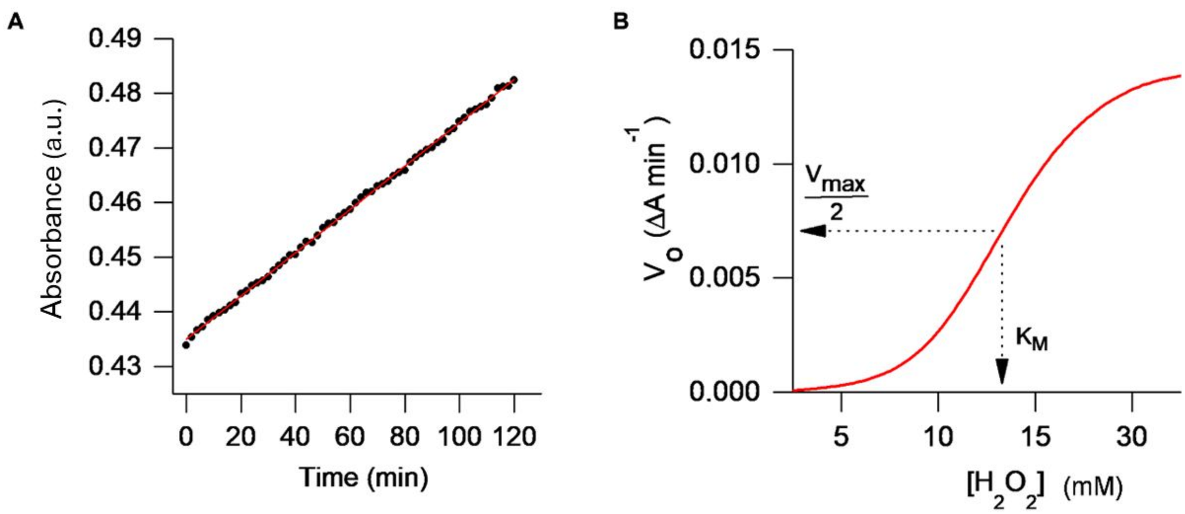

Figure S11. Determination of J-Pt peroxidase-like activity. A) Reaction rate of $ABTS^{2-}$ formation (absorbance at 405 nm *vs* time) upon H_2O_2 addition due to peroxidase-like activity on PtNds face. B) Effect of H_2O_2 concentration on the J-Pt peroxidase-like activity, from which the enzymatic parameters were calculated (**Table S5**).

Table S5. Catalytic efficiency of the J-Pt compared with the values of horseradish peroxidase (HRP) as reference.

Catalytic parameters	J-Pt	HRP[13]
**K_M (mM)**	13	3.7
**V_{max} (M^{-1} s^{-1})**	$2.3 \cdot 10^{-4}$	$8.7 \cdot 10^{-8}$
**K_{cat} (s^{-1})**	$5.2 \cdot 10^{5}$	$3.5 \cdot 10^{3}$
**K_{cat}/K_M (M^{-1} s^{-1})**	$4.7 \cdot 10^{7}$	$9.5 \cdot 10^{2}$

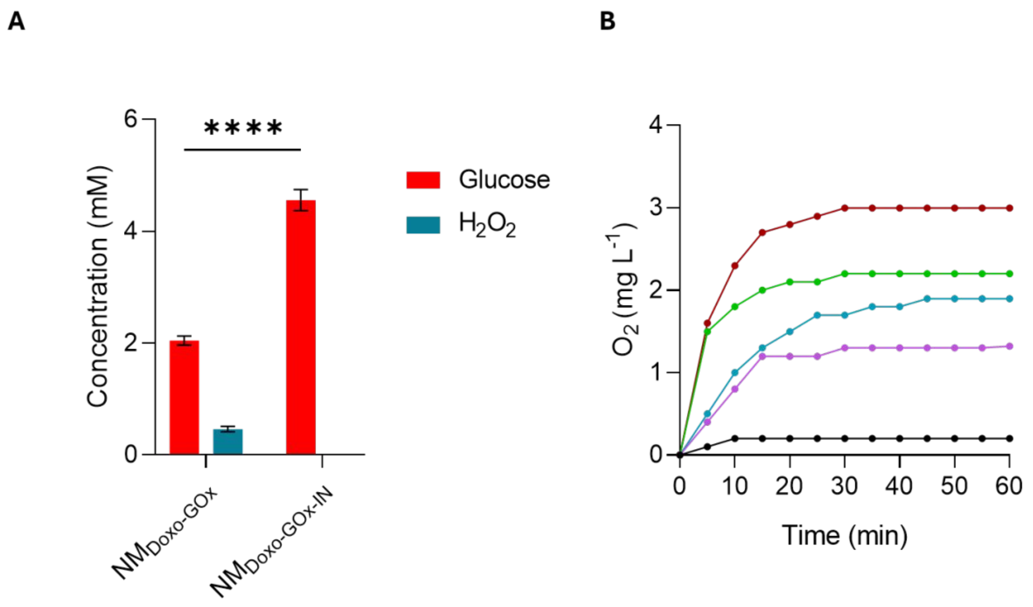

Figure S12. Study of glucose consumption, H_2O_2 production, and dissolved O_2 levels during the catalytic reaction of $NM_{Doxo\text{-}GOx}$. A) Concentration of glucose (red) and H_2O_2 (blue) after 30 min with $NM_{Doxo\text{-}GOx}$ and $NM_{Doxo\text{-}GOx\text{-}IN}$ under 5 mM glucose concentration measured by colorimetric assay with the phenol-sulfuric method for glucose and the Amplifluᵀᴹ Red/HRP assay for H_2O_2 (n = 3). Statistical significance was determined by a two-way ANOVA, (**** $p < 0.0001$). B) Monitoring of dissolved O_2 levels generated by $NM_{Doxo\text{-}GOx}$ using an oximeter over time (60 min) in response to different glucose concentrations (0 mM, black; 2.5 mM, purple; 5 mM, blue; 10 mM, green; 15 mM, red) under hypoxic conditions.

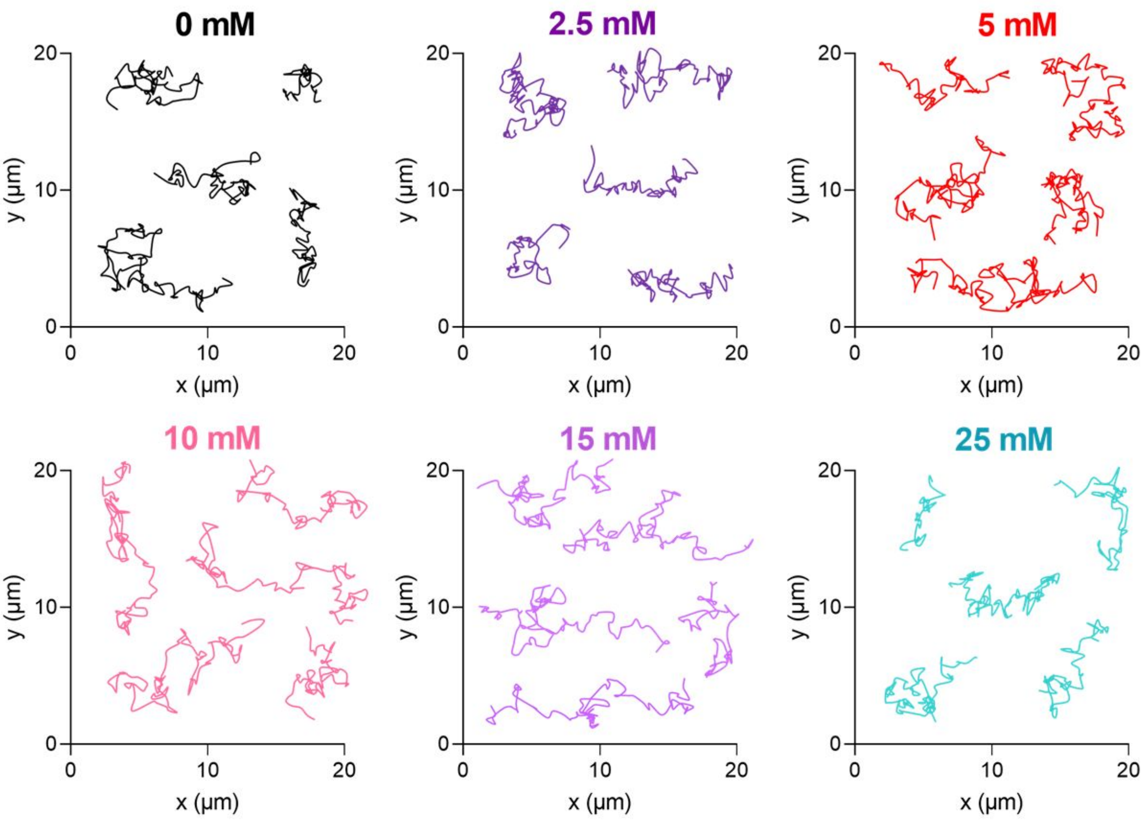

Figure S13. Trajectories of $NM_{Doxo\text{-}GOx}$ in the presence of different concentrations of glucose: 0, black; 2.5, purple; 5, red; 10, pink; 15, lilac; and 25 mM, blue. n = 5 representative nanomotors between 250 and 350 nm in size. The paths followed by the nanomotors were reconstructed by an in-house developed R-method from videos recorded in the Nanosight NS300 sample chamber.

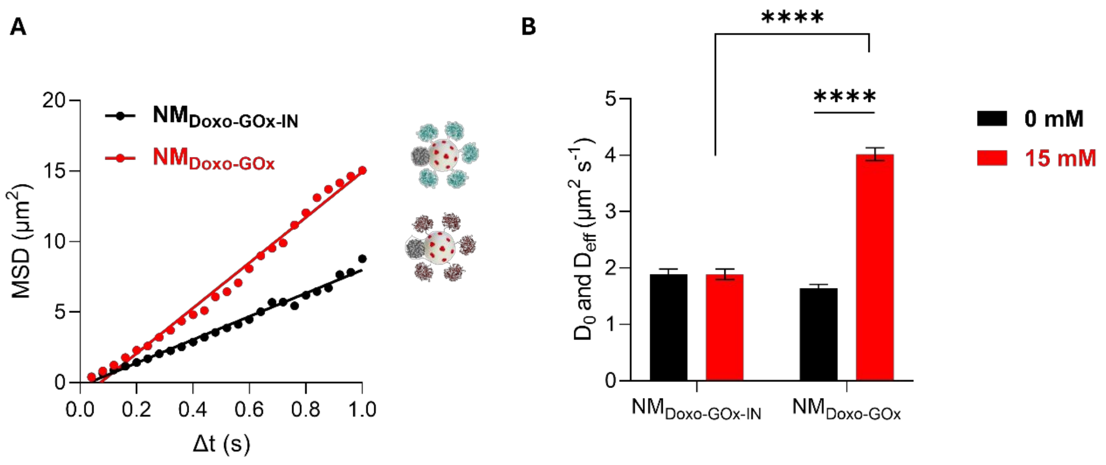

Figure S14. Comparison of the diffusion of $NM_{Doxo\text{-}GOx\text{-}IN}$ and $NM_{Doxo\text{-}GOx}$ at glucose 15 mM. A) MSD *vs* Δt ($NM_{Doxo\text{-}GOx\text{-}IN}$: black, $NM_{Doxo\text{-}GOx}$: red). B) D_0 and D_{eff} (black: 0

glucose, red: glucose 15 mM). Data extracted from NTA analysis (n = 50, size 250-350 nm). Statistical significance was determined by a two-way ANOVA. **** $p < 0.0001$.

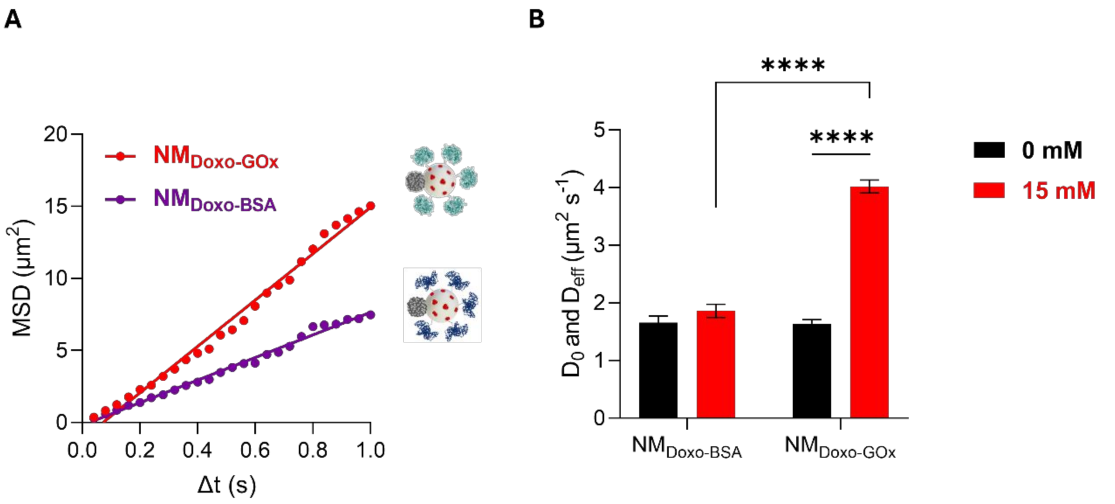

Figure S15. Comparison of the diffusion of $NM_{Doxo\text{-}BSA}$ and $NM_{Doxo\text{-}GOx}$ at glucose 15 mM. A) MSD *vs* Δt ($NM_{Doxo\text{-}BSA}$: purple, $NM_{Doxo\text{-}GOx}$: red). B) D_0 and D_{eff} (black: 0 glucose, red: glucose 15 mM). Data extracted from NTA analysis (n = 50, size 250-350 nm). Statistical significance was determined by a two-way ANOVA. **** $p < 0.0001$.

Table S6. Summary of diffusion coefficients of $NM_{Doxo\text{-}GOx}$, $NM_{Doxo\text{-}BSA}$ and $NM_{Doxo\text{-}GOx\text{-}IN}$ in the absence and presence of biofuel (glucose 15 mM). Calculated from NTA analysis.

Nanoparticle	D_0 in absence of glucose ($\mu m^2\ s^{-1}$)	D_{eff} in glucose 15 mM ($\mu m^2\ s^{-1}$)
**$NM_{Doxo\text{-}BSA}$**	1.4 ± 0.1	1.6 ± 0.1
**$NM_{Doxo\text{-}GOx\text{-}IN}$**	1.9 ± 0.1	2.1 ± 0.1
**$NM_{Doxo\text{-}GOx}$**	**1.6 ± 0.1**	**4.0 ± 0.1**

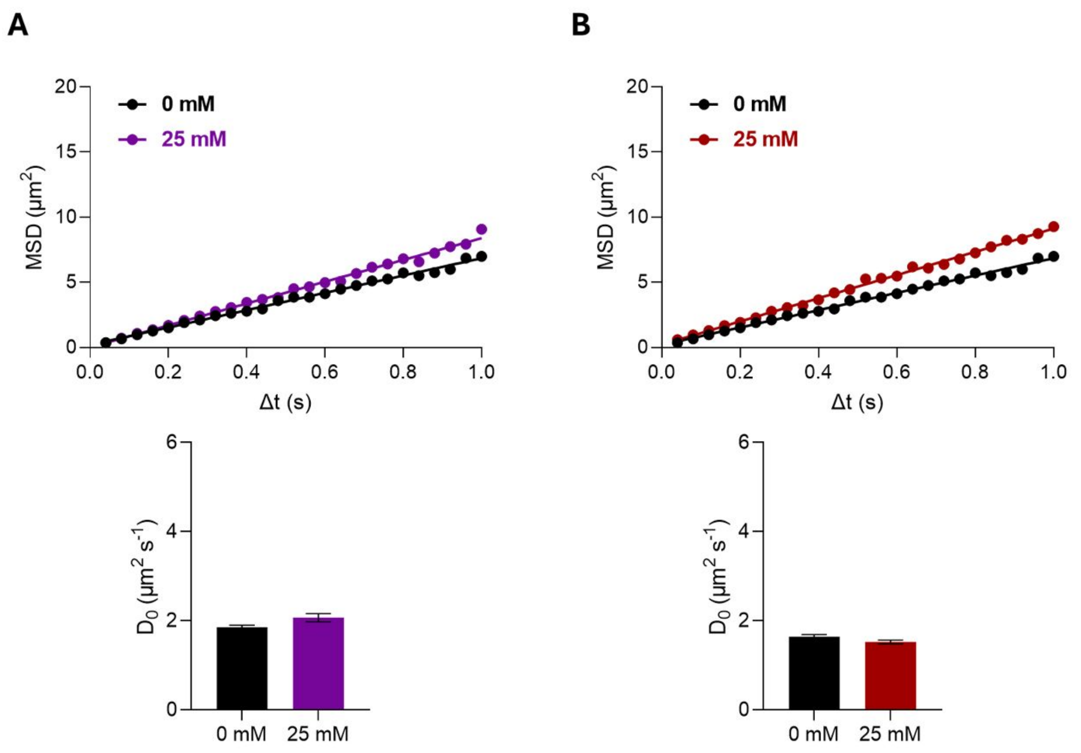

Figure S16. Motion analysis (MSD and D_0 coefficients) of controls (A) $NM_{Doxo\text{-}BSA}$, (B) $NM_{Doxo\text{-}GOx\text{-}IN}$ in response of glucose 25 mM. Data extracted from NTA analysis ($n \geq 30$, size 250-350 nm). Differences were not statistically significant and, therefore, were not indicated.

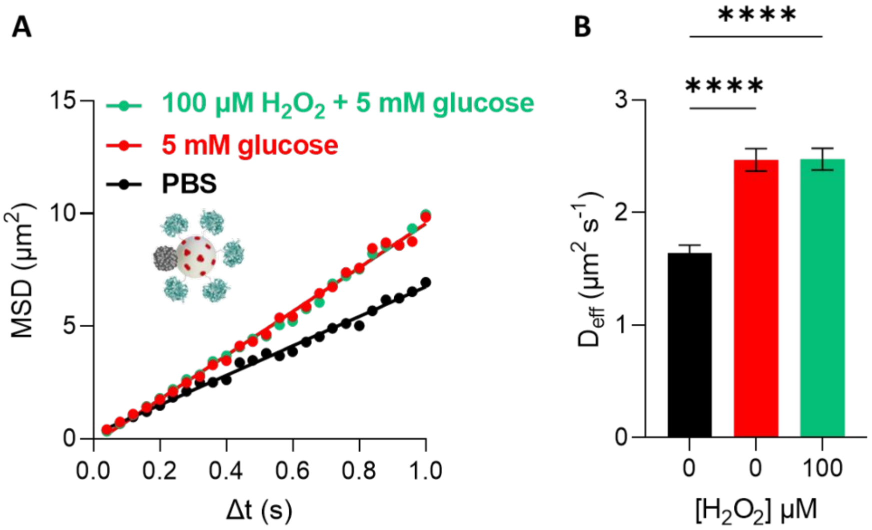

Figure S17. Analysis of the effect of H_2O_2 addition on glucose-induced movement of $NM_{Doxo\text{-}GOx}$. A) MSD *vs* Δt. B) D_0 and $D_{eff.}$ Data were obtained by NTA analysis, n = 50 nanomotors between 250 and 350 nm in size (black: PBS, red: glucose 5 mM, green:

glucose 5 mM + H_2O_2 100 μm). Statistical significance was determined by a one-way ANOVA. **** $p < 0.0001$.

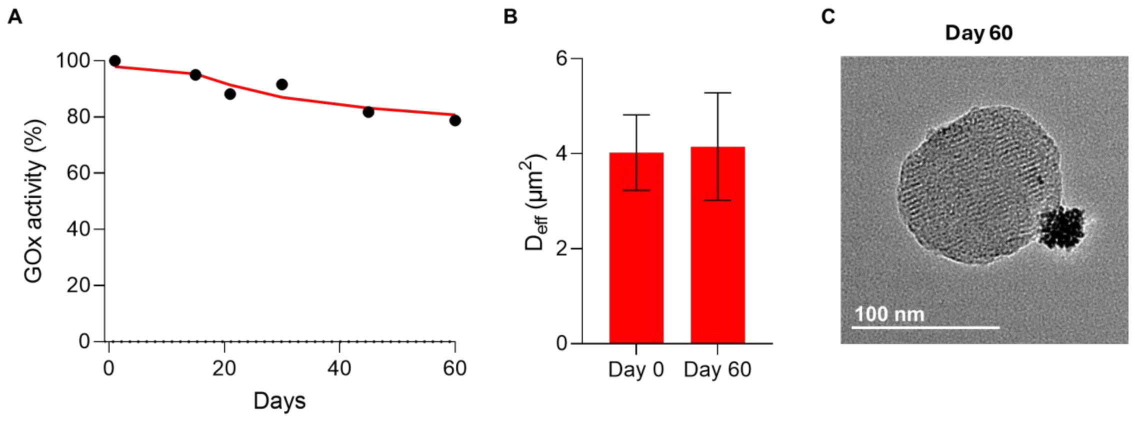

Figure S18. Evaluation of the long-term $NM_{Doxo-GOx}$ stability (60 days). A) Monitorization of GOx activity over time. B) D_{eff} at days 0 and 60 (by NTA, n = 50 nanodevices, data represents the mean ± SD). C) TEM image on day 60. $NM_{Doxo-GOx}$ was stored suspended in PBS at 4 °C.

3. Cell assays

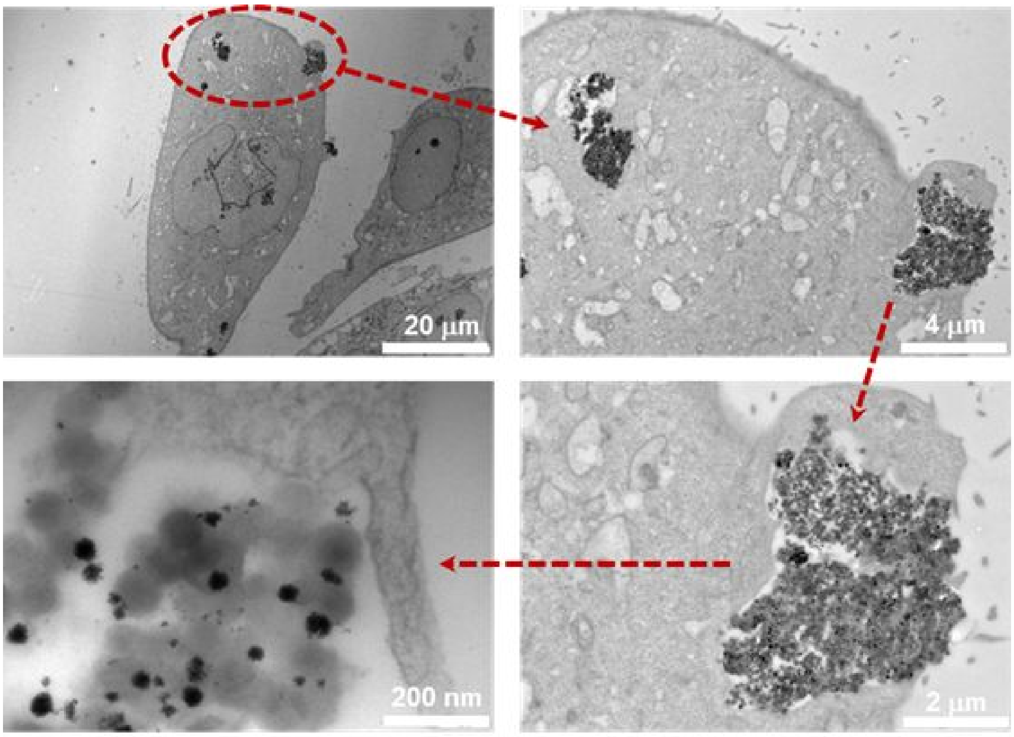

Figure S19. Cell uptake of NM_{GOx} in HeLa cells in 2D culture evaluated by TEM after incubation for 24 h at a concentration of 50 µg mL^{-1}. The images belong to the same cell and show the process of cell internalization at different magnifications (following the red arrow). As can be seen, the nanomotors are located in the lysosomes, where the proteolytic enzymes are located.

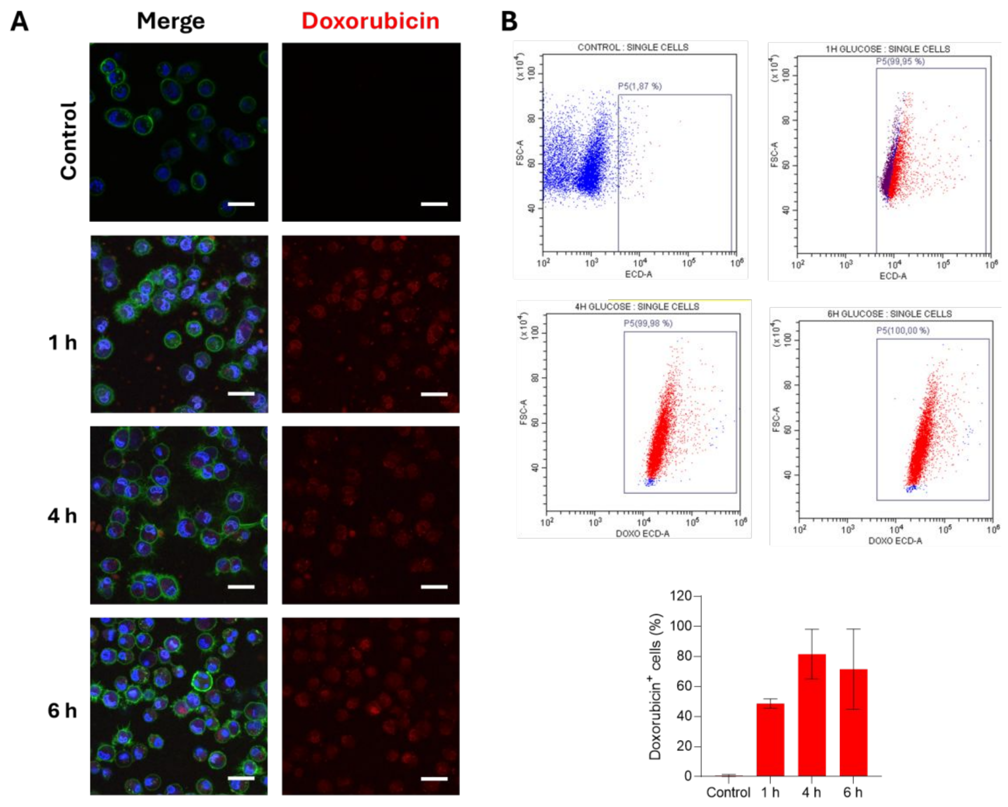

Figure S20. Cell uptake of $NM_{Doxo\text{-}GOx}$ in HeLa cells in 2D culture. A) Evaluated by CLSM (blue: Hoechst nuclear stain, green: WGA membrane stain, red: doxorubicin). Scale bar 20 µm. B) Evaluated by flow cytometry. Cells were treated with 50 µg mL^{-1} and incubated for 1, 4 or 6 h. $NM_{Doxo\text{-}GOx}$ is internalized and release doxorubicin in HeLa cells.

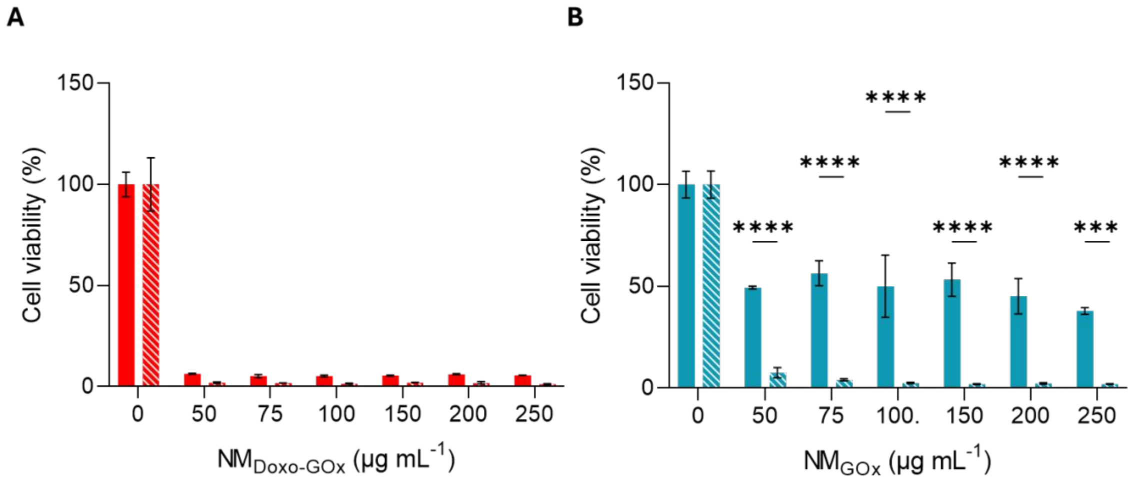

Figure S21. Effect of higher doses of $NM_{Doxo\text{-}GOx}$ (A, red) and NM_{GOx} (B, blue) on the viability of HeLa cells in 2D culture at concentrations from 50 to 250 µg mL^{-1}, in presence

(stripped) and absence (non-stripped) of glucose. Statistical significance was determined by a two-way ANOVA, (** $p < 0.01$, **** $p < 0.0001$, not statistically significant differences between adding or not fuel were not indicated).

A remarkable cytotoxic effect was observed after treatment with high concentrations of $NM_{Doxo-GOx}$ both in the presence and absence of glucose, suggesting that the nanomotor efficiently delivers doxorubicin to cancer cells. In contrast, treatment with NM_{GOx} (the unloaded control nanomotor) at high concentrations only caused pronounced cell death in the presence of glucose. This result suggests that the induction of ROS by the catalytic activities present in the nanomotor (GOx and Pt) causes cell damage by oxidative stress at these concentrations.

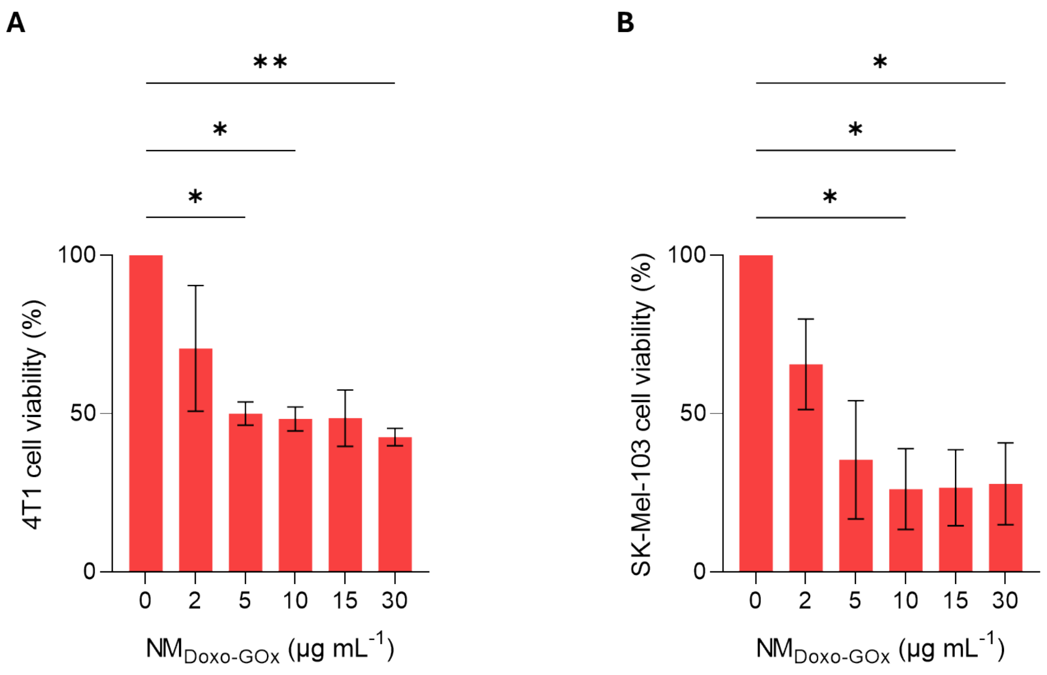

Figure S22. Cell viability assays of 4T1 (A) and SK-Mel-103 (B) cells treated with $NM_{Doxo-GOx}$ at concentrations from 2 to 30 $\mu g\ mL^{-1}$ in presence of glucose ($n = 3$). Statistical significance was determined by a one-way ANOVA, (* $p < 0.1$, ** $p < 0.01$, not statistically significant differences were not indicated).

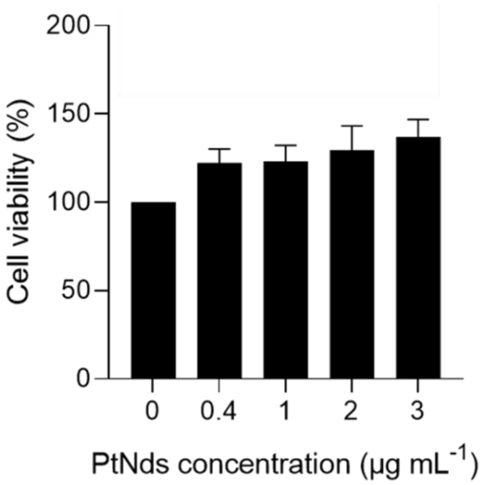

Figure S23. Effect of PtNds on the viability of HeLa cells in 2D culture at concentrations from 0.4 to 3 µg mL^{-1} (equivalent to J-Pt at 2-15 µg mL^{-1}, as indicated by ICP-MS). Assessed by WST-1 assay, (n = 2). Differences were not statistically significant and, therefore, were not indicated.

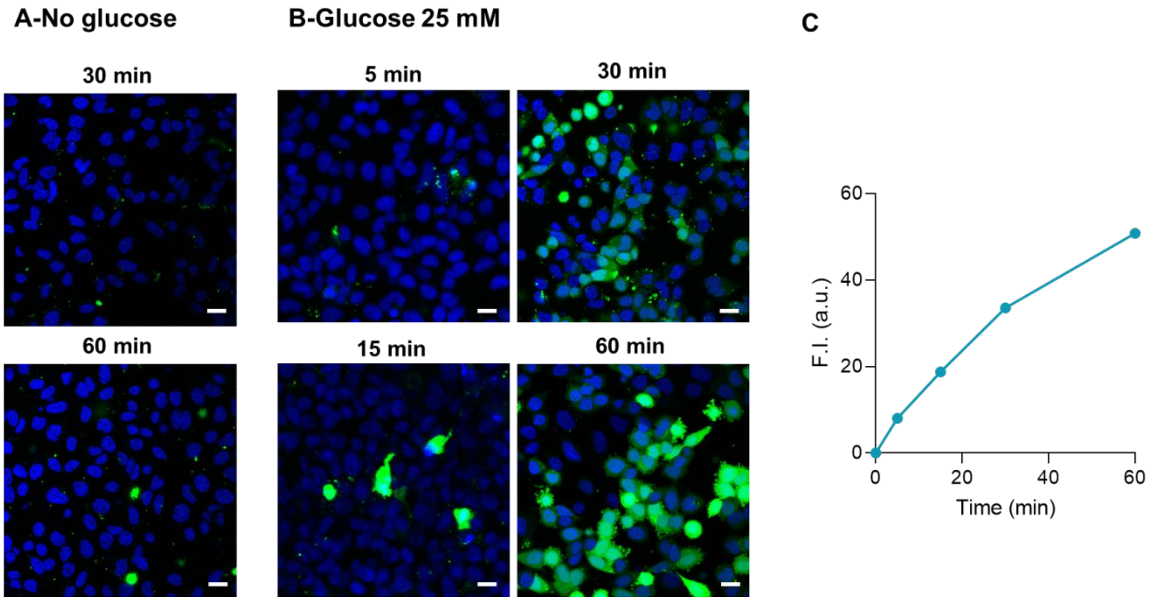

Figure S24. Intracellular ROS production by using DCFDA-H2DCFDA as detection probe. HeLa cells were treated with 50 µg mL^{-1} of NM_{GOx} in the absence (A) or presence of glucose (25 mM) at different incubation times: 5, 15, 30 and 60 min (B). C) Quantification of the DCF fluorescent signal at each incubation time. Cell nuclei, blue; DCF oxidized by ROS, green; scale bar 20 µm. Point that in this case NM_{GOx} was used instead of $NM_{Doxo\text{-}GOx}$ to avoid drug toxicity induced by doxorubicin.

As observed in the CLSM images, NM_{GOx} only produced ROS in HeLa cells if incubated with glucose. ROS production was fast, since in only 5 min the DCF-signal was already detected.

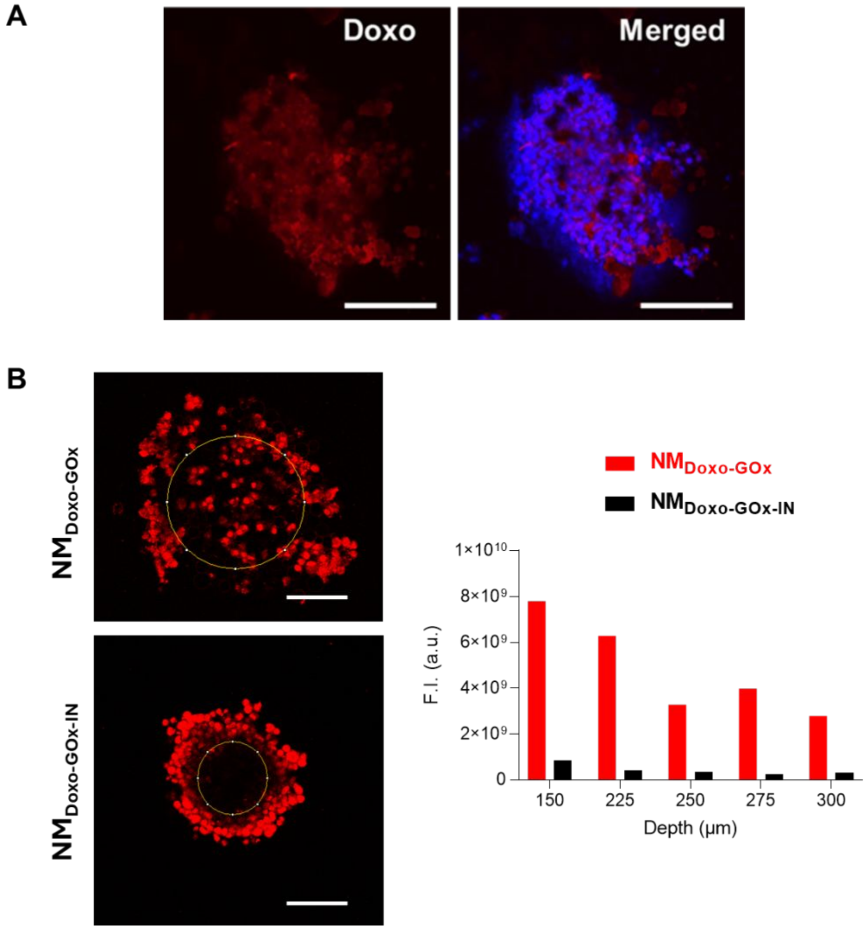

Figure S25. A) $NM_{Doxo-GOx}$ doxorubicin release (red) in 3D HeLa spheroids analyzed by CLSM (blue, Hoechst nuclear stain), scale bar 200 µm. Spheroids were treated with $NM_{Doxo-GOx}$ for 4 h at 100 µg mL^{-1}. B) Analysis of doxorubicin distribution in the core of 3D HeLa spheroids sections after treatment with $NM_{Doxo-GOx}$ or $NM_{Doxo-GOx-IN}$. The analyzed areas correspond to the core of each spheroid and are marked with a circle in the representative images (depth: 150 µm). Quantification of fluorescence intensity (FI) was assessed with Image J and expressed as FI x area. Scale bar: 1 mm.

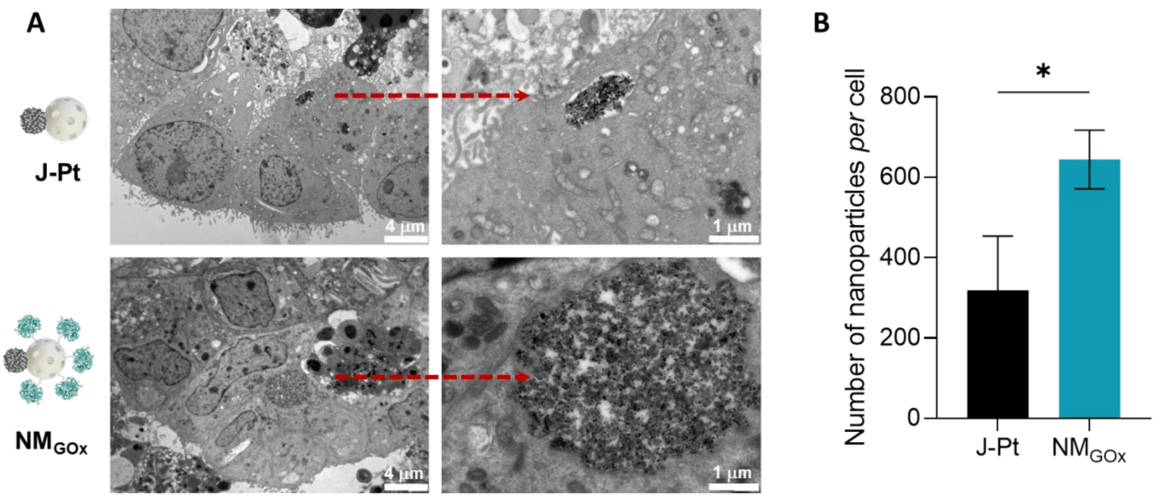

Figure S26. Comparison of nanomotors NM_{GOx} and J-Pt penetration into 3D HeLa spheroids. Nanodevices were incubated for 4 h at 150 µg mL^{-1}. A) TEM images (scale bars: 4 µm and 1 µm, following the red arrow). B) Subsequent quantification of the number of nanoparticles *per* cell using Image J (quantifying at least 9 images *per* group). Data are expressed as mean ± SEM, n = 3. Statistical significance was determined by a one-way ANOVA (* $p < 0.05$).

4. *In vivo* antitumoral effect of nanomotors

For the early *in vivo* antitumor assays were carried out using three nanoparticles: i.e. the final nanomotor ($NM_{Doxo\text{-}GOx}$), the unloaded control (NM_{GOx}), and one of the controls that did not exhibit self-propulsion ($NM_{Doxo\text{-}BSA}$). We did not include more experimental groups for ethical reasons to reduce the number of animals required. The characterization (quantity of drug loaded and gatekeeper quantity and activity) of the selected nanoparticles is shown in **Table S4**. The dose of nanoparticles was adjusted according to the amount of doxorubicin loaded in $NM_{Doxo\text{-}GOx}$.

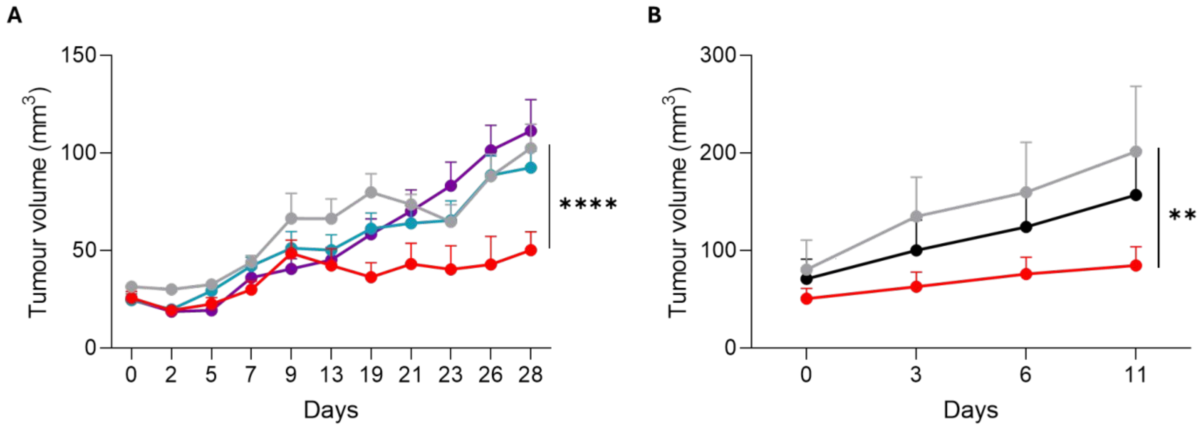

Figure S27. Monitorization of tumor volume (mm^3) upon treatment with $NM_{Doxo\text{-}GOx}$ or control nanoparticles in early (A) and advanced (B) tumor models (vehicle, gray; $NM_{Doxo\text{-}GOx}$, red; NM_{GOx}, blue; $NM_{Doxo\text{-}BSA}$, purple; and $NM_{Doxo\text{-}GOx\text{-}IN}$, black). Statistical significance was determined by a one-way ANOVA, (** $p < 0.01$, **** $p < 0.0001$, not statistically significant differences were not indicated).

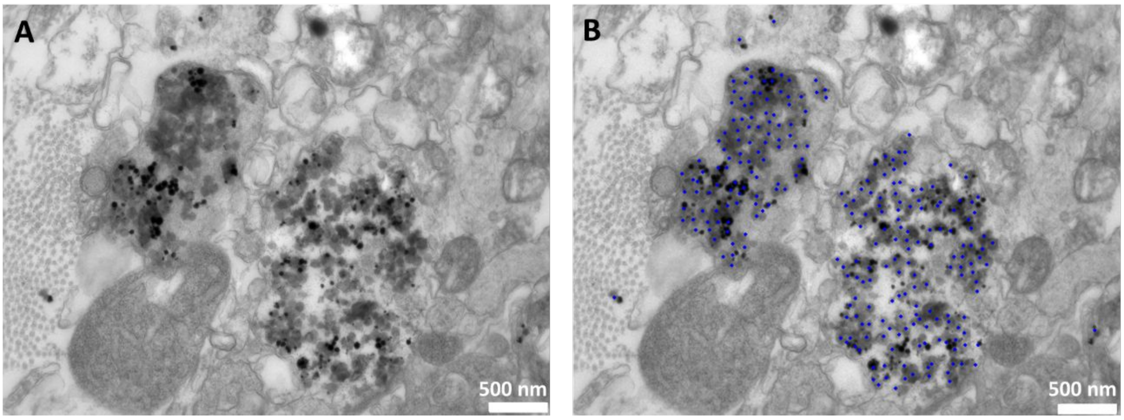

Figure S28. Representative images of the procedure followed (cell counter ImageJ plugging) for quantification of the number of nanoparticles *per* cell in spheroids and tumors. A) Tumor treated with $NM_{Doxo\text{-}GOx}$. B) Detected nanoparticles marked in blue. Scale bar: 500 nm.

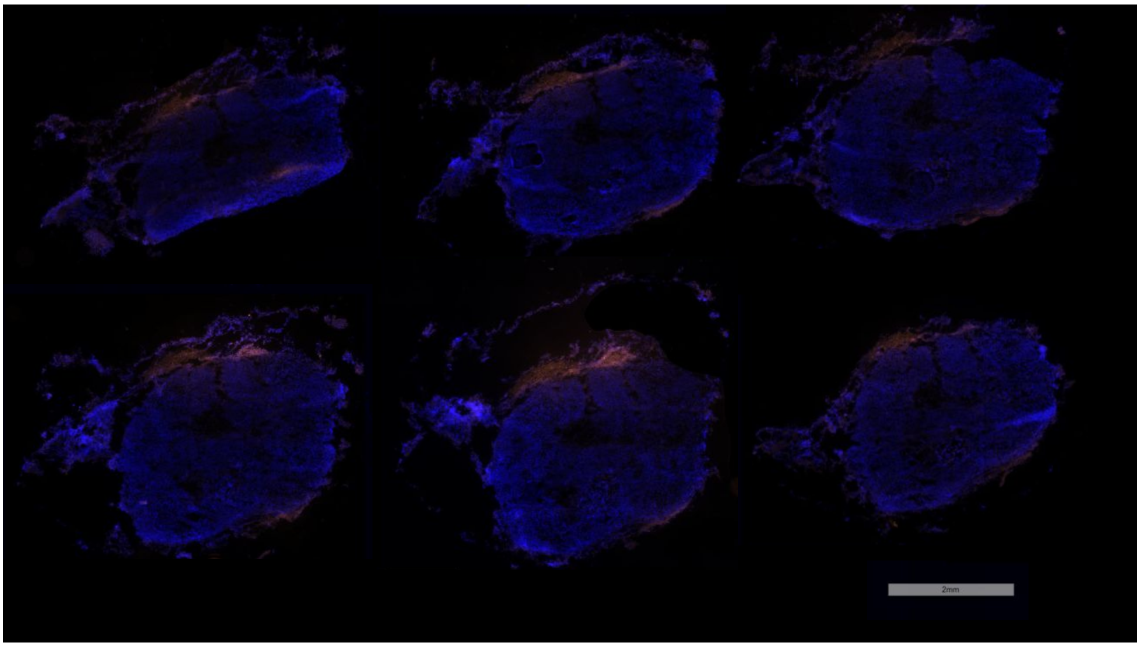

Figure S29. Representative images of complete tumor slides used for quantifying the doxorubicin delivery in $NM_{Doxo\text{-}GOx}$ with the APERIO Image Scope Software. All slides belong to the same tumor. Blue: Hoechst nuclear stain; orange: doxorubicin. (scale bar: 2 mm).

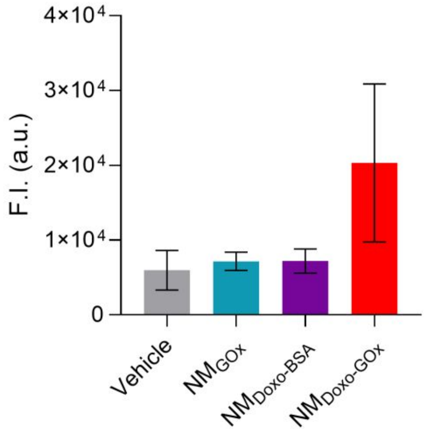

Figure S30. *In vivo* ROS production in tumor-bearing Balb/c mice by using DCFDA-H2DCFDA as detection probe. Treatments: $NM_{Doxo\text{-}GOx}$ (red), NM_{GOx} (blue), $NM_{Doxo\text{-}BSA}$ (purple) and vehicle gray. Data is expressed as mean ± SEM, n = 4.

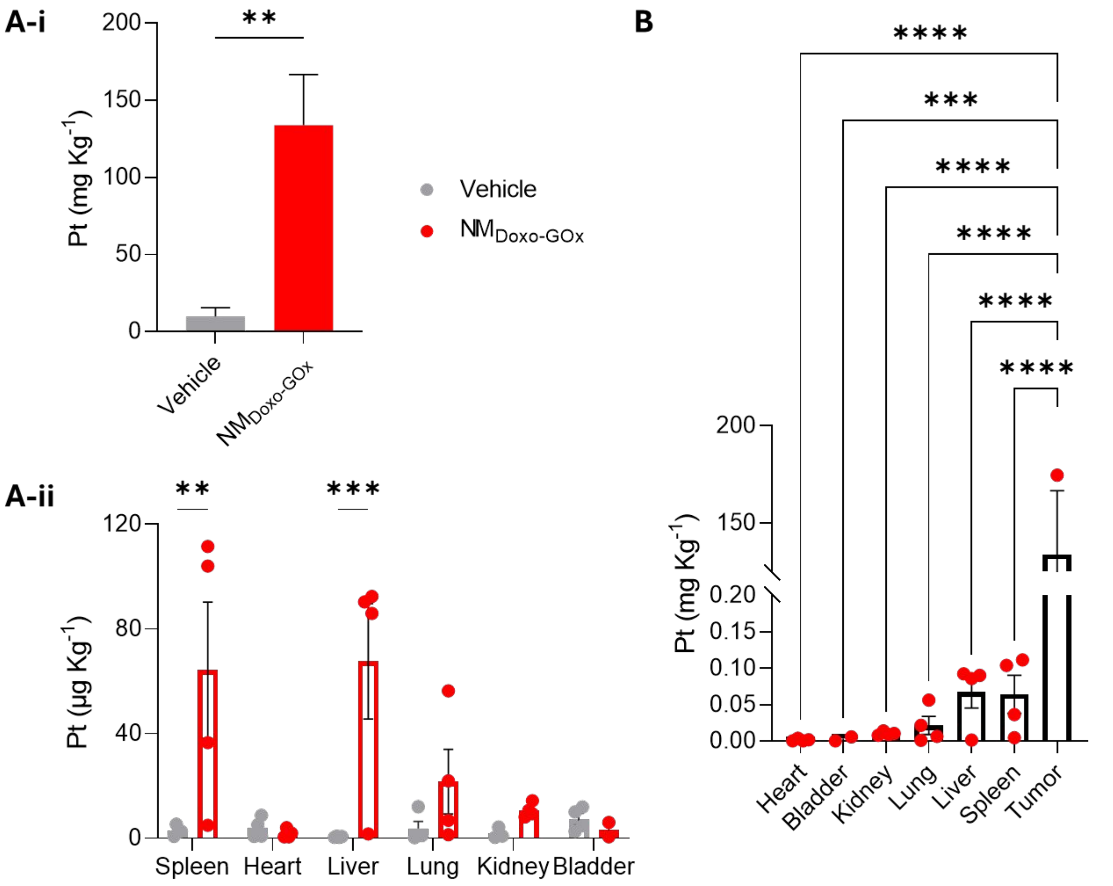

Figure 31. Analysis of $NM_{Doxo\text{-}GOx}$ biodistribution in organs (heart, bladder, kidney, lungs, liver, spleen and tumor) of early-stage cancer model mice. Pt levels were analyzed

by Inductively Coupled Plasma Mass Spectroscopy (ICP-MS). Data are expressed as mean ± SEM (n = 4) and represented as mg or μg Pt *per* Kg of tissue. A) Comparison of Pt levels in mice (A-i, tumor; A-ii, other organs) treated with vehicle (gray) or with $NM_{Doxo\text{-}GOx}$ (red). B) Biodistribution of Pt in organs of mice treated with $NM_{Doxo\text{-}GOx}$. * $p < 0.05$, ** $p < 0.001$, *** $p < 0.001$.

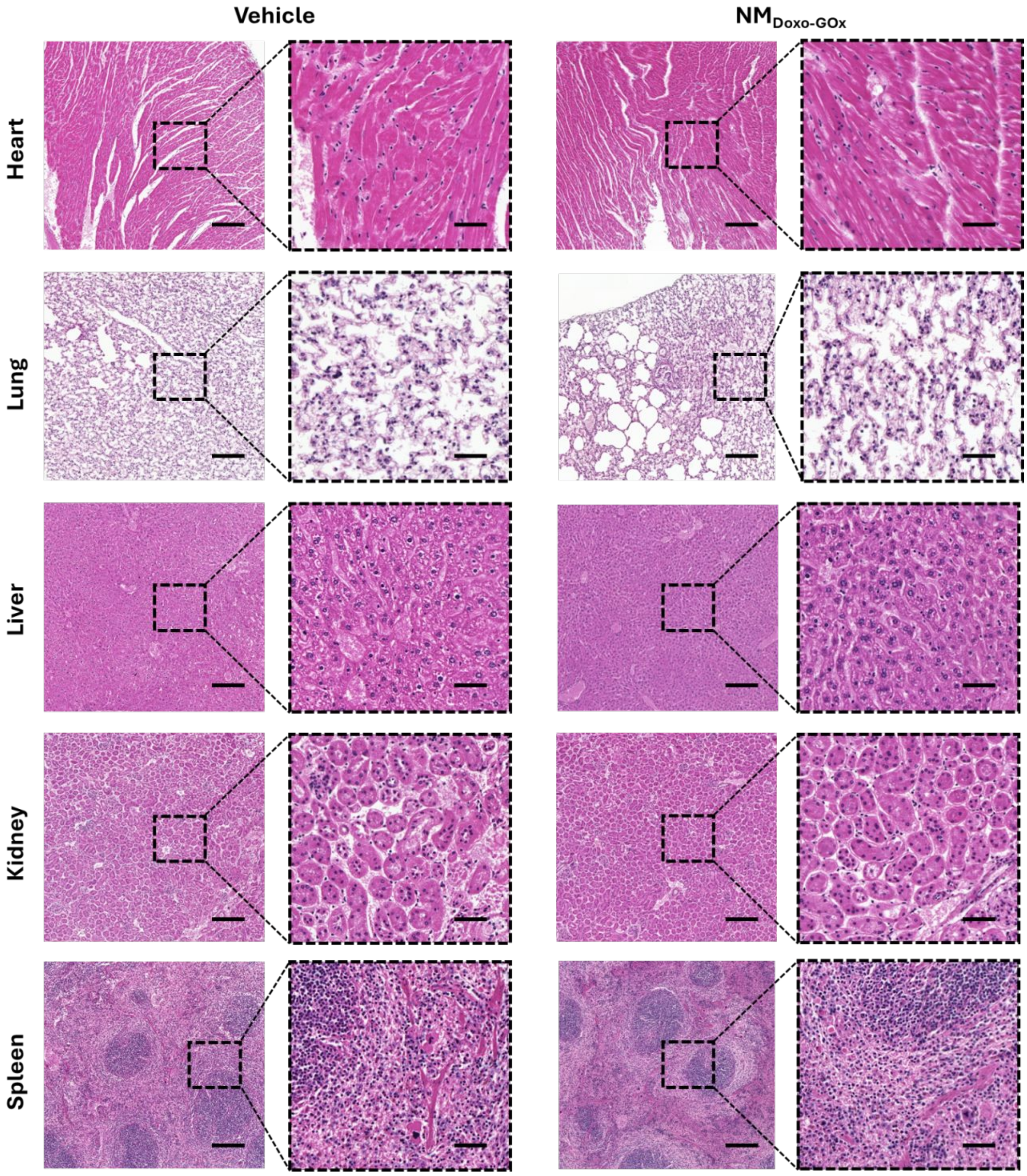

Figure S32. Evaluation of biosafety of $NM_{Doxo\text{-}GOx}$ in mice (I). Hematoxylin and eosin staining of mice organs (heart, lung, liver, kidney and spleen) after treatment with vehicle (left) or $NM_{Doxo\text{-}GOx}$ (right). Scale bars = 200 μm and 50 μm (zoomed image).

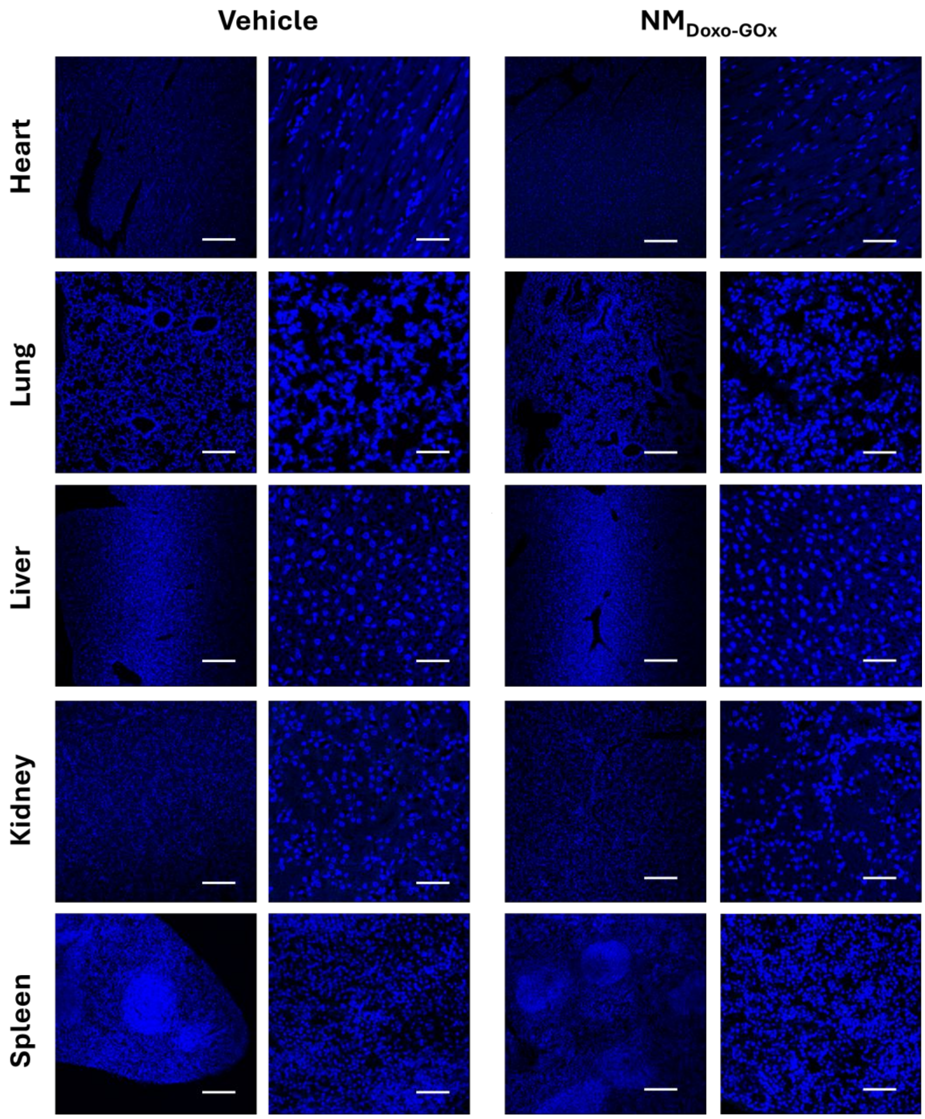

Figure S33. Evaluation of biosafety of $NM_{Doxo\text{-}GOx}$ in mice (II). Evaluation of apoptosis by TUNEL assay in mice organs (heart, lung, liver, kidney and spleen) after treatment with vehicle (left) or $NM_{Doxo\text{-}GOx}$ (right). Blue: DAPI nuclear stain; green: apoptotic cells. Scale bars = 200 µm (left) and 50 µm (right, zoomed image). No apoptotic cells were observed in any of the organs studied.

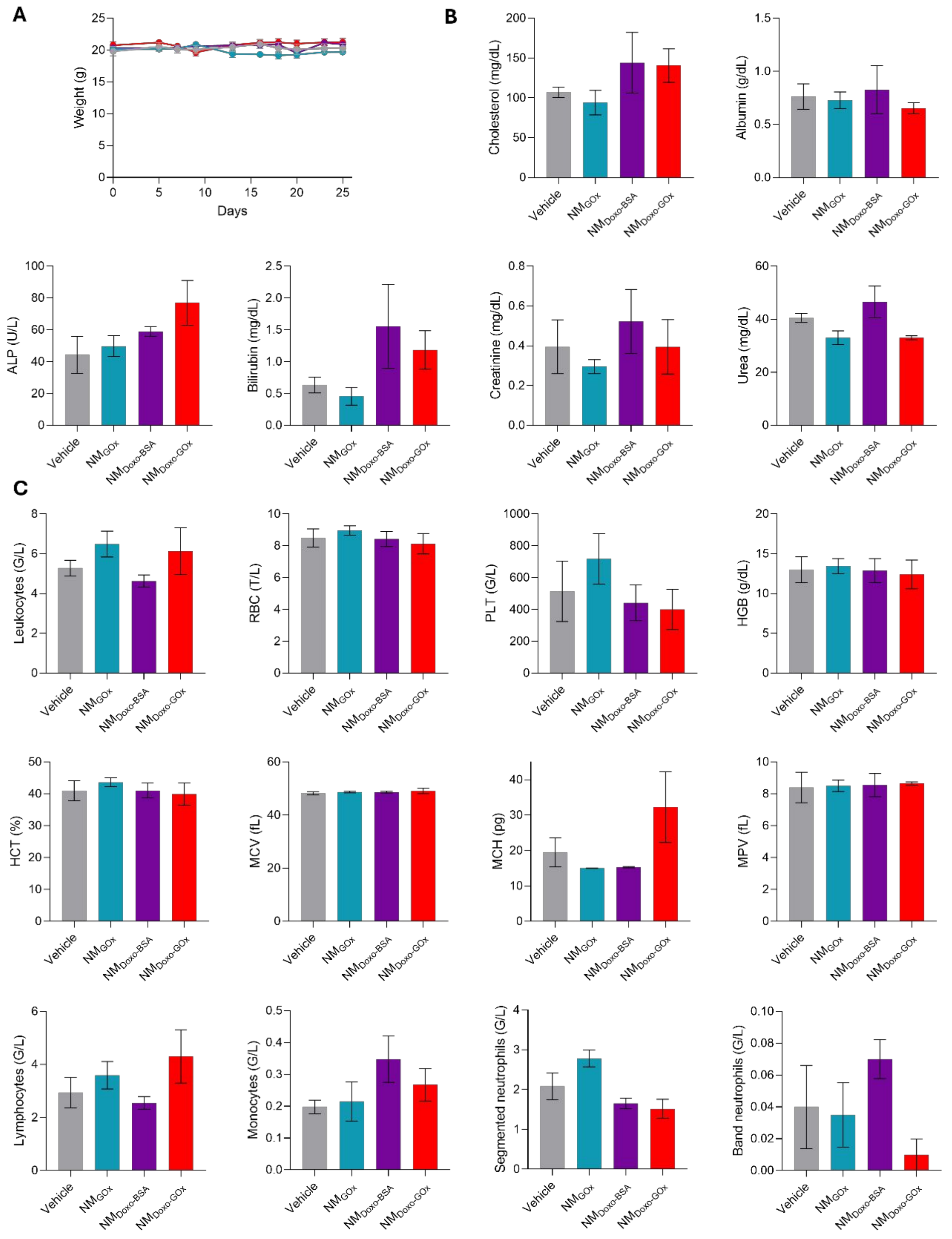

Figure S34. Evaluation of the biosafety of the nanoparticles ($NM_{Doxo\text{-}GOx}$, red; NM_{GOx}, blue; $NM_{Doxo\text{-}BSA}$, purple; and vehicle, gray) in mice (III). A) Body weight variation from the beginning to the end of treatments (n = 6). B) Biochemical analysis in mice blood samples at the end of the study, (mean ± SEM, n = 4) including cholesterol (mg dL^{-1}) and albumin (g dL^{-1}) levels, hepatic function biomarkers (alkaline phosphatase (ALP, U L^{-1}) and bilirubin (mg dL^{-1})), and kidney function biomarkers (creatinine (CREA, mg dL^{-1})

and urea (dg dL^{-1})). C) Hematological count in xenograft blood samples after the treatments, (mean ± SEM, n = 4) including leukocytes (g L^{-1}), red blood cells (RBC, T L^{-1}), platelets (PLT, g L^{-1}), hemoglobin (HGB, g dL^{-1}), hematocrit (HCT, %), mean corpuscular volume (MCV, fL), mean corpuscular-hemoglobin (MCH, pg), mean platelet volume (MPV, fL), lymphocytes (g L^{-1}), monocytes (g L^{-1}), segmented neutrophils (g L^{-1}) and band neutrophils (g L^{-1}).

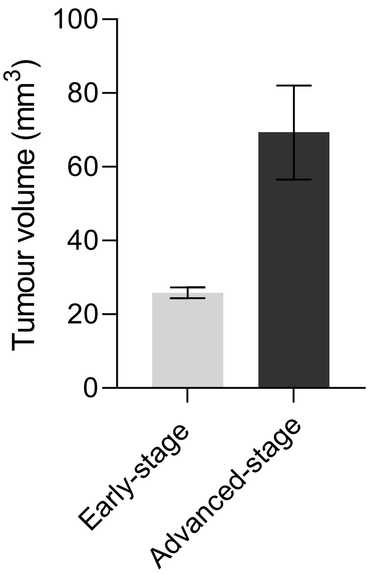

Figure S35. Comparative of tumor volumes at treatment initiation in the early-stage (gray) and the advanced-stage (black) cancer models. In the early-stage model, the nanodevices were injected when tumor volume reached 25 mm^3 (referred as day 0 in **Figure 5**B), while in the advanced-stage model treatments began when tumor volume reached 70 mm^3 (referred as day 0 in **Figure 7**B).

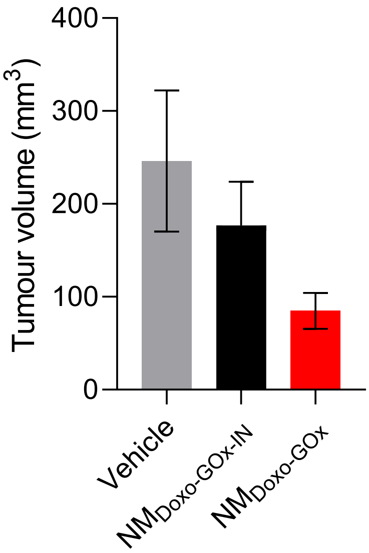

Figure S36. Comparative of tumor volumes (mm^3, Equation 9) at the end of the experiment (day 11) in the advanced-stage cancer model (n = 9 tumors) for treatments with vehicle, $NM_{Doxo\text{-}GOx\text{-}IN}$ and $NM_{Doxo\text{-}GOx}$. The tumor size of vehicle-treated mice compromised their well-being, and the experiment was terminated at this point.

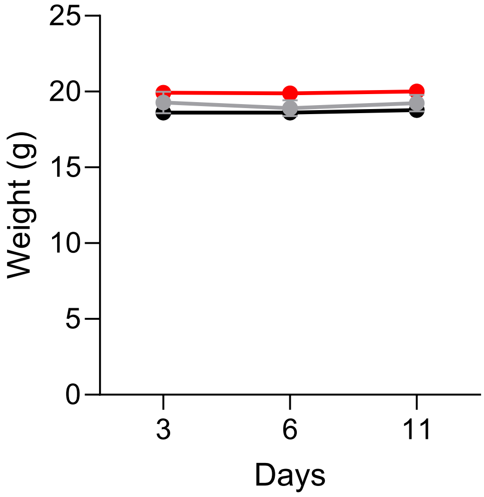

Figure S37. Mice body weight variation from the beginning to the end of treatments in the advanced-stage cancer model. $NM_{Doxo\text{-}GOx}$, red; $NM_{Doxo\text{-}GOx\text{-}IN}$, black; and vehicle, gray, n = 5 mice.

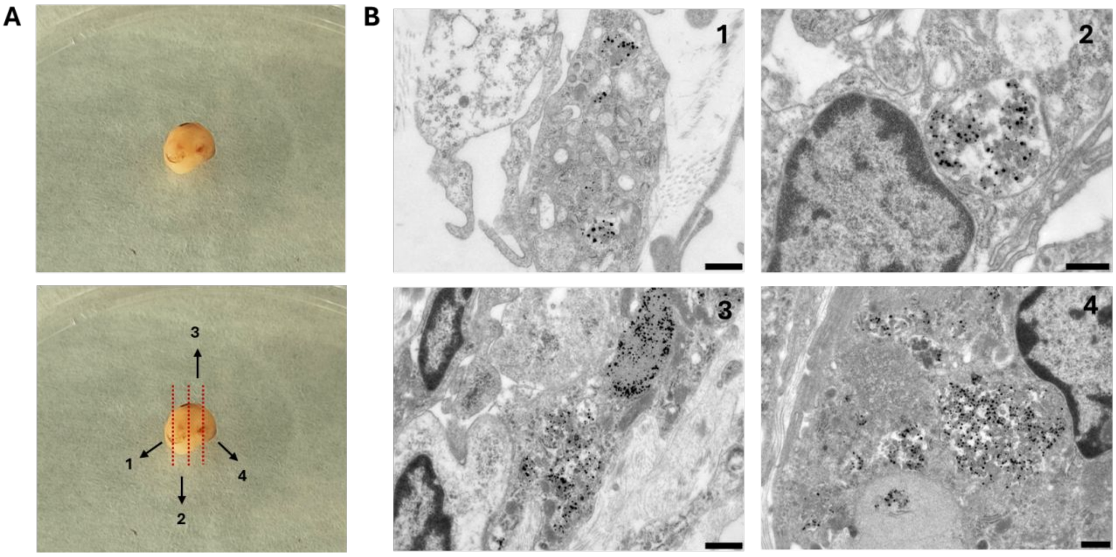

Figure S38. $NM_{Doxo\text{-}GOx}$ distributes throughout tumor tissue in the advanced-stage cancer model. A complete tumor of a mouse treated with $NM_{Doxo\text{-}GOx}$ was divided into 4 representative areas (A) and each of them was evaluated by TEM (B). Scale bar: 500 nm.

Nanoparticles inside tumor cells were localized in all representative tumor sections, suggesting that nanomotors are able to penetrate and distribute throughout the entire tumor tissue. At least 10 images were analyzed in each of the representative areas (1-4).

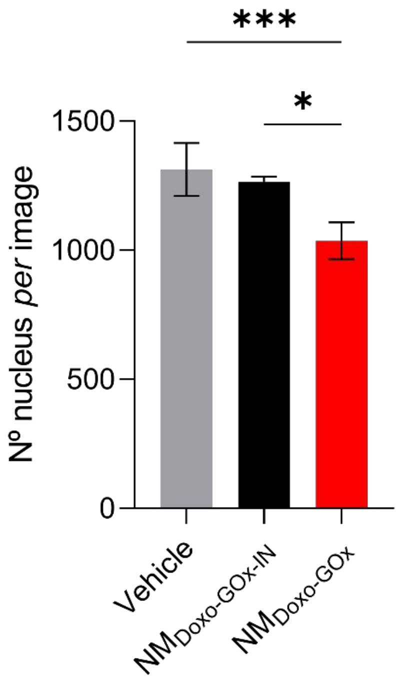

Figure S39. Comparative of the number of cell nuclei in tumor sections of mice treated with vehicle, $NM_{Doxo\text{-}GOx\text{-}IN}$ or $NM_{Doxo\text{-}GOx}$ in the advanced-cancer model. Tumors treated with the active nanomotor displayed a significant decrease in cell number due to doxorubicin delivery and apoptosis induction. Statistical significance was determined by a one-way ANOVA test (* $p < 0.05$, *** $p < 0.001$).

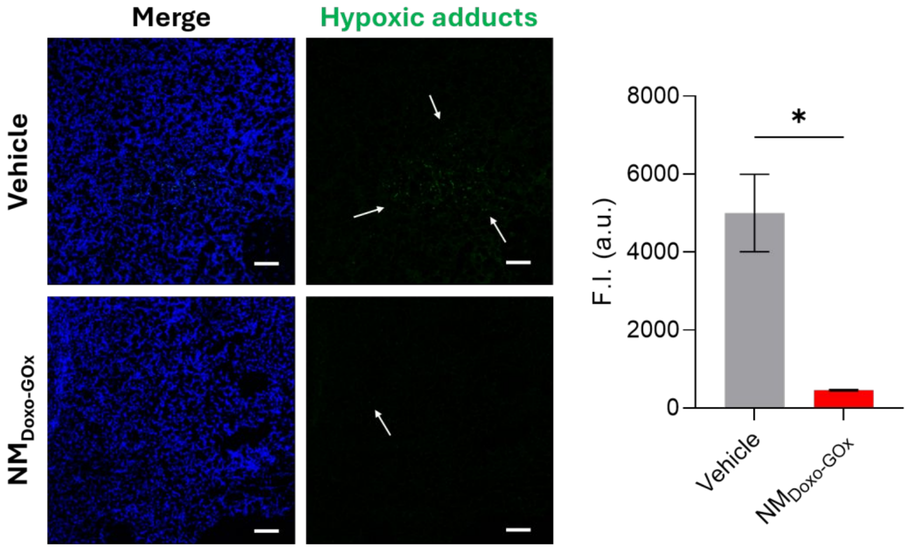

Figure S40. Comparative of hypoxia levels in tumor sections of mice treated with vehicle or $NM_{Doxo\text{-}GOx}$ in the advanced-cancer model. Blue: DAPI nuclear stain; green: hypoxic adducts marked with an Alexa Fluor-488 antibody. Scale bars: 100 μm. Statistical significance was determined by a one-way ANOVA (* $p < 0.05$). Tumors treated with $NM_{Doxo\text{-}GOx}$ display a significant decrease in hypoxic adducts, suggesting that the activity of $NM_{Doxo\text{-}GOx}$ contributes to the reversal of hypoxia.

5. Patient-derived organoids experiments

Table S7. PDO's culture media composition.

Reagent	Final concentration
Advanced-DMEM F-12 (Biowest, France)	N/A
Fetal bovine serum (GIBCO, USA)	5 % (v/v)
L-glutamine (Biowest, France)	1 % (v/v)

HEPES (Gibco, USA)	10 μM
Gentamicin (Gibco, USA)	50 μg/mL
Human EGF (Merck, Germany)	10 ng/mL
Hydrocortisone (MedChemExpress, USA)	1 μg/mL
Y-27632 (Stemcell Technologies, Canada)	10 μM

6. Supplementary videos

Video S1. Brownian motion of $NM_{Doxo\text{-}GOx.}$

Video S2. Enhanced diffusion of $NM_{Doxo\text{-}GOx}$ in the presence of glucose 25 mM.

7. References

(1) Yue, L.; Yang, K.; Li, J.; Cheng, Q.; Wang, R. Self-Propelled Asymmetrical Nanomotor for Self-Reported Gas Therapy. *Small* **2021**, *17* (34), 2102286.

(2) Wan, M.; Liu, Z.; Li, T.; Chen, H.; Wang, Q.; Chen, T.; Tao, Y.; Mao, C. Zwitterion-Based Hydrogen Sulfide Nanomotors Induce Multiple Acidosis in Tumor Cells by Destroying Tumor Metabolic Symbiosis. *Angew. Chem. Int. Ed.* **2021**, *60* (29), 16139-16148.

(3) Yu, W.; Lin, R.; He, X.; Yang, X.; Zhang, H.; Hu, C.; Liu, R.; Huang, Y.; Qin, Y.; Gao, H. Self-Propelled Nanomotor Reconstructs Tumor Microenvironment through Synergistic Hypoxia Alleviation and Glycolysis Inhibition for Promoted Anti-Metastasis. *Acta Pharm. Sin. B* **2021**, *9*, 2924-2936.

(4) Zhang, Z.; Zhang, D.; Qiu, B.; Cao, W.; Liu, Y.; Liu, Q.; Li, X. Icebreaker-Inspired Janus Nanomotors to Combat Barriers in the Delivery of Chemotherapeutic Agents. *Nanoscale* **2021**, *13* (13), 6545-6557.

(5) Wan, M. M.; Chen, H.; Wang, Z. D.; Liu, Z. Y.; Yu, Y. Q.; Li, L.; Miao, Z. Y.; Wang, X. W.; Wang, Q.; Mao, C.; Shen, J.; Wei, J. Nitric Oxide-Driven Nanomotor for Deep Tissue Penetration and Multidrug Resistance Reversal in Cancer Therapy. *Adv. Sci.* **2020**, *8* (3), 2002525.

(6) Chen, H.; Shi, T.; Wang, Y.; Liu, Z.; Liu, F.; Zhang, H.; Wang, X.; Miao, Z.; Liu, B.; Wan, M.; Mao, C.; Wei, J. Deep Penetration of Nanolevel Drugs and Micrometer-Level T Cells Promoted by Nanomotors for Cancer Immunochemotherapy. *J. Am. Chem. Soc.* **2021**, *143* (31), 12025-12037.

(7) Xu, L.; Zhang, K.; Ma, X.; Li, Y.; Jin, Y.; Liang, C.; Wang, Y.; Duan, W.; Zhang, H.; Zhang, Z.; Shi, J.; Liu, J.; Wang, Y.; Li, W. Nanocarrier Cancer Therapeutics with Functional Stimuli-Responsive Mechanisms. *J. Nanobiotechnol.* **2022**, *20* (1), 429.

(8) Chang, X.; Zhu, M.; Tang, X.; Yu, X.; Liu, F.; Chen, L.; Yin, T.; Zhu, Z.; Zhang, Y.; Chen, X. Enhanced Manipulation of Tumor Microenvironments by Nanomotor for Synergistic Therapy of Malignant Tumor. *Biomaterials* **2022**, *290*, 121853.

(9) Zhong, H.; Zhang, Z.; Zhou, Y.; Wu, L.; Ke, P.; Lu, Y.; Dai, Q.; Bao, X.; Xia, Y.; Yang, Q.; Tan, X.; Wei, Q.; Xu, W.; Han, M.; Ma, L. Pt/DOX Nanomotors Enhance Penetration in the Deep Tumor by Positive Chemotaxis. *ACS Appl. Mater. Interfaces* **2022**, *14* (33), 38172-38184.

(10) Cao, Y.; Liu, S.; Ma, Y.; Zu, M.; Sun, J.; Dai, F.; Duan, L.; Xiao, B. Oral Nanomotor-Enabled Mucus Traverse and Tumor Penetration for Targeted Chemo-Sono-Immunotherapy against Colon Cancer. *Small* **2022**, *18* (42), 2203466.

(11) Yang, Z.; Wang, L.; Gao, Z.; Hao, X.; Luo, M.; Yu, Z.; Guan, J. Ultrasmall Enzyme-Powered Janus Nanomotor Working in Blood Circulation System. *ACS Nano* **2023**, *17* (6), 6023-6035.

(12) Jung, Y. C.; Muramatsu, H.; Fujisawa, K.; Kim, J. H.; Hayashi, T.; Kim, Y. A.; Endo, M.; Terrones, M.; Dresselhaus, M. S. Optically and Biologically Active Mussel Protein-Coated Double-Walled Carbon Nanotubes. *Small* **2011**, *7* (23), 3292-3297.

(13) Gao, L.; Zhuang, J.; Nie, L.; Zhang, J.; Zhang, Y.; Gu, N.; Wang, T.; Feng, J.; Yang, D.; Perrett, S.; Yan, X. Intrinsic Peroxidase-Like Activity of Ferromagnetic Nanoparticles. *Nat. Nanotechnol.* **2007**, *2*, 577-583.
